# Supplementary material for: TET1 is a beige adipocyte-selective epigenetic suppressor of thermogenesis
Source: Nat Commun. 2020 Aug 27;11:4313. doi: 10.1038/s41467-020-18054-y (PMC7453011; doi:10.1038/s41467-020-18054-y)
Supplement: Supplementary file 1 — Supplementary Information [file 41467_2020_18054_MOESM1_ESM.pdf]

TET1 is a beige adipocyte–selective epigenetic suppressor of thermogenesis

Villivalam et al.

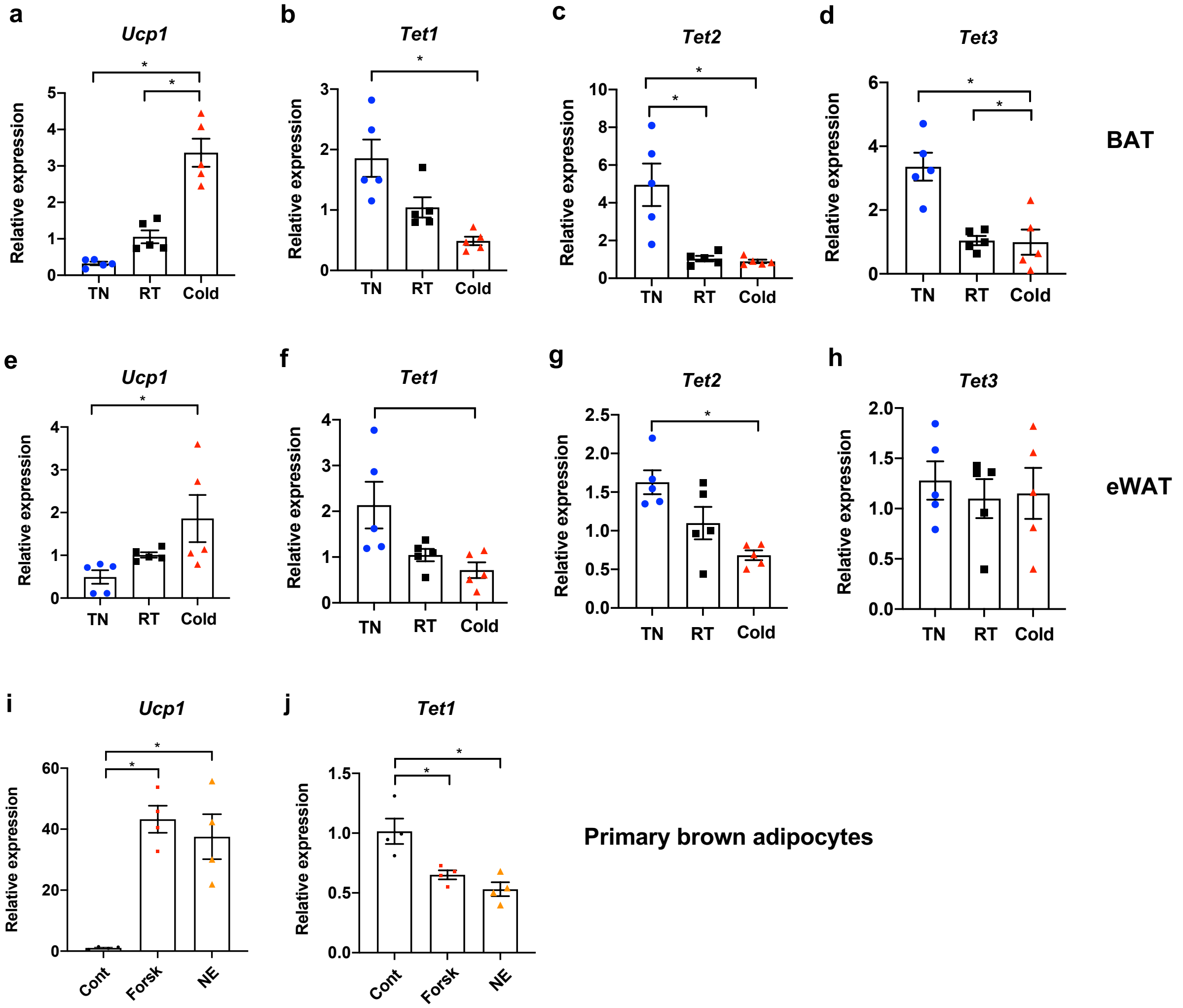

Supplementary Figure 1. *Tet1* expression in BAT and eWAT is regulated by ambient temperature and cAMP signaling. a-h *Tet* and *Ucp1* mRNA expression in BAT **a-d** and eWAT **e-h** from wild-type male C57BL/6J mice housed at room temperature (RT) then exposed to cold or thermoneutrality (TN) for 24 hours ($n = 5$ per group. Data are expressed as means ± SEM. *denotes $p < 0.05$, determined by two-tailed student's t-test and one-way ANOVA followed by Bonferroni post-hoc testing). **i, j** *Tet* and *Ucp1* mRNA expression with and without 1 uM forskolin (Forsk) or 1 uM norepinephrine (NE) stimulation for 3 hours in mature primary brown adipocytes ($n = 4$ per group. Data are expressed as means ± SEM. *denotes $p < 0.05$, determined by two-tailed student's t-test and one-way ANOVA followed by Bonferroni post-hoc testing). Source data are provided as a source data file.

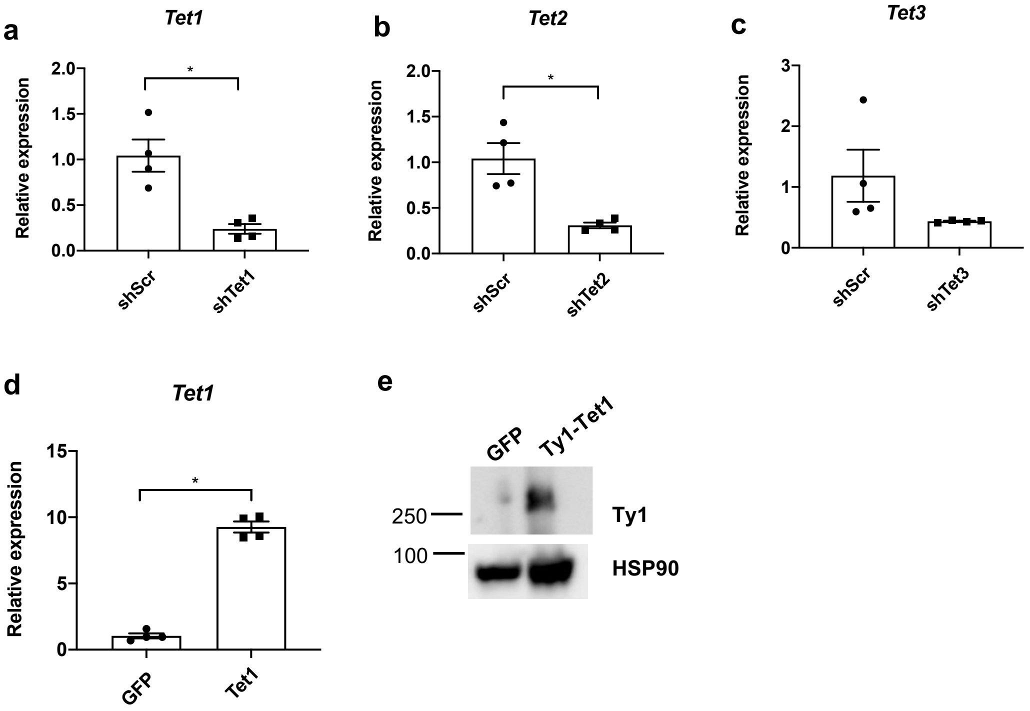

Supplementary Figure 2. Knockdown and overexpression efficiency of *Tet1* loss- and gain-of-function models *in vitro*. a-c Hairpins against Tet1, 2, and 3 (versus scrambled shRNA; shScr) were delivered to mature beige adipocytes (Day 6) via lentiviral transduction and the knockdown efficiency was evaluated by qPCR (n = 4 per group. Data are expressed as means ± SEM. *denotes $p < 0.05$, determined by two-tailed student's *t*-test). **d** Ty1-Tet1 or GFP was lentivirally transduced into mature beige adipocytes and the level of overexpression was evaluated by qPCR (n = 4 per group. Data are expressed as means ± SEM. *denotes $p < 0.05$, determined by two-tailed student's *t*-test) and **e** western blotting of Ty1 tag. Source data are provided as a source data file.

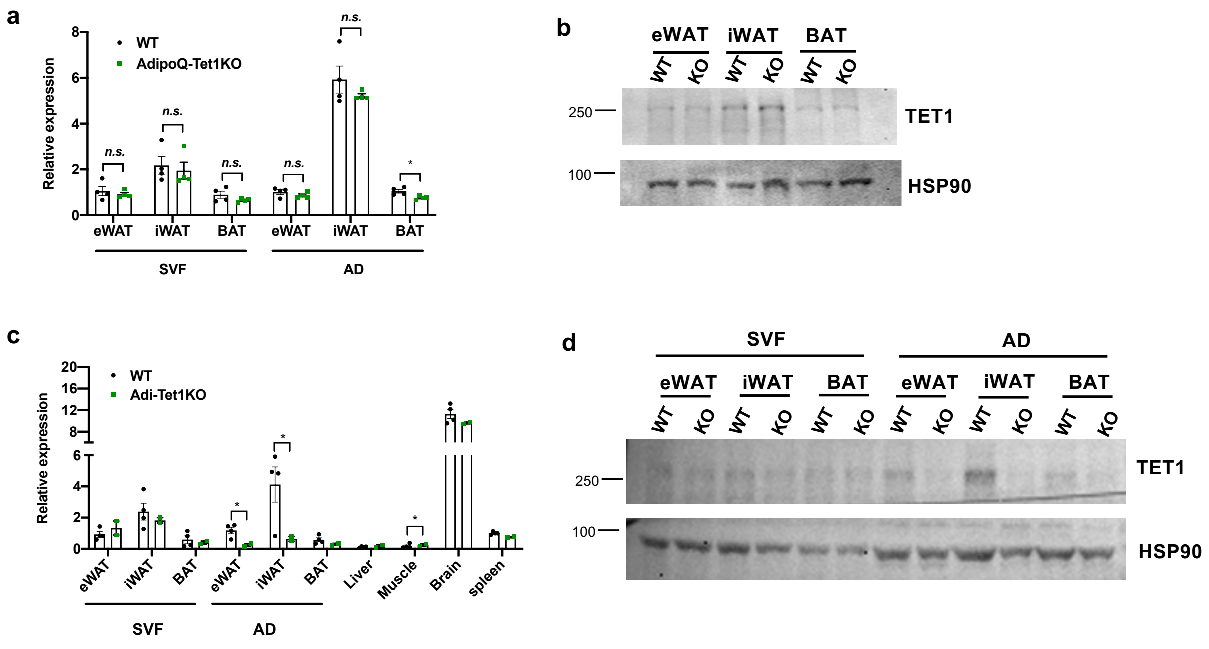

Supplementary Figure 3. Knockdown efficiency of adipose-specific *Tet1* KO models.
a Adipocyte-specific knockdown of Tet1 was examined in 8 wk-old Adipoq-Tet1 KO and WT mice by qPCR in stromal vascular fraction (SVF) vs. adipocyte fraction (AD) (n = 4 per group. Data are expressed as means ± SEM. *denotes $p < 0.05$, determined by two-tailed student's *t*-test). **b** Tet1 KD efficiency was tested by western blotting using protein lysates from eWAT, iWAT, and BAT tissues from Adipoq-Tet1 KO and wild type mice. **c, d** Tissue-specific knockdown of *Tet1* was examined in 8 wk-old Adi-Tet1 KO and WT mice by qPCR and western in stromal vascular fraction vs. adipocyte fraction and in multiple tissues (**c**: n = 3 per group. Data are expressed as means ± SEM. *denotes $p < 0.05$, determined by two-tailed student's *t*-test).

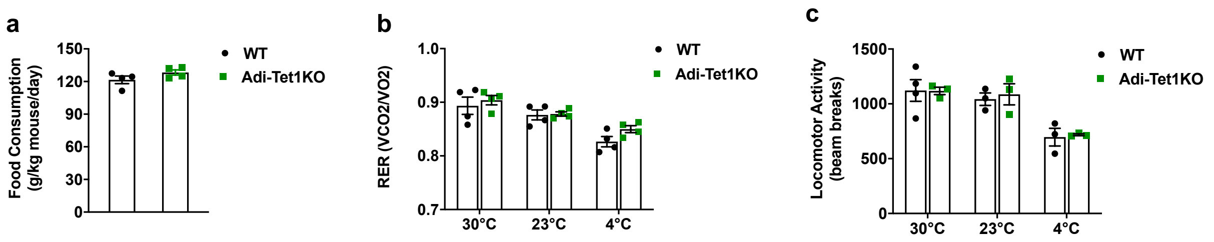

Supplementary Figure 4. Parameters from indirect calorimetry on chow. a Shown is the averaged production rates of food consumption, **b** the RER, and **c** locomotor activity of male Adi-*Tet1*-KO and WT littermates on chow (n = 4 per group. Data are expressed as means ± SEM. *denotes $p < 0.05$, determined by two-tailed student's *t*-test). (n=4 WT n=3 KO. Data are expressed as means ± SEM. *denotes $p < 0.05$, determined by two-tailed student's *t*-test). Source data are provided as a source data file.

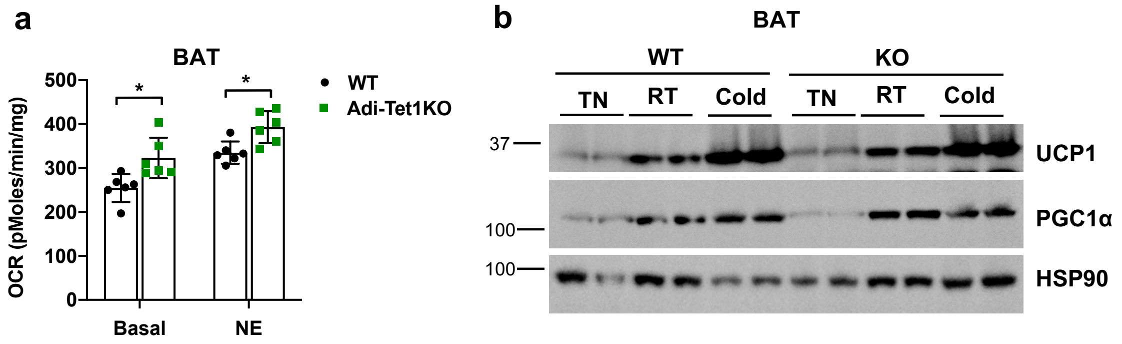

Supplementary Figure 5. Thermogenesis in the KO BAT. **a** Oxygen consumption rate of WT and KO BAT was measured with and without stimulation of NE (n = 6 per group. Data are expressed as means ± SEM. *denotes $p < 0.05$, determined by two-tailed student's *t*-test and two-way ANOVA followed by Bonferroni post-hoc testing). **b** Immunoblot of UCP1 and PGC1α from BAT from WT and KO mice at RT or housed at TN or cold for 7 days. Source data are provided as a source data file.

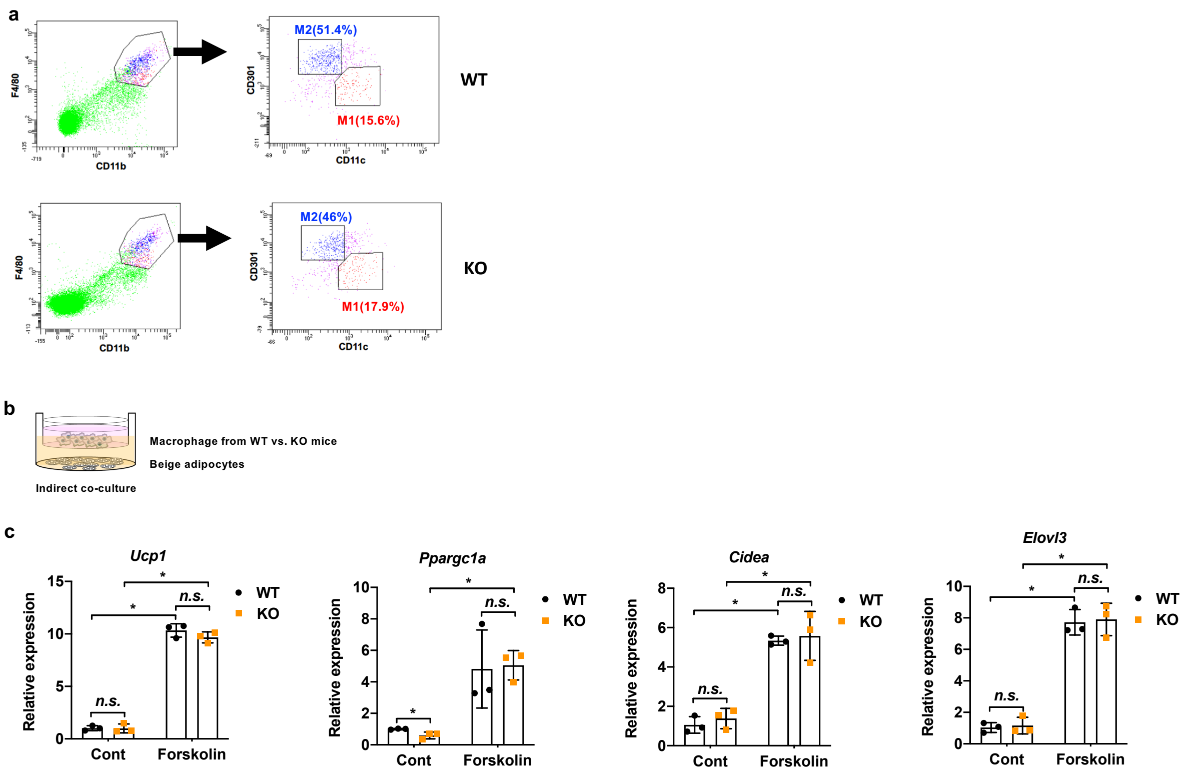

Supplementary Figure 6. Thermogenic gene expression is not affected by co-culture with macrophages from Adi-Tet1 KO mice. a Stromal vascular fractions (SVFs) of iWAT from WT (top) and Adi-KO (bottom) mice were stained with CD11b, F4/80, CD11c, and CD301. Scatterplots showing gates for M1 ($F4/80^{high}Cd11b^{high}CD11c^{+}CD301^{-}$) and M2 ($F4/80^{high}Cd11b^{high}CD11c^{-}CD301^{+}$) adipose tissue macrophages. **b, c** Primary macrophages were isolated from WT and Adi-Tet1 KO mice and co-cultured with mature wild type beige adipocytes for 24hr. The expression of a set of key thermogenic genes was measured by qPCR (n = 3 per group. Data are expressed as means ± SEM. *denotes $p < 0.05$, determined by two-tailed student's *t*-test and two-way followed by Bonferroni post-hoc testing). Source data are provided as a source data file.

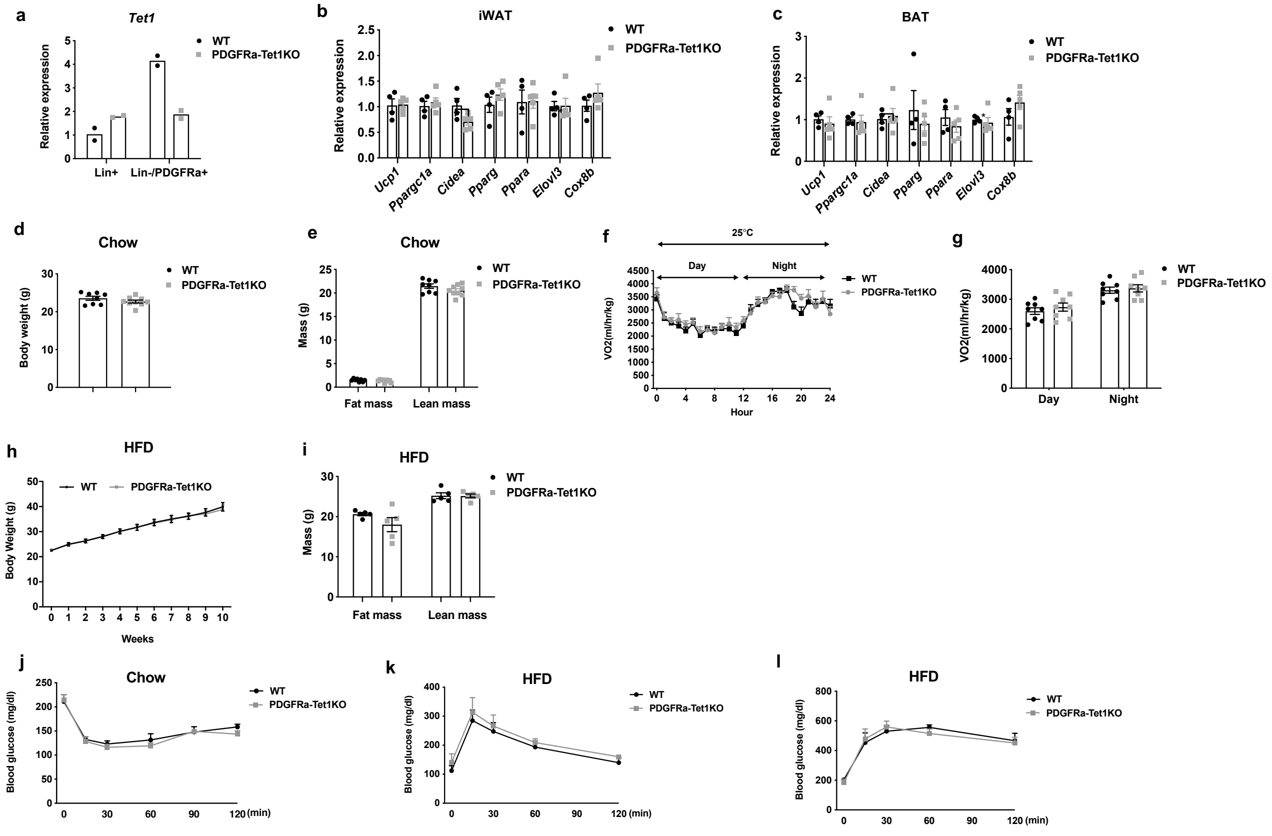

Supplementary Figure 7. PDGFRa-Tet1KO mice do not display any remarkable changes in thermogenesis, energy balance, and whole-body metabolism. a Tet1 expression was evaluated in Lin+ and Lin-/PDGFRa+ population from WT and PDGFRa-Tet1KO mice on chow (n=2 per group. Data are expressed as means). **b, c** Key adipocyte thermogenic gene transcripts were measured by qPCR in iWAT and BAT from chow-fed WT and PDGFRa-Tet1KO mice administered with CL316,243 for 5 consecutive days (n=4 WT n=5 KO. Data are expressed as means ± SEM. *denotes $p < 0.05$, determined by two-tailed student's *t*-test). **d** Body weight and **e** body composition from the eight-week old WT and PDGFRa-Tet1KO mice on chow diet (n=8 WT n=9 KO. Data are expressed as means ± SEM. *denotes $p < 0.05$, determined by two-tailed student's *t*-test). **f, g** Shown is the whole-body oxygen consumption rate (VO2, **f**) and averaged VO2 **g** from WT and PDGFRa-Tet1KO mice on chow diet at RT (n=8 per group. Data are expressed as means ± SEM. *denotes $p < 0.05$, determined by two-tailed student's *t*-test and two-way ANOVA followed by Bonferroni post-hoc testing). **h** Body weight and **i** body composition of WT and PDGFRa-Tet1KO mice on a high-fat diet (**h**: n=6 per group, **i**: n=5 per group. Data are expressed as means ± SEM. *denotes $p < 0.05$, determined by two-tailed student's *t*-test). **j** Insulin tolerance test from WT and PDGFRa-Tet1KO mice on chow (j: n=5 per group, Data are expressed as means ± SEM. *denotes $p < 0.05$, determined by two-tailed student's *t*-test). **k, l** Glucose tolerance test **k** and glucose tolerance test **l** on HFD, respectively (**k**: n=3 WT n=2 KO, and **l**: n=5 per group. Data are expressed as means ± SEM. *denotes $p < 0.05$, determined by two-tailed student's *t*-test). Source data are provided as a source data file.

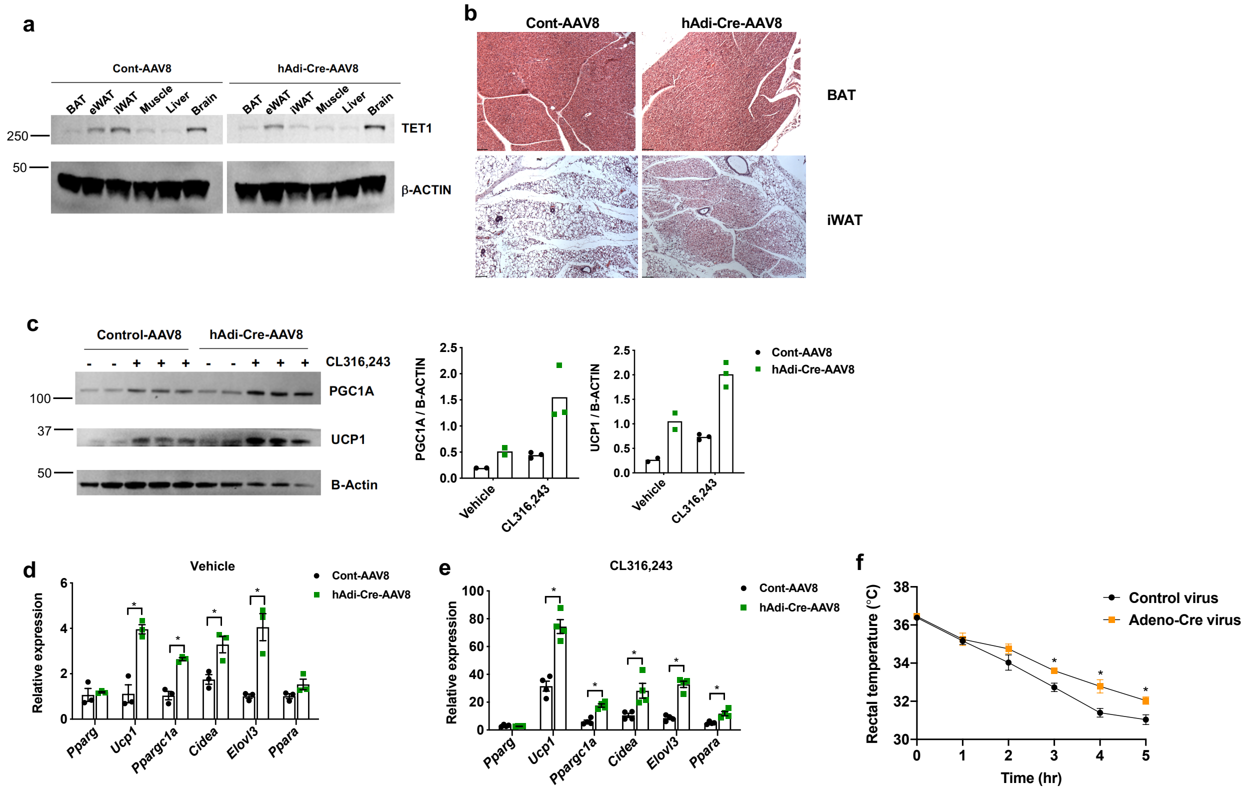

Supplementary Figure 8. Inguinal-selective Tet1 knock-down achieved AAV8-hAdiponectin-Cre leads to improved cold tolerance and increase in the key thermogenic genes. **a** Chow-fed Tet1 f/f mice were injected with AAV8-Vector control or AAV8-hAdi-Cre virus (1 × 10^{12} GC) via tail vein injection. Two weeks later, Tet1 knock-down efficiency was measured by western in various tissues. **b** H&E staining of iWAT and BAT from the AAV8-treated mice that were administered with CL316,243 for 5 consecutive days. **c-e** Key adipocyte thermogenic gene transcripts were measured by western **c** and qPCR **d, e** in iWAT from **b** (**d, e**: n=3 per group. Data are expressed as means ± SEM. *denotes $p < 0.05$, determined by two-tailed student's *t*-test). **f** Rectal core body temperatures of these virus treated mice under cold conditions at indicated time points (n=6 per group. Data are expressed as means ± SEM. *denotes $p < 0.05$, determined by two-tailed student's *t*-test). Source data are provided as a source data file.

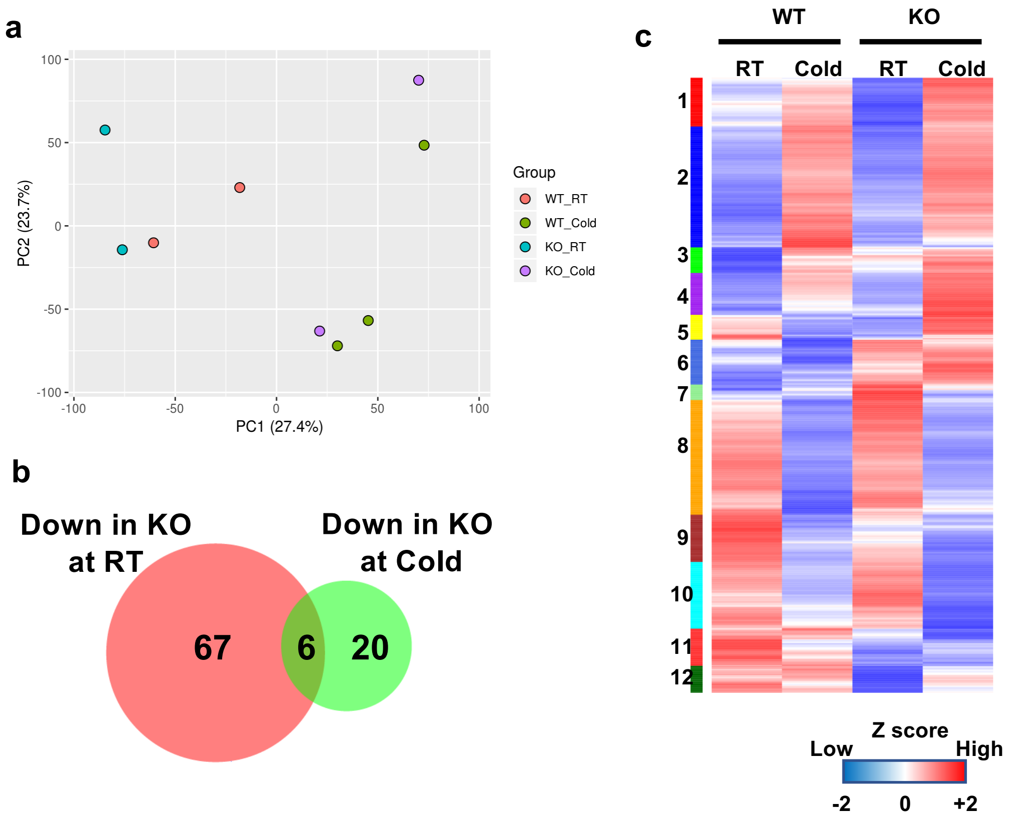

Supplementary Figure 9. RNA-Seq analysis from WT and KO adipocytes. a Principal component analysis of transcriptome of WT and KO adipocytes under RT and cold conditions. Each dot indicates an individual biological replicate (n=2 per group for RT, n=3 WT n=2 KO for Cold). **b** Venn diagram showing downregulated genes in KO under RT and cold conditions. **c** Hierarchical clustering and heatmap of transcriptional profiles for differentially regulated genes. The color scale shows z-scored FPKM representing the mRNA level of each gene in blue (low expression)-white-red (high expression) scheme. Complete gene lists are available in **Supplementary Table 2**.

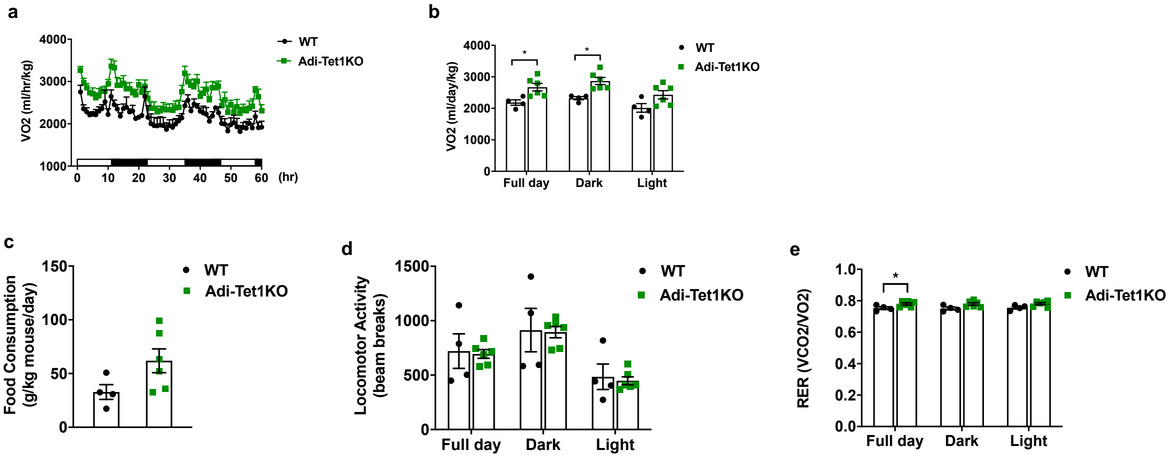

Supplementary Figure 10. Parameters from indirect calorimetry on HFD. a, b Shown is the whole-body oxygen consumption rate (VO2, **a**) and averaged VO2 **b** production rate measured on male *Tet1*-KO and WT littermates on HFD (n= 4 WT, n= 6 KO. Data are expressed as means ± SEM. *denotes $p < 0.05$, determined by two-tailed student's *t*-test and two-way ANOVA followed by Bonferroni post-hoc testing). **c-e** The averaged production rates of **c** food consumption **d** locomotor activity, and **e** the RER during the full day, dark, and light cycle measurements on male *Tet1*-KO and WT littermates on HFD (n= 4 WT, n= 6 KO. Data are expressed as means ± SEM. *denotes $p < 0.05$, determined by two-tailed student's *t*-test). Source data are provided as a source data file.

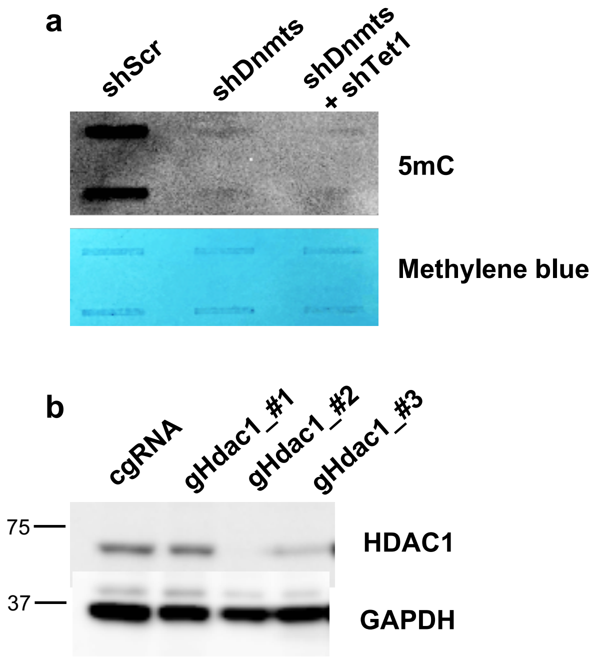

Supplementary Figure 11. Experimental controls. a Dot blot showing the global 5mC level in the cells knocked down for all three Dnmts, and Tet1, or scramble control. **b** Three individual distinct gRNAs against HDAC1 were tested in beige adipocytes and the knockdown efficiency was tested by western blotting. The #2 gHDAC1 was used in **Figs. 7i and j**. Source data are provided as a source data file.

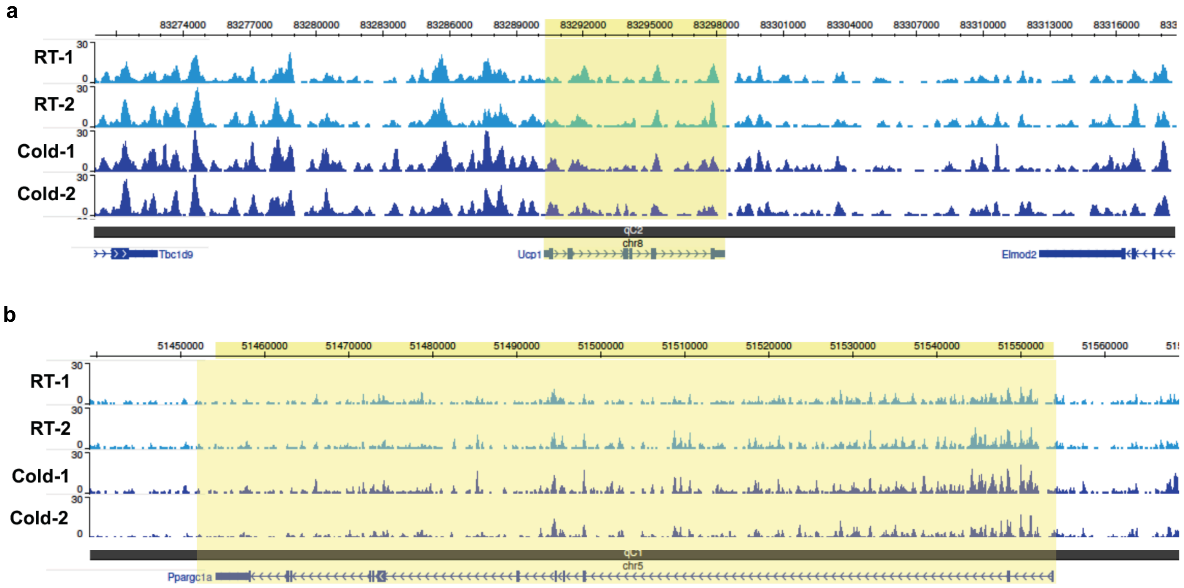

Supplementary Figure 12. 5hmC profile at *Ucp1* and *Ppargc1a* of iWAT from mice at RT or exposed to cold. a, b 5hmC-Seal-Seq was performed (see details in Materials and Method section) on genomic DNA isolated from the iWAT of C57BL/6J mice kept at RT or exposed to cold for 7 days. Shown are the 5hmC tracks at *Ucp1* **a** and *Ppargc1a* **b**, highlighted in yellow, at RT and Cold.

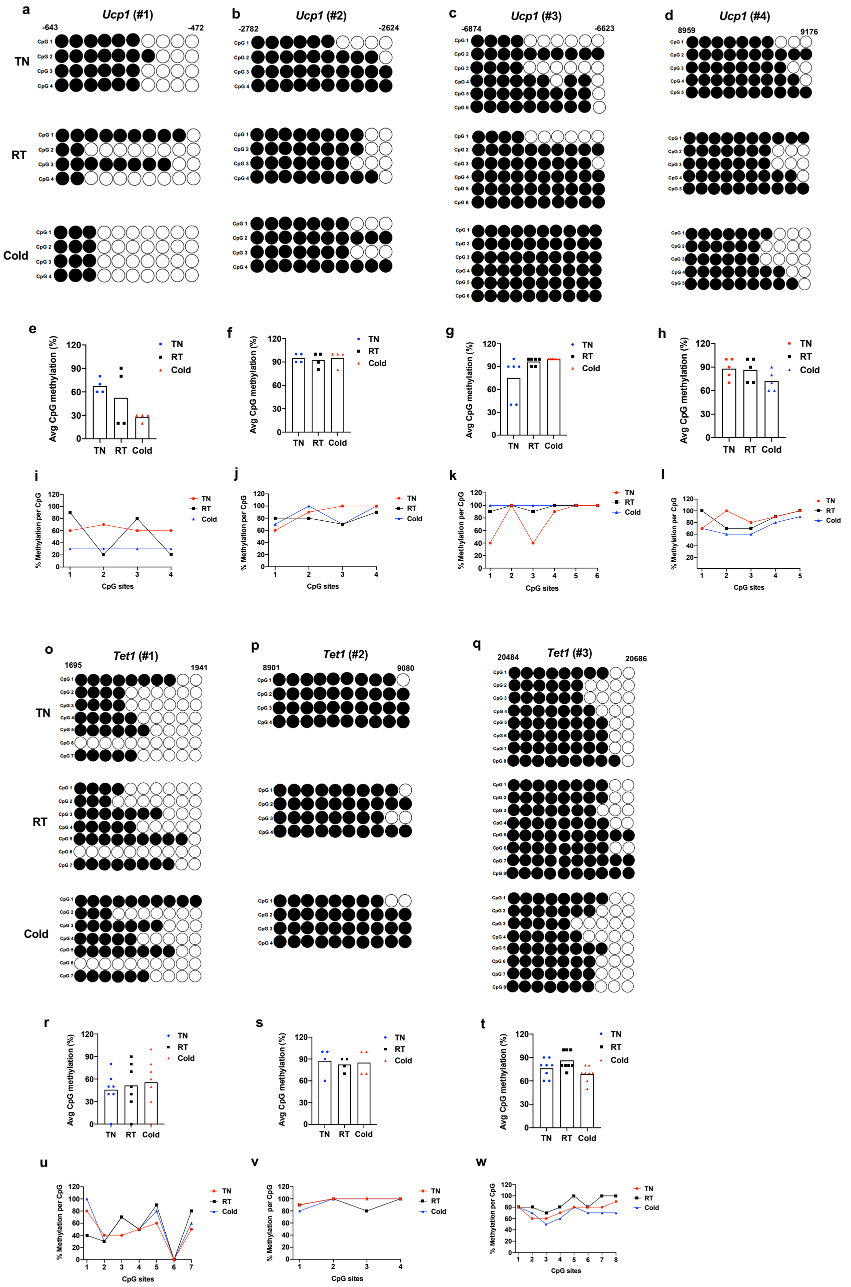

Supplementary Figure 13. Bisulfite sequencing analysis and quantification of 5mC. a-d, o-q Methylation status of individual CpGs at *Ucp1* and *Tet1* regulatory regions were measured by bisulfite sequencing using genomic DNAs isolated from the iWAT of C57BL/6J mice under different temperature conditions (n=1 per group). The numbers indicate the distance from transcription start site of *Ucp1* and *Tet1*. **e-h, r-t** Average methylation levels at the tested regions from a single representative experiment. **i-l, u-w** Methylation levels at individual CpG site from a single representative experiment. Source data are provided as a source data file.

Supplementary Table 1

Gene list in Figure 4I

RT. WT vs Cold. Up	Cold. WT vs RT. Up	Commonly up in KO at RT and Cold
Abi3	A330041J22Rik	Abi3
Acaa2	A530050N04Rik	Acaa2
Acot11	Abi3	Acot11
Agtr1a	Acaa2	Cidea
Atp1b1	Acacb	Clstn3
Atp7b	Acot11	Cox7a1
Bdnf	Acss1	Cox8b
Cidea	Adcy3	Cpt1b
Clic5	Ankrd9	Dio2
Clstn3	Apold1	Fabp3
Coq10b	Aspg	Fam151a
Cox7a1	Atp2a1	Gk
Cox8b	Calm4	Gm44502
Cpt1b	Cend1	Otop1
Csn1s1	Cidea	Pank1
Csn2	Ckm	Phospho1
Dbp	Cldn1	Ppara
Dcpp3	Clstn3	Ppargc1a
Defb15	Coch	Ppp1r3b
Dio2	Coq8a	Slc27a2
Fabp3	Cox7a1	Ucp1
Fam151a	Cox8b	
Fcgbp	Cpt1b	
Gk	Crct1	
Gm44502	Ctnnbl1	
Gm4841	Cyp2g1	
Gm5148	Dagla	

Gys2
Hsd11b1
Muc15
Nr1d2
Nr4a2
Nr4a3
Otop1
Pank1
Per2
Per3
Phospho1
Pnldc1
Ppara
Ppargc1a
Ppp1r3b
Ppp1r3d
Sbp
Slc27a2
Tef
Tnfsf11
Ucp1
Vldlr
Wnk2
Wnk4

Defb6
Dhrs9
Dio2
Dmkn
Dpep1
Elovl3
Elovl6
Fa2h
Fabp3
Fam151a
Fam69b
Gk
Gm44502
Gm94
Gm9899
Gmpr
Gnao1
Gpd2
Hamp2
Hc
Inmt
Kcnh1
Kcnk3
Krt10
Krtdap
Lce1m
Ldhb
Letmd1
Lor
Lrrc52
Me1
Mmp11
Mrgprg

Mtfp1
Otop1
Pamr1
Pank1
Pdk4
Perm1
Phospho1
Pim1
Ppara
Ppargc1a
Ppif
Ppp1r3b
Psapl1
Pvalb
Pygl
Rorc
Serpina3b
Serpina3j
Slc25a34
Slc27a2
Slc2a5
Slc4a4
Sorcs2
Tnni2
Tnnt3
Ucp1
Ucp3

Supplementary Table 1

Gene list in Supplementary Figure 9b

RT. WT vs Cold down	Cold. WT vs KO down	Commonly down in KO at RT and Cold
Akr1c14	3830417A13Rik	Aoc1
Alox5ap	Agt	C4b
Aoc1	Aldh1l2	Chrdl1
Aqp3	Aoc1	Faim2
Aqp5	Bmper	Padi4
Areg	C2	Vnn1
Arsi	C4b	
Bst1	Cfb	
C4b	Chrdl1	
Ccl6	Creb3l1	
Ccl8	Dclk1	
Ccl9	Faim2	
Cd209f	Fam83e	
Cd209g	Ffar2	
Cfp	Fgf10	
Chrdl1	Irs3	
Chsy3	Mcam	
Cldn11	Padi4	
Clec3b	Prr32	
Cystm1	Prtn3	
Dab2	Serpinh1	
Dscam	Sncg	
Faim2	Spon1	
Fbln1	Timp1	
Fcgr1	Ttyh2	
Fcgr2b	Vnn1	

Fcrls
Gas6
Gm13031
Gm14005
Gng12
Gsr
Hif1a
Il33
Irf4
Itgam
Krt4
Ldb3
Lgi2
Mafb
Matn4
Mcpt4
Mgl2
Mmp9
Mrc1
Ms4a6d
Muc13
Nfam1
Ngfr
Ninj1
Npl
Padi2
Padi4
Pinc
Ptpn5
Sfrp4
Siglec1
Slco2a1
Slpi

Spi1
Sprr1a
Stc2
Syn2
Tcf21
Tgm2
Thbs2
Thsd4
Tmed3
Tmem176a
Tmem86a
Vnn1
Wfdc17
Wfdc3

Supplementary Table 1

Gene list in Supplementary Figure 9c

	WT.RT_vs_Cold				KO.RT_vs_Cold				RT.WT_vs_KO				Cold.WT_vs_KO				
Geneid	logFC.WT.RT_vs_Cold	logCPM.WT.RT_vs_Cold	PValue.WT.RT_vs_Cold	FDR.WT.RT_vs_Cold	logFC.KO.RT_vs_Cold	logCPM.KO.RT_vs_Cold	PValue.KO.RT_vs_Cold	FDR.KO.RT_vs_Cold	logFC.RT.WT_vs_KO	logCPM.RT.WT_vs_KO	PValue.RT.WT_vs_KO	FDR.RT.WT_vs_KO	logFC.Cold.WT_vs_KO	logCPM.Cold.WT_vs_KO	PValue.Cold.WT_vs_KO	FDR.Cold.WT_vs_KO	Cluster
Apod	-0.108629498	6.59238302	0.582655657	0.954541726	1.035764252	6.59238302	3.39E-06	0.000461389	-0.543619559	6.59238302	1.41E-02	0.413271079	0.600774147	6.59238302	2.48E-03	0.171830909	red
Rcan2	-0.042990323	3.599820475	0.851483073	0.997680032	0.977146414	3.599820475	0.000219668	0.011684579	-0.462551221	3.599820475	0.080220352	0.836876692	0.557584941	3.599820475	0.017672149	0.540450678	red
St3gal5	0.021541019	3.572153343	0.934465195	1	0.931215689	3.572153343	0.000125962	0.007620975	-0.419007713	3.572153343	0.083845486	0.845870221	0.490666349	3.572153343	2.33E-02	0.632583692	red
Ace	0.051509485	7.301925217	0.800094008	0.989314717	0.710066582	7.301925217	0.000995707	0.035376901	-0.316017559	7.301925217	0.141616318	0.992645357	0.3425395	7.301925217	7.67E-02	1	red
Fam25c	0.165203378	4.344488493	0.64645348	0.97151377	1.660163128	4.344488493	1.93E-05	0.001691751	-0.735959739	4.344488493	0.054320648	0.732097757	0.758999891	4.344488493	0.024906296	0.659262104	red
Zfp750	0.174427292	2.662104677	0.564192913	0.947517612	1.028637536	2.662104677	0.001564936	0.047858975	-0.41124793	2.662104677	2.08E-01	1	0.442961031	2.662104677	1.24E-01	1	red
Dapl1	0.604541	-1.188581036	0.460091407	0.914765341	3.259240396	-1.188581036	0.000405527	0.018211169	-1.388311045	-1.188581036	0.160763521	1	1.266503814	-1.188581036	0.072736542	1	red
Ntn4	0.078045357	4.717393443	0.698748702	0.976386163	0.798224403	4.717393443	0.00021161	0.011374868	-0.210104122	4.717393443	3.30E-01	1.00E+00	0.510074255	4.717393443	0.008193833	0.352360031	red
Id2	0.264001479	4.779896333	0.191991381	0.738408831	0.879868642	4.779896333	6.19E-05	0.004281366	-0.182868761	4.779896333	4.05E-01	1	0.432997702	4.779896333	0.027477913	0.68738954	red
Kcnn4	0.415523222	2.951743833	0.161208992	0.697705454	1.088543458	2.951743833	0.000676582	0.026787291	-0.172421197	2.951743833	5.91E-01	1	0.500595697	2.951743833	0.077522789	1	red
Reck	0.386068123	5.763016512	0.04337264	0.401411393	0.950150357	5.763016512	4.90E-06	0.000600532	-0.164504872	5.763016512	0.427403852	1	0.39957697	5.763016512	0.031484941	0.731958842	red
Rassf4	0.293676712	5.34100582	0.116308801	0.619025547	0.832147256	5.34100582	4.29E-05	0.00316872	-0.20860602	5.34100582	3.05E-01	1	0.329864166	5.34100582	0.070050714	1	red
Eps8	0.233819876	6.453091876	0.166387644	0.705746194	0.629167667	6.453091876	0.000613118	0.02500553	-0.189061366	6.453091876	3.03E-01	1	0.206286325	6.453091876	0.211571846	1	red
Hey1	0.424145713	4.959743294	0.014512186	0.209106904	0.86604804	4.959743294	4.36E-06	0.000557251	-0.2122628	4.959743294	0.260406491	1	0.229639108	4.959743294	0.17349847	1	red
Cldn10	0.560969044	4.45898688	0.01681925	0.231820347	1.079844511	4.45898688	2.25E-05	0.001912749	-0.226148505	4.45898688	0.374655738	1	0.292726139	4.45898688	0.195619443	1	red
Msx2	0.795494123	3.322347333	0.002717414	0.073176444	1.514905773	3.322347333	1.61E-07	3.81E-05	-0.33145887	3.322347333	2.50E-01	1	0.387950599	3.322347333	0.123870545	1	red
Cdk19	0.331489928	7.539643017	0.056436628	0.453570925	0.602806829	7.539643017	0.001395026	0.044110588	-0.128383003	7.539643017	0.495182843	1	0.142933841	7.539643017	0.398746596	1	red
Prom2	1.193112359	7.303735173	1.47E-05	0.001552812	2.171873275	7.303735173	6.22E-13	6.21E-10	-0.500618215	7.303735173	0.084863321	0.847346568	0.478142558	7.303735173	0.065645285	1	red
Adh7	0.918256486	2.140129622	0.008923557	0.157437674	1.631338468	2.140129622	1.87E-05	0.001663136	-0.429150333	2.140129622	2.62E-01	1	0.283928313	2.140129622	0.387808361	1	red
6030408B16Rik	1.816291462	0.415370821	0.000852512	0.031441109	3.826496932	0.415370821	1.14E-09	5.71E-07	-1.331113894	0.415370821	4.51E-02	0.681041299	0.679156217	0.415370821	0.151489999	1	red
Col28a1	0.433126744	3.806931822	0.082501167	0.537546063	0.924274756	3.806931822	0.00064078	0.025883069	-0.271557314	3.806931822	0.317622611	1	0.219589971	3.806931822	0.361461426	1	red
Def6	0.426175678	3.716150998	0.052602126	0.436819557	1.013264523	3.716150998	2.26E-05	0.001912749	-0.32078424	3.716150998	1.81E-01	1	0.266303865	3.716150998	0.21037123	1	red
Slc7a8	0.300898852	4.946685521	0.172238746	0.714266342	0.977572101	4.946685521	4.56E-05	0.003305395	-0.371589234	4.946685521	1.20E-01	0.946835239	0.305083801	4.946685521	0.153435069	1	red
Zfp777	0.190997658	4.561099036	0.275455756	0.817343958	0.61028473	4.561099036	0.001310515	0.042008616	-0.232122923	4.561099036	2.23E-01	1.00E+00	0.187163922	4.561099036	0.272400516	1	red
Drd1	0.644391869	0.948295321	0.154368707	0.685995882	2.588432391	0.948295321	4.13E-07	8.48E-05	-1.186402323	0.948295321	0.018815889	0.461913105	0.75765513	0.948295321	0.068401727	1	red
Arhgef10l	0.203943327	4.997584812	0.263686641	0.807177783	0.708945752	4.997584812	0.000343644	0.016225557	-0.252351038	4.997584812	0.202719654	1	0.252651149	4.997584812	0.154911985	1	red
Foxi1	0.472329241	2.584436715	0.217032234	0.763079875	2.14615444	2.584436715	5.37E-07	0.000105658	-0.999030216	2.584436715	1.76E-02	0.447803905	0.674797772	2.584436715	0.063289121	0.993852541	red
Runx1	0.297354695	6.731805583	0.188421799	0.734359705	1.017217784	6.731805583	3.64E-05	0.002749178	-0.45020953	6.731805583	0.065698844	0.780788307	0.269653544	6.731805583	0.219039196	1	red
Lcn2	0.256109881	7.737800922	0.165912742	0.705006144	0.946897534	7.737800922	2.68E-06	0.000382229	-0.407529938	7.737800922	4.20E-02	6.70E-01	0.283257699	7.737800922	0.116448545	1	red
Bmp4	0.269394298	2.300989767	0.373898938	0.883152198	1.071054834	2.300989767	0.000997317	0.035376901	-0.505448776	2.300989767	1.24E-01	0.956226206	0.296211848	2.300989767	0.303847132	1	red
Adamtsl1	0.158561813	4.953771025	0.490991704	0.929487183	0.889304942	4.953771025	0.000360471	0.016793163	-0.522752823	4.953771025	3.55E-02	0.628189489	0.207990509	4.953771025	0.349045927	1	red
Csn3	0.150930724	7.557973649	0.474281064	0.921496564	1.143601691	7.557973649	6.71E-07	0.000125045	-0.645664474	7.557973649	4.68E-03	0.219530831	0.34700653	7.557973649	0.090096759	1	red
Clic6	0.062991515	4.699448096	0.783681798	0.987952495	1.007920412	4.699448096	3.44E-05	0.00263835	-0.6588512	4.699448096	6.61E-03	0.267790873	0.286078107	4.699448096	0.186665469	1	red
1810010D01Rik	0.140318619	2.830844714	0.779992735	0.987750219	2.459296875	2.830844714	4.56E-06	0.000564114	-1.694211522	2.830844714	0.001268888	0.098522137	0.624776932	2.830844714	0.163499204	1	red
Papln	0.075070308	6.582228478	0.714856448	0.978725873	0.735656311	6.582228478	0.000836555	0.0312612	-0.583012877	6.582228478	0.008011331	0.297782883	0.077573286	6.582228478	0.690783319	1	red
Cd163	0.262167102	6.227056219	0.39414502	0.889563201	1.281352357	6.227056219	0.00013016	0.007740903	-0.830848634	6.227056219	0.012370117	0.382488401	0.1883369	6.227056219	0.520286402	1	red
Sulf1	0.169512068	5.081276831	0.30946396	0.841289301	0.658895397	5.081276831	2.84E-04	0.013934845	-0.439533582	5.081276831	1.54E-02	4.27E-01	0.049850018	5.081276831	0.757051817	1	red
Alox15	0.592229653	3.274833768	0.128121799	0.640425702	1.916626079	3.274833768	8.64E-06	0.000903978	-1.278047451	3.274833768	0.002652601	0.159124298	0.046354666	3.274833768	0.8905699	1	red
Fam3b	1.092836701	1.88675853	0.072798644	0.515060954	3.588994703	1.88675853	5.73E-07	0.000111216	-2.214301856	1.88675853	1.25E-03	0.09783071	0.281912096	1.88675853	0.60537579	1	red
Rnf144b	0.22200944	5.124236763	0.22451287	0.77038838	0.647364879	5.124236763	1.13E-03	3.83E-02	-0.363287952	5.124236763	6.75E-02	7.92E-01	0.062067618	5.124236763	0.725157337	1	red
Cysrt1	0.647293109	1.765860464	0.112801271	0.611858553	2.14546039	1.765860464	2.72E-06	0.000383913	-1.22878985	1.765860464	7.07E-03	0.279062846	0.269391153	1.765860464	0.481090548	1	red
Zmiz1	0.285949049	8.640694867	0.125873262	0.637525066	0.93472613	8.640694867	4.52E-06	0.000563716	-0.505722767	8.640694867	1.26E-02	0.383289531	0.143054329	8.640694867	0.431475949	1	red
Zfp143	0.214463042	3.827112486	0.31227211	0.844621698	0.758735935	3.827112486	0.000987034	0.035371258	-0.385700316	3.827112486	9.48E-02	0.884946568	0.158572689	3.827112486	0.440127118	1	red
Cyb561	0.244311653	5.535540306	0.107149844	0.599010487	0.794593564	5.535540306	1.56E-06	0.000240168	-0.368533205	5.535540306	2.57E-02	5.39E-01	0.181748691	5.535540306	0.220696391	1	red
Scara5	0.497965001	6.411587518	0.068189138	0.500268448	1.470035916	6.411587518	9.08E-07	0.000154731	-0.699680935	6.411587518	1.78E-02	0.448304783	0.27239008	6.411587518	0.299919881	1	red
Cldn4	0.378860817	7.154624181	0.090540117	0.558177622	0.947715297	7.154624181	9.94E-05	0.0063217	-0.422580263	7.154624181	0.080761165	0.836876692	0.146274221	7.154624181	0.498668309	1	red
Stc2	1.083863905	7.31740887	2.36E-05	0.00208639	2.575575524	7.31740887	2.70E-19	1.89E-15	-1.132551069	7.31740887	3.72E-05	0.008765327	0.359160796	7.31740887	0.139665141	1	red
Ctr9	0.415157815	7.113055703	0.040457518	0.386489594	0.989083411	7.113055703	7.39E-06	0.000813143	-0.443338818	7.113055703	0.043125531	0.67446002	0.13058679	7.113055703	0.505453194	1	red
Stat5a	0.368987842	7.602951136	0.081888816	0.536532527	0.89801574	7.602951136	9.92E-05	0.0063217	-0.436984984	7.602951136	5.68E-02	0.746059679	0.092042933	7.602951136	0.651852025	1	red
Enho	0.911628034	4.445085927	8.97E-05	0.006056455	1.80937368	4.445085927	1.42E-12	1.32E-09	-0.724677859	4.445085927	0.00406991	0.203874789	0.173068205	4.445085927	0.434693387	1	red
Cpt1a	0.390321415	7.124552153	0.051655405	0.434844135	0.695833704	7.124552153	0.001383526	0.044045928	-0.251644837	7.124552153	0.246001848	1	0.05386743	7.124552153	0.777997688	1	red
Qtrt1	0.618732067	3.624706106	0.008030723	0.146915695	1.031445015	3.624706106	4.76E-05	0.003425797	-0.3209774	3.624706106	0.206353254	1	0.091735002	3.624706106	0.679981263	1	red
Pxylp1	0.595353778	4.803232813	0.003451559	0.085834491	1.103664354	4.803232813	6.18E-07	0.000116808	-0.377193333	4.803232813	8.73E-02	0.851791999	0.131117062	4.803232813	0.503285796	1	red
Adamts15	0.551168963	5.462432794	0.009344882	0.161096467	1.057607686	5.462432794	4.46E-06	0.000561248	-0.378589997	5.462432794	9.84E-02	0.891625163	0.127848636	5.462432794	0.53130487	1	red
Prmt1	0.303739022	6.160411664	0.048404967	0.424142837	0.594698156	6.160411664	0.000378825	0.017358896	-0.257023175	6.160411664	1.24E-01	0.956226206	0.033935958	6.160411664	0.818248367	1	red
H2-M9	0.864382082	2.522791244	0.00703368	0.136911847	1.59636803	2.522791244	5.92E-06	0.000676573	-0.7134494	2.522791244	0.042267026	0.670515276	0.018539024	2.522791244	0.94486982	1	red
Vtcn1	0.939446473	5.136658977	0.002186164	0.062101279	1.875998567	5.136658977	2.44E-08	7.75E-06	-1.054158456	5.136658977	1.42E-03	0.10532897	-0.117605132	5.136658977	0.702684197	1	red
Nhp2	0.204413861	4.983199247	2.12E-01	0.758098043	0.639607698	4.983199247	0.000338	0.016122503	-0.478958572	4.983199247	7.27E-03	0.282156373	-0.043764286	4.983199247	0.794104952	1	red
Akr1c14	0.347356159	4.373696618	0.099717277	0.584339063	1.094363244	4.373696618	2.30E-06	0.000334893	-0.847711089	4.373696618	2.46E-04	0.031790079	-0.100702473	4.373696618	0.636472807	1	red
Myh14	0.210179218	6.118380968	0.213024496	0.75907387	0.621114327	6.118380968	0.000720984	0.028068174	-0.47809807	6.118380968	9.17E-03	0.322895246	-0.067162744	6.118380968	0.69372821	1	red
Aqp5	0.769041214	7.80088837	1.72E-02	0.2336509	1.896364912	7.80088837	9.60E-08	2.40E-05	-1.363748135	7.80088837	9.85E-05	0.016781558	-0.236424115	7.80088837	0.462375081	1	red
Cd200	0.386305358	7.750834621	3.51E-02	0.355195794	0.65974994	7.750834621	0.000914064	0.033796168	-0.427683661	7.750834621	0.03123725	0.597225455	-0.154239017	7.750834621	0.399910344	1	red
Syn2	1.777345693	7.014831259	5.10E-05	3.83E-03	2.584730993	7.014831259	5.56E-08	1.55E-05	-1.677943568	7.014831259	2.76E-04	0.034401024	-0.870557036	7.014831259	0.03997795	0.812110221	red
Fam129b	0.622058418	7.489032598	5.95E-04	0.024809744	0.679350763	7.489032598	0.000511352	0.021656544	-0.256302859	7.489032598	1.89E-01	1	-0.199010499	7.489032598	0.268153507	1	blue
Ngp	2.112559308	-0.76626641	0.00020641	0.010942234	3.158218114	-0.76626641	9.54E-06	0.000980484	-1.573976912	-0.76626641	0.050964427	0.712278826	-0.528036514	-0.76626641	0.323751828	1	blue
Ctla2a	0.727397471	5.655025448	8.07E-05	0.005585307	0.790203008	5.655025448	7.24E-05	0.004849568	-0.23981123	5.655025448	0.227967138	1	-0.177005724	5.655025448	3.32E-01	1	blue
Glt28d2	0.692891243	3.900078996	0.00098505	0.034677726	0.784135807	3.900078996	5.69E-04	0.023652779	-0.237171127	3.900078996	0.299738622	1	-0.145926711	3.900078996	4.85E-01	1	blue
Mthfd1l	1.239884453	2.780500245	1.46E-05	0.001552812	1.309023667	2.780500245	2.19E-05	0.001879395	-0.361212668	2.780500245	0.245306857	1	-0.292074002	2.780500245	0.292159216	1	blue
Parvb	0.783638711	5.130284373	0.000201603	0.010836924	0.62500825	5.130284373	0.005797158	0.111907568	-0.087119733	5.130284373	0.702172992	1	-0.245750386	5.130284373	0.237955794	1	blue
Man2a1	0.740720719	7.432131169	2.49E-05	0.002117227	0.689815523	7.432131169	0.000262539	0.013246362	-0.189379112	7.432131169	0.314868398	1	-0.240284316	7.432131169	1.66E-01	1	blue
Sgsm1	0.752582301	3.199394697	0.003399491	0.085298535	0.937916814	3.199394697	0.000751222	0.029002995	-0.342960037	3.199394697	0.219765638	1	-0.157625324	3.199394697	5.39E-01	1	blue
Gpr160	0.670086627	4.491463897	0.003094931	0.079144821	0.861691145	4.491463897	0.000441505	0.019349248	-0.317271206	4.491463897	0.195989662	1	-0.125666636	4.491463897	0.578822714	1	blue
Lrp5	0.465327383	7.87439146	0.007551053	0.141465839	0.607281927	7.87439146	0.001284106	0.041639587	-0.224007539	7.87439146	0.233820081	1	-0.082052995	7.87439146	0.63865926	1	blue
Lad1	0.525797576	5.404230021	0.009133695	0.159147836	0.762758298	5.404230021	4.83E-04	0.020649956	-0.329983591	5.404230021	0.130368311	0.975562438	-0.093022785	5.404230021	6.47E-01	1	blue
Cdk18	0.760833455	5.588956451	0.010314218	0.173051033	1.181349819	5.588956451	0.000229181	0.011978651	-0.488141812	5.588956451	0.124432423	0.956226206	-0.067625337	5.588956451	0.825756789	1	blue
Sla	0.695242564	3.24274055	0.010135419	0.171078031	1.002904226	3.24274055	0.000629511	0.025509877	-0.379403897	3.24274055	0.198232799	1	-0.071742101	3.24274055	0.797370332	1	blue
C77080	0.46997306	6.137437638	0.004363467	0.099809843	0.669079772	6.137437638	1.83E-04	0.010003083	-0.205373846	6.137437638	0.250252114	1	-0.006267194	6.137437638	9.76E-01	1	blue
Cd9	0.558244469	8.230203287	0.001686742	0.051136454	0.781493978	8.230203287	4.88E-05	0.003482824	-0.235378252	8.230203287	0.219065602	1	-0.012128758	8.230203287	0.951187036	1	blue
Cyp4b1	0.6961554	6.496385744	0.001389877	0.044992576	0.869747299	6.496385744	2.15E-04	0.011512853	-0.253854153	6.496385744	0.277849216	1	-0.080262303	6.496385744	7.14E-01	1	blue
Ms4a4a	1.268468784	3.425803973	0.000137601	0.00870187	1.626934314	3.425803973	5.91E-06	0.000676573	-0.499508754	3.425803973	0.162601511	1	-0.141043351	3.425803973	0.668687907	1	blue
Id1	0.813859355	5.261985505	1.79E-05	0.001750728	0.910820895	5.261985505	8.45E-06	0.00090111	-0.188354032	5.261985505	0.35710534	1	-0.091392756	5.261985505	0.627877387	1	blue
Myrip	1.024611517	3.699815663	5.13E-05	0.003831278	1.138243604	3.699815663	2.87E-05	0.002304695	-0.252005643	3.699815663	0.356626234	1	-0.138374379	3.699815663	0.579034025	1	blue
Tinagl1	1.038768848	8.021457301	1.24E-06	0.000217262	1.066640464	8.021457301	3.28E-06	0.000454905	-0.216005853	8.021457301	0.341522047	1	-0.188134279	8.021457301	3.70E-01	1	blue
Plekhh1	0.755339538	5.817131215	7.32E-06	0.000947368	0.719575037	5.817131215	7.25E-05	0.004849568	-0.080170445	5.817131215	0.659600358	1	-0.115935163	5.817131215	4.87E-01	1	blue
Fam107a	1.709343976	4.194558541	2.25E-09	9.54E-07	1.785381605	4.194558541	4.25E-09	1.80E-06	-0.239937537	4.194558541	0.426095564	1	-0.163901722	4.194558541	0.549361441	1	blue
Gpihbp1	0.795007848	7.542514277	0.000170251	0.010039767	0.854056766	7.542514277	1.72E-04	0.009565959	-0.107261057	7.542514277	0.635437462	1	-0.04821222	7.542514277	0.822578103	1	blue
Pkig	0.551312279	5.244698588	0.000519693	0.02225488	0.682681483	5.244698588	7.24E-05	0.004849568	-0.127344121	5.244698588	0.460828126	1	0.004024819	5.244698588	0.975034494	1	blue
Foxa1	0.996571344	6.985665223	4.53E-05	0.003457768	1.176184119	6.985665223	7.34E-06	0.000813143	-0.190789579	6.985665223	0.462302718	1	-0.011176952	6.985665223	0.971230211	1	blue
Epn2	0.506365994	6.414392354	0.001333669	0.044095063	0.618156512	6.414392354	0.000296527	0.014439958	-0.087103764	6.414392354	0.610413156	1	0.024686621	6.414392354	0.868502147	1	blue
Mthfd2	0.873759819	3.335054493	0.001240012	0.041659638	1.055732843	3.335054493	0.000288689	0.014107398	-0.141425276	3.335054493	0.632425329	1	0.040545772	3.335054493	0.869482856	1	blue
Lpin3	0.694504091	4.242109572	0.000226705	0.011734911	0.77566468	4.242109572	0.00013592	0.007981601	-0.079872055	4.242109572	0.69844175	1	0.001287697	4.242109572	0.990251483	1	blue
Hspb7	1.786455381	1.426913931	5.47E-05	0.004069838	2.055399164	1.426913931	1.62E-05	0.001485077	-0.320225525	1.426913931	0.523648078	1	-0.051294645	1.426913931	0.921353627	1	blue
Btla	1.439206133	2.12675153	0.001112007	0.038091704	1.624285915	2.12675153	0.000553683	0.023238073	-0.192909663	2.12675153	0.687319854	1	-0.00783751	2.12675153	1	1	blue
Jaml	1.696809789	1.398078298	0.000209527	0.011008846	1.874235327	1.398078298	1.14E-04	0.007017402	-0.078097681	1.398078298	0.878460532	1	0.099305974	1.398078298	0.801861759	1	blue
Fgfr3	0.73842259	4.263413093	0.000901689	0.032563333	0.782537733	4.263413093	1.08E-03	0.037238092	-0.028451279	4.263413093	0.908111031	1	0.01566282	4.263413093	0.935891246	1	blue
Rbp7	1.05609521	5.983771762	5.35E-06	0.000740071	1.118141491	5.983771762	6.77E-06	0.000763489	-0.041659931	5.983771762	0.867434166	1	0.020385879	5.983771762	0.919727644	1	blue
Slc38a1	0.925779927	5.693832445	2.08E-07	4.69E-05	0.996518899	5.693832445	1.96E-07	4.55E-05	-0.049778766	5.693832445	0.795647373	1	0.020959721	5.693832445	0.897744151	1	blue
Slc43a3	0.932627044	6.841177894	1.09E-05	0.001241955	0.981802218	6.841177894	1.59E-05	0.00146734	-0.049848789	6.841177894	0.826147355	1	-0.000673827	6.841177894	0.995424324	1	blue
9530014B07Rik	1.027381456	3.906150805	0.000141556	0.008817184	1.067224967	3.906150805	0.00021988	0.011684579	-0.034711398	3.906150805	0.907589952	1	0.005130204	3.906150805	0.975684656	1	blue
Tsc22d3	1.025048963	7.140884203	1.74E-05	0.00171012	0.904293295	7.140884203	3.81E-04	0.017414254	0.062637347	7.140884203	0.804600698	1	-0.058118536	7.140884203	0.809367431	1	blue
Bcl2l1	0.761144059	6.352595794	2.28E-05	0.002056111	0.677614809	6.352595794	4.49E-04	0.019549867	0.052269607	6.352595794	0.786695108	1	-0.031259914	6.352595794	0.865249726	1	blue
Eef2k	0.739166459	5.442700054	1.22E-05	0.001364296	0.695700272	5.442700054	0.000129852	0.007740903	0.035696354	5.442700054	0.846064889	1	-0.007770333	5.442700054	0.969485194	1	blue
Smad6	0.807656829	4.460510346	7.20E-05	0.005134623	0.845848159	4.460510346	1.10E-04	0.006861752	0.024920405	4.460510346	0.911123278	1	0.063110521	4.460510346	0.743286091	1	blue
Serinc3	0.64973479	10.47118744	0.000718294	0.027963458	0.666110402	10.47118744	0.001287198	0.041643225	0.032613524	10.47118744	0.874264298	1	0.048989122	10.47118744	0.787557978	1	blue
Traf3ip2	0.644706446	5.006160563	0.00138326	0.044959173	0.657129911	5.006160563	2.47E-03	0.064647751	0.041822454	5.006160563	0.849302732	1	0.054245235	5.006160563	0.776367781	1	blue
Podxl	0.708886149	7.837561823	0.000134	0.008551552	0.777727049	7.837561823	0.000100416	0.0063217	0.037335683	7.837561823	0.851287383	1	0.106176469	7.837561823	0.551759386	1	blue
Trim62	0.971451816	4.189021359	0.000168783	0.009995399	1.188347569	4.189021359	1.83E-05	0.001653759	-0.05733611	4.189021359	0.839066256	1	0.159557869	4.189021359	0.513189338	1	blue
Ano1	0.66582106	8.102860229	0.000293472	0.014391437	0.887525204	8.102860229	7.95E-06	0.000860935	-0.088880744	8.102860229	0.652683948	1	0.132823326	8.102860229	0.454068494	1	blue
Prex1	0.677890082	6.159543276	0.000395094	0.017870005	0.832235186	6.159543276	5.53E-05	0.003901387	-0.06630753	6.159543276	0.747493402	1	0.088037297	6.159543276	0.631070916	1	blue
Jade2	0.54881478	5.266674911	0.002118084	0.060660546	0.643301609	5.266674911	8.64E-04	0.032218938	-0.037686101	5.266674911	8.48E-01	1	0.056800313	5.266674911	0.740238481	1	blue

0610039K10Rik	0.625035443	6.27843491	0.000109409	0.007281448	0.734482217	6.27843491	2.57E-05	0.002099505	-0.0331429	6.27843491	0.85056356	1	0.076303626	6.27843491	0.624244526	1	blue
Tbc1d8	0.562990795	5.412655912	0.001100272	0.037875373	0.678287465	5.412655912	2.74E-04	1.36E-02	-0.038457792	5.412655912	8.38E-01	1	0.076838472	5.412655912	0.643835541	1	blue
Pdgfra	0.720257488	7.702729003	1.07E-03	0.037054866	0.894398721	7.702729003	1.63E-04	9.14E-03	-0.091796918	7.702729003	6.97E-01	1	0.08234422	7.702729003	0.693635447	1	blue
Ucp2	0.994223124	8.719347437	1.16E-06	0.000207869	1.261927559	8.719347437	9.79E-09	3.51E-06	-0.143275036	8.719347437	0.509043749	1	0.124474335	8.719347437	0.521969097	1	blue
Amd1	0.994689935	6.873066056	1.36E-08	4.76E-06	1.391954758	6.873066056	1.89E-13	2.42E-10	-0.226743405	6.873066056	0.224356415	1	0.17052121	6.873066056	0.308459036	1	blue
Amd2	0.994689929	6.873066056	1.36E-08	4.76E-06	1.39195475	6.873066056	1.90E-13	2.42E-10	-0.226743392	6.873066056	0.224403344	1	0.170521222	6.873066056	0.30850766	1	blue
9130409J20Rik	3.633691877	1.981297266	3.23E-08	9.29E-06	5.731203838	1.981297266	5.64E-14	9.84E-11	-1.297162032	1.981297266	0.083943109	0.845870221	0.800412623	1.981297266	0.127935994	1	blue
Acot1	1.205886785	5.875248595	1.42E-05	0.001529395	1.799340142	5.875248595	2.15E-09	1.00E-06	-0.261782996	5.875248595	0.372347246	1	0.331669766	5.875248595	0.204762387	1	blue
Prr15l	0.747639304	3.225279928	0.005779624	0.120922188	1.141296031	3.225279928	0.000100038	0.0063217	-0.191973331	3.225279928	0.516155384	1	0.201681185	3.225279928	0.433383474	1	blue
F3	1.419687011	7.727166625	4.74E-07	9.75E-05	2.278594546	7.727166625	1.23E-13	1.90E-10	-0.376836861	7.727166625	0.200386683	1	0.48207049	7.727166625	0.067251409	1	blue
Erbb3	0.701868021	6.78890901	0.0004618	0.020187667	1.111513818	6.78890901	3.07E-07	6.55E-05	-0.127160678	6.78890901	0.554882959	1	0.282484885	6.78890901	0.143975386	1	blue
Pgf	0.679620726	3.128564667	0.012157716	0.191569999	1.118590638	3.128564667	0.00013349	0.007905338	-0.166553329	3.128564667	0.576059738	1	0.272413935	3.128564667	0.292367441	1	blue
Cirbp	0.530395862	4.952764904	0.003028263	0.078521342	0.946058689	4.952764904	1.07E-06	0.000180917	-0.147347106	4.952764904	0.44760616	1	0.268315073	4.952764904	0.121518797	1	blue
Gm12339	1.081412062	-0.224351867	0.018326512	0.240951394	1.874190423	-0.224351867	1.43E-04	0.008286404	-0.278401718	-0.224351867	0.656907768	1	0.514331146	-0.224351867	0.212954106	1	blue
Ccdc92	0.941406572	4.522393411	0.000156192	0.009449948	1.38829666	4.522393411	2.41E-07	5.36E-05	-0.070572705	4.522393411	0.795370681	1	0.376315643	4.522393411	0.112173623	1	blue
Tbx2	0.5399336	4.354707665	0.012029682	0.190404118	0.73441133	4.354707665	1.55E-03	0.04759797	0.032791581	4.354707665	0.890248802	1	0.227268025	4.354707665	0.273564031	1	blue
Iqgap2	0.645833926	5.373131927	0.001604521	0.050053101	0.867101693	5.373131927	8.70E-05	5.66E-03	0.028295778	5.373131927	8.99E-01	1	0.249562817	5.373131927	0.206261185	1	blue
Bex3	0.578548725	5.05985165	0.001295183	0.04309877	0.766950944	5.05985165	7.90E-05	0.005230217	-0.002956467	5.05985165	0.990298915	1	0.185445031	5.05985165	0.286432343	1	blue
Dgke	0.496537693	4.862039232	0.00424558	0.098729172	0.636978701	4.862039232	0.000692514	0.027187022	0.00518059	4.862039232	0.980346623	1	0.145620901	4.862039232	0.387421609	1	blue
Rnf208	0.927684806	2.955234052	0.00062105	0.025604116	1.282774597	2.955234052	1.02E-05	0.001017198	-0.064374074	2.955234052	0.82821698	1	0.290711023	2.955234052	0.254929041	1	blue
Galnt15	1.069569834	5.733849978	1.57E-08	5.00E-06	1.361127225	5.733849978	2.24E-11	1.74E-08	-0.017401078	5.733849978	0.933529324	1	0.274155508	5.733849978	0.128023189	1	blue
Acot4	1.090619856	3.100915954	0.003054407	0.078695583	1.403255774	3.100915954	0.000367164	0.017048125	-0.014033389	3.100915954	0.977190483	1	0.298597283	3.100915954	0.383609117	1	blue
Ccdc160	1.376366978	0.626861344	0.00135125	0.044435459	1.473182592	0.626861344	0.001200993	0.039845004	0.136965239	0.626861344	0.793355156	1	0.233739536	0.626861344	0.543008501	1	blue
Spock2	1.214006164	5.008459561	6.45E-09	2.50E-06	1.261938539	5.008459561	1.53E-08	5.33E-06	0.149303583	5.008459561	0.502236944	1	0.197234272	5.008459561	0.317760217	1	blue
Tmprss2	1.306912053	7.443328782	0.000361864	0.016858051	1.386593368	7.443328782	3.74E-04	0.017246677	0.180993704	7.443328782	0.636635374	1	0.260674656	7.443328782	0.443009178	1	blue
Rdh12	1.414644844	0.551824247	0.001454363	0.046625615	1.447718236	0.551824247	1.80E-03	0.053376774	0.219193509	0.551824247	0.674471731	1	0.252218053	0.551824247	0.527543254	1	blue
Fzd6	0.737725683	5.429315892	0.000135155	0.008586048	0.698317754	5.429315892	7.71E-04	0.029535506	0.135127911	5.429315892	0.515881226	1	0.095719269	5.429315892	0.604775938	1	blue
Pla2g12a	0.90681715	5.637984645	0.000282544	0.014002965	0.863740076	5.637984645	1.23E-03	0.040387032	0.16746133	5.637984645	0.529787115	1	0.124383464	5.637984645	0.598708065	1	blue
Fmo2	0.933919293	6.274333397	2.60E-05	0.002188418	0.907078902	6.274333397	0.000135198	0.007972717	0.188641541	6.274333397	0.425074982	1	0.161800592	6.274333397	0.444315026	1	blue
E030018B13Rik	3.464045181	-1.192186001	2.50E-05	0.002117227	2.830144939	-1.192186001	0.000202128	0.01090713	1.168475186	-1.192186001	0.262569411	1	0.533388123	-1.192186001	0.358891106	1	blue
Doc2b	2.025487909	4.173270047	9.59E-19	1.91E-15	1.848847518	4.173270047	7.17E-15	1.43E-11	0.544489981	4.173270047	0.022699887	0.512474439	0.367841776	4.173270047	0.073886315	1	blue
Adgrf5	0.756326259	9.345786667	0.000711696	0.027861808	0.660663506	9.345786667	5.85E-03	0.112480452	0.198353069	9.345786667	0.406243206	1	0.102690264	9.345786667	0.629405073	1	blue
Lncbate1	1.111203802	3.651767409	0.00037831	0.017392296	1.014945315	3.651767409	0.002210222	0.060568758	0.265232528	3.651767409	0.424760762	1	0.168969704	3.651767409	0.561943554	1	blue
Cntfr	1.348822572	4.770386185	5.06E-10	2.44E-07	1.17533991	4.770386185	3.09E-07	6.55E-05	0.267259614	4.770386185	0.244754834	1	0.093774685	4.770386185	0.642900525	1	blue
Vipr1	1.865192981	5.658334984	1.13E-19	3.94E-16	1.706052177	5.658334984	2.44E-15	6.72E-12	0.312816745	5.658334984	0.141518768	0.992645357	0.153673996	5.658334984	0.414320507	1	blue
Timp3	0.964779928	9.688689281	1.49E-05	0.001552812	0.8206474	9.688689281	5.61E-04	0.023459467	0.186989227	9.688689281	0.429035901	1	0.042856651	9.688689281	0.834568293	1	blue
Acot5	4.99615376	2.312573422	1.61E-13	1.61E-10	4.914911385	2.312573422	3.89E-13	4.53E-10	0.550689052	2.312573422	0.476930225	1	0.4692304	2.312573422	0.341469962	1	blue
Lnx1	0.587212079	5.214133977	0.000741044	0.028689287	0.542338226	5.214133977	0.0038743	0.086359187	0.064586598	5.214133977	0.731965938	1	0.01971222	5.214133977	0.903036299	1	blue
Rhpn2	0.84201561	4.691028512	7.93E-05	0.005540888	0.72597817	4.691028512	0.001500221	0.046381353	0.113267491	4.691028512	0.622098178	1	-0.002771064	4.691028512	0.997922404	1	blue
Fkbp5	2.741350376	6.984434929	3.61E-19	1.01E-15	1.96172975	6.984434929	2.45E-10	1.56E-07	0.431172357	6.984434929	0.151956911	0.999246012	-0.348449505	6.984434929	0.209942093	1	blue
E030003E18Rik	0.854441344	4.264958251	0.000756522	0.029047101	0.568917148	4.264958251	3.59E-02	3.06E-01	0.147348207	4.264958251	0.59004117	1	-0.138177251	4.264958251	0.583885415	1	blue
Fam117b	0.706367958	5.753259135	1.85E-05	0.001783651	0.479663658	5.753259135	0.006779411	0.122414783	0.154093538	5.753259135	0.386075393	1	-0.072611179	5.753259135	0.658779714	1	blue
Tead1	1.397241814	7.609702097	3.76E-07	7.97E-05	0.997158198	7.609702097	5.69E-04	2.37E-02	0.292594769	7.609702097	0.308245442	1	-0.107489132	7.609702097	0.690675588	1	blue
Rbm3	0.733808345	6.733015213	2.11E-05	0.002008606	0.451102219	6.733015213	0.014794549	0.189493731	0.204571963	6.733015213	0.269222257	1	-0.078134399	6.733015213	0.649165818	1	blue
Mertk	1.029015989	5.358791334	8.45E-10	3.94E-07	0.692586367	5.358791334	1.05E-04	0.006563851	0.250241159	5.358791334	0.162508213	1	-0.086189385	5.358791334	0.600869351	1	blue
Mcf2l	0.83229974	6.388578383	0.000143832	0.008894676	0.633882399	6.388578383	0.006835106	0.123102379	0.165011488	6.388578383	0.480413241	1	-0.033406201	6.388578383	0.883462482	1	blue
Pgm3	0.590573941	4.653485038	0.000761285	0.02914992	0.462073461	4.653485038	1.45E-02	0.188221265	0.128915585	4.653485038	0.49796455	1	0.000414263	4.653485038	0.993461078	1	blue
Rab27a	0.718368643	3.628015463	0.001567823	0.049240227	0.491932192	3.628015463	0.043506771	0.342557397	0.183610962	3.628015463	0.456858875	1	-0.042827555	3.628015463	0.856943214	1	blue
Pkp4	0.656449803	7.60955638	0.000998895	0.034988847	0.454878617	7.60955638	3.38E-02	0.296604626	0.184254245	7.60955638	0.389460298	1	-0.017317067	7.60955638	0.936664876	1	blue
Bdh1	1.1512702	2.954224809	0.000698818	0.02779717	0.864386975	2.954224809	1.60E-02	0.196427164	0.273057185	2.954224809	0.450661204	1	-0.013832208	2.954224809	0.981520631	1	blue
Hif3a	2.58019991	1.987878999	1.77E-11	1.18E-08	1.898632389	1.987878999	8.66E-07	1.51E-04	0.679656229	1.987878999	0.092116638	0.875797372	-0.001958094	1.987878999	1	1	blue
Vwa3a	1.039689419	3.848365904	0.000318916	0.015212199	0.766740901	3.848365904	1.26E-02	0.173063789	0.270758054	3.848365904	0.379427457	1	-0.002193472	3.848365904	0.997654322	1	blue
Arg2	1.332911746	5.95491937	4.80E-10	2.40E-07	1.089840356	5.95491937	1.47E-06	0.000230104	0.496280422	5.95491937	0.027615363	0.560163016	0.253207721	5.95491937	0.208598756	1	blue
Trim24	0.613798861	6.257363855	7.57E-05	0.005315624	0.481248156	6.257363855	3.94E-03	0.087730907	0.249802356	6.257363855	0.13504958	0.984073476	0.11725123	6.257363855	0.435074514	1	blue
Tfap2b	0.979803269	6.429155404	0.000141948	0.008817184	0.724477194	6.429155404	0.008211143	0.137643065	0.4779401	6.429155404	0.080897499	0.836876692	0.222613316	6.429155404	0.362612436	1	blue
Spdef	2.261113551	3.424095102	5.70E-19	1.33E-15	1.702739923	3.424095102	4.46E-11	3.12E-08	0.751215362	3.424095102	0.004548646	0.215497896	0.192826116	3.424095102	0.38815111	1	blue
Mchr1	2.883502982	1.180841908	0.000341136	0.016161749	2.078304758	1.180841908	0.009909756	0.151199508	0.977463862	1.180841908	0.22409868	1	0.172144887	1.180841908	0.783130598	1	blue
Picalm	0.768824764	8.935796305	0.000192816	0.010600388	0.495489458	8.935796305	0.024847809	0.24852317	0.253816156	8.935796305	0.249697144	1	-0.019519214	8.935796305	0.930128978	1	blue
Hacd2	0.977915599	7.660755962	0.000990013	0.03476489	0.706642178	7.660755962	0.025287272	0.251094724	0.304083644	7.660755962	0.334094822	1	0.032810006	7.660755962	0.898503866	1	blue
Acsl1	1.098454325	11.8545152	3.15E-04	0.015184271	0.747139421	11.8545152	2.08E-02	2.24E-01	0.388057345	11.8545152	0.227872521	1	0.036742426	11.8545152	0.889478115	1	blue
Acer2	0.72953022	6.388383381	0.000376462	0.017364469	0.494453544	6.388383381	0.024595767	0.246947158	0.297585942	6.388383381	0.176017921	1	0.062508843	6.388383381	0.747885643	1	blue
Rab11fip4	1.537157873	3.176872231	2.70E-08	8.40E-06	0.98429268	3.176872231	0.000600963	0.024703133	0.658248964	3.176872231	0.023950542	0.527240563	0.105373327	3.176872231	0.674886713	1	blue
Ttll7	1.205549868	4.189772768	8.13E-07	0.000153625	0.681226457	4.189772768	8.25E-03	1.38E-01	0.567931847	4.189772768	0.028729546	0.566324582	0.043604768	4.189772768	0.844653058	1	blue
Cry2	0.698059413	6.102482994	1.09E-05	0.001241955	0.406786363	6.102482994	1.69E-02	2.02E-01	0.283252501	6.102482994	0.097076404	0.888392991	-0.00802099	6.102482994	0.964923332	1	blue
Gramd1b	1.417544479	4.711567254	9.24E-08	2.35E-05	0.814560378	4.711567254	3.42E-03	0.079854772	0.545355622	4.711567254	0.050665479	0.711157815	-0.057631229	4.711567254	0.828998008	1	blue
Lrrn1	1.566323401	1.945104228	4.48E-06	0.000632226	0.719681992	1.945104228	4.04E-02	0.32867894	0.676338939	1.945104228	0.062566578	0.771874186	-0.17032379	1.945104228	0.60582684	1	blue
Atl2	0.952854283	7.119014985	0.000776858	0.029423775	0.416545975	7.119014985	0.166452847	0.627980926	0.385547333	7.119014985	0.200466321	1	-0.150761242	7.119014985	0.592510659	1	blue
Ccdc85a	0.844465458	4.89222276	0.000260985	0.013167964	0.258826256	4.89222276	0.294587729	0.765128804	0.390124727	4.89222276	0.11508933	0.935167722	-0.195515502	4.89222276	0.392347115	1	blue
Hpgd	0.970325176	4.330839936	3.00E-06	0.00047158	0.241566394	4.330839936	0.275541634	0.74699056	0.371677524	4.330839936	0.094851886	0.884946568	-0.357082572	4.330839936	0.079872496	1	blue
Adh1	0.728061415	6.974220274	0.000412052	0.018457822	0.359078623	6.974220274	0.103935757	0.518417611	0.268675727	6.974220274	0.223831906	1	-0.100307275	6.974220274	0.625510121	1	blue
4930523C07Rik	0.736666591	6.375915302	2.90E-05	0.002384562	0.436197927	6.375915302	2.09E-02	0.225346693	0.216509725	6.375915302	0.252321817	1	-0.083959245	6.375915302	0.632029343	1	blue
Arxes1	1.760851126	5.26525857	8.16E-08	2.11E-05	1.027076514	5.26525857	0.002531766	0.065519286	0.481440157	5.26525857	0.155505941	1	-0.252336512	5.26525857	0.421832539	1	blue
4930562F07Rik	0.806162095	4.461274437	8.04E-05	0.005585307	0.399476353	4.461274437	0.067824736	0.425839405	0.265935769	4.461274437	0.226922817	1	-0.140751203	4.461274437	0.486722652	1	blue
Tmem52	2.476352048	0.166329032	3.26E-06	0.000494542	1.257996319	0.166329032	1.87E-02	2.14E-01	0.703718664	0.166329032	0.254063688	1	-0.514763581	0.166329032	0.279130478	1	blue
Sult1a1	1.024284651	7.632797681	1.07E-06	0.000194915	0.507517797	7.632797681	2.28E-02	2.37E-01	0.261047114	7.632797681	0.241098505	1	-0.255719888	7.632797681	0.213827337	1	blue
Hhipl1	2.113611244	1.606635072	9.43E-09	3.56E-06	1.149638766	1.606635072	0.002199004	0.060498574	0.470240158	1.606635072	0.225812814	1	-0.493762104	1.606635072	0.14457258	1	blue
Map3k6	1.287435331	6.460252583	1.03E-09	4.64E-07	0.808007505	6.460252583	0.000282698	0.013911939	0.147575206	6.460252583	0.506328868	1	-0.33185298	6.460252583	0.104497072	1	blue
Ercc1	0.840560109	5.152430037	4.43E-06	0.000632226	0.531487274	5.152430037	0.006756364	0.122156469	0.115442559	5.152430037	0.557773102	1	-0.193630828	5.152430037	0.282665678	1	blue
Arxes2	1.236206346	6.988556664	0.000313147	0.015143735	0.909743869	6.988556664	0.012039716	0.169112635	0.155903629	6.988556664	0.664942424	1	-0.170559146	6.988556664	0.614956151	1	blue
Arhgap27	0.593845778	5.60393262	0.000259491	0.013140009	0.401428594	5.60393262	2.20E-02	0.233349447	0.077564332	5.60393262	0.659192754	1	-0.114853135	5.60393262	0.477327367	1	blue
Dlg3	0.911285371	5.316144596	1.72E-07	4.08E-05	0.665745686	5.316144596	3.59E-04	0.016766799	0.072303573	5.316144596	0.700495246	1	-0.173236647	5.316144596	0.311306866	1	blue
Hmgcs2	2.24146939	7.089566034	6.43E-08	1.73E-05	2.088628135	7.089566034	1.31E-06	2.10E-04	-0.075307505	7.089566034	0.856893105	1	-0.228149285	7.089566034	0.560417175	1	blue
Arhgef37	1.29507907	2.438323921	2.18E-05	0.002033323	1.126115503	2.438323921	5.04E-04	0.021477797	0.017721178	2.438323921	0.967397572	1	-0.151248704	2.438323921	0.614890737	1	blue
Adam12	0.877406941	6.960743112	1.34E-06	0.00022812	0.717527788	6.960743112	0.000222773	0.011793482	0.035207002	6.960743112	0.856054599	1	-0.124672317	6.960743112	0.486019059	1	blue
Aldh1a1	0.917452432	6.413295798	5.03E-06	0.00070313	0.759955633	6.413295798	0.000408042	0.018219807	0.051680254	6.413295798	0.810405453	1	-0.105816824	6.413295798	0.594528476	1	blue
Abcg2	0.736612966	5.879003949	2.97E-05	0.002413018	0.578880435	5.879003949	0.002262834	0.061159018	0.028454261	5.879003949	0.881603582	1	-0.129278537	5.879003949	0.459173725	1	blue
Asph	0.831581955	7.39324062	3.17E-04	0.015212199	0.618950798	7.39324062	1.22E-02	1.70E-01	0.026461988	7.39324062	0.914828798	1	-0.186169264	7.39324062	0.4150765	1	blue
Arg1	2.599297591	3.184737288	1.99E-04	0.010728997	1.685831854	3.184737288	1.66E-02	0.200276785	0.135037271	3.184737288	0.848350079	1	-0.778437985	3.184737288	0.231775325	1	blue
Gimap3	1.063428819	3.005087274	7.15E-04	2.79E-02	0.729233654	3.005087274	2.96E-02	0.276732073	-0.023909242	3.005087274	0.950697699	1	-0.358106065	3.005087274	0.245829394	1	blue
Slc2a3	0.993676461	3.241694228	1.33E-04	8.51E-03	0.66442156	3.241694228	1.66E-02	2.00E-01	0.008220896	3.241694228	0.981768332	1	-0.321035516	3.241694228	0.207279624	1	blue
Rpp25	2.278147397	-0.250095948	4.13E-06	0.000613971	1.207912568	-0.250095948	0.019524004	0.21742429	0.341661177	-0.250095948	0.588423835	1	-0.72867341	-0.250095948	0.101407825	1	blue
Fam166a	0.771775767	3.611245376	0.000528338	0.022512343	0.297786812	3.611245376	0.213940314	0.693903418	0.171888957	3.611245376	0.476602723	1	-0.302101142	3.611245376	0.169706452	1	blue
Tubb4b	0.768170116	7.747087276	1.37E-05	0.001490146	0.3907997	7.747087276	3.88E-02	0.321259087	0.080042341	7.747087276	0.672188633	1	-0.297328124	7.747087276	0.088266485	1	blue
Acta1	1.43402517	4.939937223	0.000743343	0.028698784	0.86456728	4.939937223	0.052021266	0.376355629	0.023737323	4.939937223	0.958856051	1	-0.545721245	4.939937223	0.187627896	1	blue
Ramp2	0.702589498	5.906020011	8.73E-05	0.005951449	0.454613955	5.906020011	0.018299233	0.212064741	-0.061453976	5.906020011	0.751188386	1	-0.309429557	5.906020011	0.080827951	1	blue
Itga3	0.6580401	6.249348178	7.55E-05	0.005315624	0.39473008	6.249348178	0.027424771	0.261988107	-0.087201042	6.249348178	0.626631195	1	-0.350511033	6.249348178	0.03350442	0.745544874	blue
Mmp28	1.094307568	5.849210985	1.72E-04	1.01E-02	0.451523702	5.849210985	1.43E-01	5.93E-01	0.041758492	5.849210985	0.892856918	1	-0.601025459	5.849210985	0.036100846	0.776223732	blue
Hsd11b2	1.917871981	1.322557591	3.44E-07	7.50E-05	0.65028404	1.322557591	0.100891488	0.512004154	0.175032262	1.322557591	0.67822458	1	-1.092566483	1.322557591	0.002415982	0.167988845	blue
1700020L24Rik	1.023541402	3.887623509	1.86E-04	0.010600388	0.259129067	3.887623509	0.375610062	0.828442549	0.105297158	3.887623509	0.720854792	1	-0.659115283	3.887623509	0.014875005	0.498843884	blue
Snta1	1.03526562	5.022342532	1.83E-04	0.010529899	0.322940749	5.022342532	0.271756433	0.743989796	0.075279219	5.022342532	0.7990293	1	-0.637045716	5.022342532	0.019679916	0.577753327	blue
Prrg3	1.037385269	4.845047341	1.33E-05	0.001458677	0.309956759	4.845047341	0.221293684	0.702201142	0.062612723	4.845047341	0.806684621	1	-0.66481579	4.845047341	0.004687674	0.258952313	blue
Abhd2	0.776396415	6.908758217	1.02E-05	0.001197305	0.274448673	6.908758217	0.145533784	0.596864298	-0.095698467	6.908758217	0.612231282	1	-0.597646111	6.908758217	0.000634225	0.065175918	blue
Prr5	0.75734972	5.323788412	1.59E-03	0.04980942	0.288860176	5.323788412	0.261824677	0.736543853	-0.189601415	5.323788412	0.461729983	1	-0.658090476	5.323788412	0.005932723	0.299640059	blue
Dmrt2	0.940741633	5.309269374	2.68E-04	0.013431649	0.244373989	5.309269374	0.374462263	0.827428394	-0.008292443	5.309269374	0.977323188	1	-0.704659869	5.309269374	0.005938773	0.299640059	blue
Prap1	7.803310191	-2.087893825	6.58E-04	0.026581036	4.434376539	-2.087893825	0.173499692	0.639846002	0	-2.087893825	1	1	-3.38311564	-2.087893825	0.023868007	0.639040746	blue
Bves	1.433830038	0.57218631	0.001156344	0.039321333	0.075472858	0.57218631	0.906951377	0.998463035	0.147108337	0.57218631	0.780481055	1	-1.211239833	0.57218631	0.005385898	0.280832869	blue
Bmper	0.820031452	5.099432266	1.52E-04	0.009306445	-0.044539625	5.099432266	0.848230697	0.983893751	-0.007023704	5.099432266	0.97867452	1	-0.871594107	5.099432266	5.87E-05	0.00976082	blue
Aldh1l2	0.833127391	2.761028558	3.67E-03	8.99E-02	-0.25468257	2.761028558	0.414311295	0.853665733	0.026597756	2.761028558	9.36E-01	1.00E+00	-1.061207162	2.761028558	0.00025176	0.031987248	blue
Sprr2f	8.117505927	-1.779938808	4.99E-04	0.021539862	-1.766546245	-1.779938808	0.354680408	0.812264563	4.993459983	-1.779938808	0.080194858	0.836876692	-4.920393125	-1.779938808	0.003968093	0.233017068	blue
Ldlr	1.016052091	5.593230623	6.81E-07	0.000132197	-0.082622825	5.593230623	0.704709936	0.960193977	0.604783742	5.593230623	5.54E-03	0.243490854	-0.493891781	5.593230623	0.01402656	0.488308544	green
Clca1	1.852552531	5.550276755	6.29E-05	0.00453325	-0.136540547	5.550276755	0.772489798	0.972503068	1.303376697	5.550276755	6.59E-03	0.267790873	-0.685718511	5.550276755	0.122577709	1	green
Zbtb16	1.004343291	6.548549971	1.30E-04	8.40E-03	0.176369804	6.548549971	0.525985422	0.900217029	0.580714704	6.548549971	3.74E-02	6.44E-01	-0.247259231	6.548549971	3.38E-01	1.00E+00	green
Fam90a1a	4.256346294	0.966201982	2.18E-05	0.002033323	0.663652211	0.966201982	0.457654701	0.874323659	2.871424415	0.966201982	4.00E-03	2.03E-01	-0.721666108	0.966201982	3.98E-01	1	green
Hlf	1.159210537	4.589951861	1.42E-07	3.55E-05	0.266468335	4.589951861	0.250681124	0.725724931	0.75792243	4.589951861	0.001226394	0.09733151	-0.134822388	4.589951861	0.531423959	1	green
Slc1a1	1.005737135	3.255130293	1.25E-04	8.24E-03	0.268543444	3.255130293	3.34E-01	0.795715899	0.612735268	3.255130293	2.93E-02	5.74E-01	-0.124463666	3.255130293	0.632370232	1	green
Timp4	1.599184426	6.059068423	3.44E-10	1.85E-07	0.665501909	6.059068423	1.18E-02	1.67E-01	0.826222171	6.059068423	1.87E-03	0.127362287	-0.107461763	6.059068423	6.63E-01	1	green

Elmod1	2.406245051	0.950833131	2.18E-05	0.002033323	0.781485552	0.950833131	1.60E-01	0.620149041	1.455151054	0.950833131	0.013197346	0.39495954	-0.169715658	0.950833131	7.59E-01	1	green
Dbp	2.128361701	5.647384536	7.82E-17	1.21E-13	0.743554083	5.647384536	4.02E-03	8.93E-02	1.474926118	5.647384536	2.23E-08	1.56E-05	0.090114678	5.647384536	6.93E-01	1.00E+00	green
Defb1	3.176162358	4.056296796	2.43E-05	0.002094958	0.705823803	4.056296796	0.324857327	0.790409512	2.375234483	4.056296796	0.001605694	0.114495797	-0.095129588	4.056296796	0.907979067	1	green
Vldlr	0.581999053	6.404520307	0.000470358	0.020352075	-0.105014263	6.404520307	0.557306874	0.907588076	0.636264214	6.404520307	0.000399086	0.044672631	-0.050749528	6.404520307	0.762209753	1	green
Per3	0.712943419	4.720701508	0.000215636	0.011203457	-0.007753704	4.720701508	9.72E-01	1	0.956325054	4.720701508	4.17E-06	0.001388235	0.235625319	4.720701508	2.04E-01	1	green
Ucp1	4.311951949	8.789550622	2.98E-15	4.16E-12	1.517570922	8.789550622	2.41E-03	0.0636901	5.652026585	8.789550622	2.88E-21	2.01E-17	2.857636409	8.789550622	2.06E-10	1.52E-07	green
Atp1b1	0.635062842	5.033336671	0.002394235	0.066261049	0.115990723	5.033336671	6.04E-01	0.925246103	0.789886023	5.033336671	0.000462949	0.049390678	0.27081204	5.033336671	1.79E-01	1	green
Wnk2	0.856611446	4.771147706	1.55E-05	0.001581754	0.209134461	4.771147706	3.20E-01	0.787720626	0.874947373	4.771147706	3.97E-05	0.008958835	0.227467745	4.771147706	2.31E-01	1	green
Slc35f3	0.838419748	4.19440086	0.001487395	0.047245074	0.194894168	4.19440086	0.487341885	0.886860701	0.827005398	4.19440086	0.003469077	0.185343843	0.183476158	4.19440086	4.64E-01	1	green
Hecw2	0.790663299	4.248254429	0.00036825	0.017098568	0.189034088	4.248254429	0.425144386	0.859628985	0.732599653	4.248254429	0.00212231	0.136532201	0.130967375	4.248254429	5.36E-01	1	green
Nr1d2	0.885924594	6.592266291	1.71E-06	0.00028774	0.327181345	6.592266291	0.096831314	0.502530428	0.836251395	6.592266291	2.52E-05	0.006300986	0.277507373	6.592266291	1.18E-01	1.00E+00	green
Hmcn1	1.069436917	5.400113035	8.20E-05	0.005648787	0.5687602	5.400113035	0.047392885	0.357507289	0.995159423	5.400113035	5.91E-04	0.059434824	0.494480251	5.400113035	5.43E-02	9.34E-01	green
Zim1	1.247933662	4.085438773	0.000140958	0.008817184	0.965365938	4.085438773	0.004987724	0.102287048	1.066616002	4.085438773	2.18E-03	0.139031142	0.784040218	4.085438773	1.03E-02	4.05E-01	green
Mrgprg	3.993118823	2.043643367	2.22E-10	1.29E-07	2.787451587	2.043643367	4.80E-07	9.72E-05	3.565532196	2.043643367	2.73E-08	1.82E-05	2.359363328	2.043643367	5.11E-07	1.93E-04	green
Ppargc1a	1.982534947	5.435797876	7.55E-11	4.59E-08	1.773714812	5.435797876	2.07E-08	7.05E-06	1.754274958	5.435797876	3.68E-08	2.23E-05	1.545446915	5.435797876	2.22E-08	1.11E-05	green
Clstn3	1.064770476	6.041001421	1.28E-06	0.000221195	0.984565873	6.041001421	2.57E-05	0.002099505	0.872248158	6.041001421	0.000197947	0.028229657	0.792041809	6.041001421	1.44E-04	0.020316334	green
Dio2	3.251687223	7.214316121	3.16E-21	1.47E-17	2.39748024	7.214316121	2.82E-12	2.46E-09	2.175785787	7.214316121	1.89E-10	2.20E-07	1.321574126	7.214316121	6.28E-06	0.001720873	green
Agtr1a	1.786134508	6.823663073	1.01E-14	1.28E-11	1.061156796	6.823663073	8.82E-06	0.000912587	1.200177633	6.823663073	5.84E-07	0.000282839	0.475198441	6.823663073	2.54E-02	0.667031241	green
Usp2	1.047880391	4.768329828	1.73E-06	0.00028774	0.596314893	4.768329828	0.010090581	0.15213157	0.670113928	4.768329828	0.00415223	0.205785718	0.218545763	4.768329828	2.93E-01	1	green
Pde4c	1.351227457	1.537245209	0.000187769	0.010600388	0.635901299	1.537245209	0.087266143	0.481270409	1.025435112	1.537245209	0.008273823	0.303503793	0.310069874	1.537245209	3.47E-01	1	green
Gm5627	1.900664118	1.012341782	2.46E-05	0.002109815	0.917482479	1.012341782	0.03873415	0.320704074	1.524252488	1.012341782	0.001379506	0.103101487	0.540967895	1.012341782	1.68E-01	1	green
Cpm	1.196917847	3.45537694	3.68E-06	0.000552435	0.486521238	3.45537694	0.071721808	0.438387547	0.895618987	3.45537694	0.001158552	0.093594892	0.185214254	3.45537694	4.44E-01	1	green
Per2	2.064782704	5.563537741	7.39E-24	5.16E-20	0.946938331	5.563537741	5.68E-06	0.000666561	1.270404265	5.563537741	2.10E-09	2.10E-06	0.152556153	5.563537741	4.12E-01	1	green
Plxna2	0.696618996	6.494677677	0.000404515	0.018178471	0.293896068	6.494677677	0.163873002	0.624805269	0.418893276	6.494677677	4.76E-02	0.694640431	0.016169942	6.494677677	9.27E-01	1	green
Tef	1.476517336	7.233668497	4.07E-12	2.84E-09	0.790101395	7.233668497	0.000384084	0.017485204	0.854823149	7.233668497	1.28E-04	0.020511795	0.16840652	7.233668497	3.97E-01	1.00E+00	green
Myzap	0.768756507	4.432975696	0.000388946	0.017706528	0.436775049	4.432975696	0.059482742	0.399327317	0.413771251	4.432975696	7.56E-02	8.18E-01	0.081787823	4.432975696	6.92E-01	1.00E+00	green
Coro2a	0.827407998	7.521081466	2.43E-05	0.002094958	0.874068987	7.521081466	3.28E-05	0.002561752	0.393697096	7.521081466	6.02E-02	7.61E-01	0.440357751	7.521081466	1.94E-02	5.70E-01	purple
Wnt4	0.784124639	5.881931281	0.001248352	0.041739146	0.868194562	5.881931281	0.00087422	0.032494946	0.316077464	5.881931281	2.24E-01	1.00E+00	0.400146464	5.881931281	8.58E-02	1.00E+00	purple
Ciart	2.362758445	2.160857684	1.16E-09	5.07E-07	2.312332835	2.160857684	4.94E-09	1.97E-06	0.867541103	2.160857684	3.24E-02	0.608175827	0.817060862	2.160857684	1.39E-02	0.488308544	purple
Cutal	1.289054787	3.371331092	3.07E-06	0.000476209	1.261300666	3.371331092	1.48E-05	0.001392839	0.476706941	3.371331092	1.03E-01	9.06E-01	0.448944277	3.371331092	8.03E-02	1.00E+00	purple
Rev3l	1.041603752	7.181238286	8.50E-06	0.001052705	1.037035155	7.181238286	3.33E-05	0.002571221	0.324719276	7.181238286	1.90E-01	1.00E+00	0.320150255	7.181238286	1.50E-01	1.00E+00	purple
Nup210	0.685373938	5.540092682	0.000769792	0.029395112	0.697846612	5.540092682	0.001442488	0.045303862	0.230396176	5.540092682	0.29306043	1	0.242867995	5.540092682	0.21605994	1	purple
P2ry14	1.135357851	2.766842375	0.000130352	0.008395362	1.232878239	2.766842375	8.62E-05	0.005641246	0.317565677	2.766842375	3.22E-01	1.00E+00	0.415075705	2.766842375	1.33E-01	1.00E+00	purple
Setx	0.919954055	7.036280574	6.45E-06	0.000858248	1.085964455	7.036280574	7.16E-07	0.000130057	0.261463451	7.036280574	2.29E-01	1.00E+00	0.427473404	7.036280574	2.85E-02	6.97E-01	purple
Rnf103	0.581804773	6.1458953	0.000354	0.016602381	0.665811108	6.1458953	0.000151706	0.008603816	0.17261572	6.1458953	3.26E-01	1.00E+00	0.256621567	6.1458953	1.04E-01	1.00E+00	purple
Penk	0.65869557	5.238105193	0.002206391	0.06229601	0.747870251	5.238105193	0.00124044	0.040600459	0.137026943	5.238105193	5.55E-01	1.00E+00	0.226200717	5.238105193	2.75E-01	1.00E+00	purple
Sox9	0.933093415	6.487667883	0.000444719	0.019726362	1.047910591	6.487667883	0.000233166	0.012069366	0.198613453	6.487667883	0.482387252	1	0.313430064	6.487667883	0.215281857	1	purple
Bmf	0.97100521	4.875736996	2.88E-08	8.57E-06	1.04186115	4.875736996	2.61E-08	7.94E-06	0.207813913	4.875736996	0.269173209	1	0.278668115	4.875736996	0.095035674	1	purple
Pik3ip1	1.289287561	4.20961172	1.55E-08	5.00E-06	1.535313666	4.20961172	2.96E-10	1.79E-07	0.102872295	4.20961172	0.673664811	1	0.348895046	4.20961172	0.102852915	1	purple
Lifr	0.618501836	7.364552415	0.000801655	0.030118099	0.722860275	7.364552415	0.00028228	0.013911939	0.097390004	7.364552415	6.24E-01	1	0.201748261	7.364552415	0.258715847	1	purple
Cldn3	0.660791436	6.275978654	0.000190773	0.010600388	0.820213712	6.275978654	1.77E-05	0.001613007	0.088526489	6.275978654	6.43E-01	1	0.247948337	6.275978654	0.147764778	1	purple
Kcnj11	1.031889361	2.193547139	0.001440683	0.046393969	1.337838714	2.193547139	0.000104904	0.006563851	0.080880443	2.193547139	0.826093992	1	0.38681839	2.193547139	0.197260173	1	purple
Ppp1r1b	1.217769289	2.384261146	0.000710706	0.027861808	1.646155066	2.384261146	1.86E-05	0.001663136	0.092414238	2.384261146	0.822864263	1	0.520787257	2.384261146	0.117333955	1	purple
B4galnt1	0.944127244	6.161515422	1.69E-05	0.001686219	1.276210369	6.161515422	6.75E-08	1.85E-05	0.0789009	6.161515422	0.736253451	1	0.410983332	6.161515422	0.049845827	0.901682314	purple
Tceal9	0.570459844	6.620303944	0.001063529	0.037054866	0.80178076	6.620303944	2.11E-05	0.001818951	0.051457285	6.620303944	0.784722151	1	0.282777891	6.620303944	0.094517883	1	purple
Cyp1b1	0.757259222	5.756235502	0.000259234	0.013140009	1.014522464	5.756235502	5.61E-06	0.000666561	0.101742014	5.756235502	0.648331565	1	0.359004481	5.756235502	0.071630281	1	purple
Slc25a33	0.836046219	4.353422245	0.003059906	0.078695583	1.267566173	4.353422245	3.07E-05	0.002437658	0.06549663	4.353422245	0.831644656	1	0.497014188	4.353422245	0.064395693	1	purple
1700003D09Rik	1.316800605	-0.002439808	0.004215143	0.098246499	1.976428455	-0.002439808	4.51E-05	0.003284714	0.037495587	-0.002439808	0.952126885	1	0.697045575	-0.002439808	0.088012738	1	purple
Kcnip3	1.378648895	3.675972929	3.47E-05	0.002747378	2.268615128	3.675972929	3.30E-10	1.85E-07	0.018610211	3.675972929	0.965101772	1	0.908569511	3.675972929	0.003125399	0.199303796	purple
Mrgpre	0.933077231	1.996623469	0.010587745	0.175533	1.324738439	1.996623469	6.96E-04	0.027241824	0.148817785	1.996623469	0.707488281	1	0.540464216	1.996623469	0.113109439	1	purple
Lox	0.598161484	6.774435352	0.00362647	0.089231596	0.795125779	6.774435352	0.000342369	0.016220179	0.125217005	6.774435352	0.571640596	1	0.32218097	6.774435352	0.105317615	1	purple
Adcyap1r1	0.429737764	5.084990331	0.019562026	0.252445862	0.644776236	5.084990331	0.001203105	0.039845004	0.091361546	5.084990331	0.647378981	1	0.306399162	5.084990331	0.086852978	1	purple
Zfp664	0.830175952	8.366031636	6.64E-05	0.004759724	1.200112453	8.366031636	9.50E-08	2.40E-05	0.178568728	8.366031636	0.421378392	1	0.548505058	8.366031636	0.006030876	0.302208908	purple
Stard5	0.468733483	4.390234073	0.013069296	0.199421077	0.652084402	4.390234073	0.001362177	0.043465266	0.161427735	4.390234073	0.431079811	1	0.344776998	4.390234073	0.059359914	0.965078208	purple
Hpdl	1.588182628	1.241813226	0.000153292	0.009355518	2.449632198	1.241813226	2.16E-08	7.20E-06	0.380733695	1.241813226	0.395913419	1	1.242118267	1.241813226	0.000694459	0.070153271	purple
Cyp2d22	0.38487714	5.45947463	0.027142116	0.304444795	0.59937209	5.45947463	1.48E-03	4.60E-02	0.156702358	5.45947463	0.406127204	1	0.371196552	5.45947463	0.02855736	0.696763009	purple
Adcy3	1.004320212	6.541174635	0.000163164	0.00970377	1.743920244	6.541174635	1.74E-09	8.38E-07	0.225419169	6.541174635	0.425293482	1	0.96501827	6.541174635	0.000133907	0.01909671	purple
Cenpo	0.70559601	4.84676238	0.001647684	0.050279548	1.300708147	4.84676238	8.32E-08	2.19E-05	0.139138016	4.84676238	0.56527294	1	0.734248109	4.84676238	0.000621486	0.064339964	purple
Cab39l	0.380719766	6.710905829	0.022581041	0.273951933	0.678451501	6.710905829	1.80E-04	0.009899238	0.062211741	6.710905829	0.731109531	1	0.3599432	6.710905829	0.027073322	0.685465124	purple
Sec14l4	0.534875137	4.018524456	0.038841013	0.3786639	1.041054424	4.018524456	0.000197328	0.010689346	0.055305569	4.018524456	0.844896325	1	0.56148228	4.018524456	0.023780012	0.637906804	purple
Dpep1	0.621248347	7.170932261	0.001841175	0.054633245	1.274829126	7.170932261	4.42E-09	1.81E-06	0.052630899	7.170932261	0.806234209	1	0.706211328	7.170932261	0.000242983	0.031270596	purple
Herpud1	0.324832352	6.644343507	0.045645863	0.413365976	0.736049728	6.644343507	3.09E-05	0.002437658	0.028518044	6.644343507	0.871838229	1	0.439735124	6.644343507	0.005558695	0.286672758	purple
Tmem37	0.312426937	1.610296088	0.359518011	0.875369987	1.194482297	1.610296088	9.42E-04	0.034102749	-0.033814735	1.610296088	0.932250767	1	0.848226096	1.610296088	0.007866059	0.349003314	purple
Ctnnbl1	0.202933088	5.664924001	0.282171458	0.821629834	1.018323042	5.664924001	7.17E-07	0.000130057	-0.077096135	5.664924001	0.707404369	1	0.73829313	5.664924001	5.49E-05	0.009244813	purple
Setdb2	0.303502235	3.643238724	0.204706337	0.751812939	0.993801032	3.643238724	1.20E-04	0.007292034	-0.041942238	3.643238724	0.875568769	1	0.648353749	3.643238724	0.004841626	0.263294013	purple
Setdb2-phf11c	0.301964527	3.78475548	0.186514735	0.730995496	0.887827859	3.78475548	0.000319493	0.015291874	0.015540998	3.78475548	0.953428334	1	0.601401752	3.78475548	0.006331402	0.30702712	purple
Inmt	1.040794509	6.141049989	3.59E-07	7.71E-05	1.945213502	6.141049989	2.67E-18	1.25E-14	0.426854983	6.141049989	0.049833646	0.709014016	1.331272245	6.141049989	6.19E-12	5.41E-09	purple
Elovl3	1.316951643	2.403575347	0.005187665	0.111542773	2.675270648	2.403575347	1.44E-07	3.49E-05	0.723452694	2.403575347	0.144159449	0.994029995	2.081727412	2.403575347	1.28E-06	0.000424439	purple
Bbs9	0.323911685	4.491351099	0.146819264	0.675986263	0.798213091	4.491351099	0.000922544	0.033841126	0.210501736	4.491351099	0.382998327	1	0.684801016	4.491351099	0.001472751	0.121077447	purple
Hccs	0.346707231	4.846496156	0.079466897	0.529953489	0.702549794	4.846496156	9.94E-04	0.035376901	0.284232065	4.846496156	0.18393415	1	0.640072908	4.846496156	0.000820041	0.078216873	purple
Cited1	1.292056572	6.726855209	0.001628993	0.050252623	1.694044408	6.726855209	0.000115448	0.007058465	0.83893387	6.726855209	0.051752528	0.715274536	1.240920204	6.726855209	0.001183105	0.103487941	purple
Ankrd9	1.522982286	3.977521496	4.26E-06	0.000620547	1.810631352	3.977521496	2.06E-07	4.71E-05	1.172880951	3.977521496	0.000765741	0.069493449	1.460515879	3.977521496	1.52E-06	0.000492605	purple
Gk	1.58801393	5.905133288	1.42E-08	4.82E-06	1.947533109	5.905133288	6.36E-11	4.23E-08	1.475250312	5.905133288	6.08E-07	0.000283224	1.834764542	5.905133288	1.61E-12	1.50E-09	purple
Slc4a4	0.733593336	5.659593766	1.93E-04	1.06E-02	0.93213481	5.659593766	1.02E-05	0.001017198	0.697828139	5.659593766	0.000973532	0.082963926	0.896367737	5.659593766	1.95E-06	6.20E-04	purple
Ppif	0.57605741	4.797562573	5.13E-03	0.110805494	0.921523494	4.797562573	3.12E-05	0.002450372	0.523518501	4.797562573	0.018330606	0.458076995	0.868981762	4.797562573	1.07E-05	0.002781713	purple
Lrrc52	1.762604553	-1.31541922	0.073319329	0.515060954	3.232378347	-1.31541922	5.49E-06	0.000661923	1.73324033	-1.31541922	0.094404665	0.883132257	3.20116385	-1.31541922	1.37E-07	5.81E-05	purple
Gmpr	0.396410929	3.302938604	2.40E-01	0.784039429	2.151352508	3.302938604	5.54E-09	2.15E-06	0.29817562	3.302938604	0.411774349	1	2.053104227	3.302938604	1.33E-10	1.03E-07	purple
Cend1	0.421714655	-0.02492599	3.91E-01	0.887695628	1.852359983	-0.02492599	5.95E-05	0.004160542	0.427890975	-0.02492599	0.406397268	1	1.858406803	-0.02492599	3.91E-06	0.001137958	purple
Mmp11	0.053982636	3.301820608	0.886437376	1	1.043283672	3.301820608	0.004029715	0.08939571	0.270766951	3.301820608	0.456598955	1	1.260060926	3.301820608	9.04E-05	0.01404172	purple
Dhrs9	0.046108713	2.087416357	0.914743135	1	1.433609639	2.087416357	0.0003738	0.017246677	0.575875426	2.087416357	0.157307121	1	1.963347329	2.087416357	4.52E-08	2.18E-05	purple
Slc25a34	-0.417462478	1.849186457	0.337796058	0.85956143	1.165976747	1.849186457	0.017100857	0.20397708	-0.042055954	1.849186457	0.932806163	1	1.541366645	1.849186457	0.000384553	0.043575463	yellow
2310050C09Rik	-5.794351339	-1.929092343	0.007231776	0.138538137	5.330516317	-1.929092343	0.004828611	0.10042361	-2.63813539	-1.929092343	0.171000922	1	8.473441346	-1.929092343	2.51E-05	0.005307817	yellow
Awat1	-5.323645551	-2.20239051	0.007170551	0.138038053	8.23511128	-2.20239051	6.84E-05	0.004642806	-5.356447184	-2.20239051	0.03292348	0.60864888	8.204879445	-2.20239051	1.00E-06	0.000359976	yellow
Coch	-1.356107798	0.281665635	0.038879701	0.3786639	3.501929062	0.281665635	1.94E-05	0.001691751	-2.4588584	0.281665635	0.002439642	0.150199913	2.399251744	0.281665635	0.000299486	0.037371556	yellow
Nbl1	-0.23816484	5.919537583	0.155606715	0.68952424	0.826755127	5.919537583	9.63E-06	0.000982322	-0.563691198	5.919537583	0.002492084	0.152093273	0.501228798	5.919537583	0.002739709	0.18380356	yellow
Serpina3j	-1.266276175	0.437321864	9.60E-02	0.574767387	3.912042709	0.437321864	3.95E-05	0.002967507	-2.213768698	0.437321864	0.014340158	0.415805081	2.964597334	0.437321864	0.00011217	0.016501962	yellow
Fa2h	-1.017084897	1.452829135	0.123314418	0.631528879	2.587868571	1.452829135	0.000993772	0.035376901	-1.199558618	1.452829135	0.116587709	0.940779341	2.405384544	1.452829135	0.000326374	0.040012316	yellow
Lce1a2	-5.794998343	-2.436261812	0.004958885	0.108919069	7.801706449	-2.436261812	0.001104974	0.037943768	-5.828029497	-2.436261812	0.026601068	0.549152925	7.771510258	-2.436261812	4.95E-05	0.008875233	yellow
Sdr16c6	-6.299900766	-2.14787901	0.002860027	0.07556093	8.111616558	-2.14787901	0.001191598	0.039835152	-6.333107815	-2.14787901	0.01676538	0.439308492	8.081393817	-2.14787901	5.44E-05	0.009244813	yellow
Gm94	-6.778785664	-1.546906642	0.001840476	0.054633245	8.863536164	-1.546906642	0.000895708	0.033205337	-6.812111423	-1.546906642	0.013226607	0.394989444	8.833268412	-1.546906642	3.20E-05	0.006306695	yellow
Serpina3b	-2.473527938	0.885715834	0.008276685	0.149450842	4.980985466	0.885715834	4.37E-05	0.003197945	-3.122019243	0.885715834	0.005801965	0.24804562	4.332564288	0.885715834	1.12E-05	0.002850493	yellow
Lor	-2.389286944	0.678325548	0.008609371	0.154064748	4.518967916	0.678325548	9.32E-05	0.006022318	-2.981886028	0.678325548	0.007205089	0.28049672	3.926427881	0.678325548	2.87E-05	0.005935889	yellow
Calm4	-4.651982286	-1.245186888	0.002252095	0.063076724	9.1304615	-1.245186888	1.43E-04	0.008286404	-7.322585073	-1.245186888	0.002887989	0.16817725	6.47543838	-1.245186888	2.99E-05	0.006061516	yellow
Krt10	-1.508562462	2.705129684	0.02095764	0.263287053	2.513373311	2.705129684	1.21E-03	0.039899767	-1.537034083	2.705129684	0.041194782	0.664824796	2.484898968	2.705129684	0.00018384	0.024945108	yellow
Krtdap	-3.027264383	0.482229258	0.007303674	0.139448298	4.671525949	0.482229258	0.0009745	0.035139157	-2.918862263	0.482229258	0.027996828	0.563809316	4.779907898	0.482229258	4.70E-05	0.008644648	yellow
Lce1m	-8.049628567	-0.503882042	0.000531914	0.022595817	9.95457059	-0.503882042	7.96E-04	0.030166529	-8.083131232	-0.503882042	0.006216485	0.258376689	9.924267923	-0.503882042	2.50E-05	0.005307817	yellow
Cyp2g1	-8.574935601	-0.292260674	0.000306832	0.014889886	10.08047626	-0.292260674	9.21E-04	0.033841126	-8.608476518	-0.292260674	0.004304853	0.207634596	10.05017101	-0.292260674	3.04E-05	0.00607066	yellow
Crct1	-7.358586001	-1.633763257	0.000672943	0.026948562	8.536012083	-1.633763257	0.001607722	0.048953205	-7.392011713	-1.633763257	0.006887646	0.276162238	8.505761101	-1.633763257	7.45E-05	0.01170174	yellow
Hamp2	-6.15069011	-2.488952939	2.36E-03	0.065329896	5.303878854	-2.488952939	5.77E-03	0.111761879	-3.901813511	-2.488952939	0.048124135	0.69697711	7.541351738	-2.488952939	9.68E-05	0.014706612	yellow
Psapl1	-2.416637443	0.171833608	0.009571305	0.164334842	3.343717987	0.171833608	2.75E-03	0.069040116	-2.068632711	0.171833608	0.054157533	0.73130984	3.6916733	0.171833608	9.88E-05	0.014840599	yellow
Rnase2b	-6.158255644	-2.338810684	1.09E-03	0.037793345	4.635965597	-2.338810684	3.49E-03	0.080707258	-3.009584835	-2.338810684	0.065357153	0.779378476	7.771541056	-2.338810684	1.07E-05	0.002781713	yellow
Dmkn	-2.182662497	1.898978278	0.010372337	0.173609322	3.12514811	1.898978278	2.34E-03	0.062527393	-1.726164455	1.898978278	0.078699481	0.829490156	3.581627246	1.898978278	5.12E-05	0.008936576	yellow
Spink5	-2.945771851	0.196011606	4.78E-03	1.06E-01	2.739134472	0.196011606	2.22E-02	2.34E-01	-1.836579972	0.196011606	0.119358663	0.946835239	3.848216355	0.196011606	0.000278866	0.035112051	yellow
Lce1a1	-4.541348911	-2.418568698	1.38E-03	0.044959173	3.039643097	-2.418568698	1.96E-02	2.18E-01	-2.022111802	-2.418568698	0.134910309	0.983974457	5.555482801	-2.418568698	5.10E-05	0.008936576	yellow
Defb6	-7.939927477	-1.199066988	0.000691753	0.027622678	4.360326483	-1.199066988	3.23E-02	0.289603338	-3.415169998	-1.199066988	0.101456556	0.901434724	8.87097783	-1.199066988	0.000154598	0.021392746	yellow
Cyp26b1	-0.929659331	3.58954082	0.000720425	0.027968495	0.811934894	3.58954082	0.008411176	0.138953415	-0.793167629	3.58954082	0.010024521	0.340055107	0.948426475	3.58954082	0.000561161	0.058968362	yellow
Mafb	-0.761999625	6.156003759	0.001105917	0.037976173	0.436041343	6.156003759	9.45E-02	0.498074045	-0.96402004	6.156003759	0.000237831	0.031357795	0.234021394	6.156003759	0.316722981	1	yellow
Il33	-2.277792588	4.437293273	5.81E-06	0.000788775	1.30309815	4.437293273	0.02123273	0.227742623	-2.160945947	4.437293273	0.000191893	0.027648483	1.419946304	4.437293273	0.004241066	0.244930341	yellow
Nuak2	-0.73203141	3.325934643	0.001537898	0.04851842	0.175510542	3.325934643	4.99E-01	0.890090934	-0.552093749	3.325934643	0.031967361	0.604471554	0.355449563	3.325934643	0.126736189	1	yellow
Adrb2	-0.73094708	3.954353775	0.0008949	0.032498532	0.158617771	3.954353775	0.519715207	0.898174816	-0.486178069	3.954353775	0.047196947	0.692882916	0.403387383	3.954353775	0.068177817	1	yellow
Chil1	-1.12320778	4.345506428	0.001092994	0.037811095	0.402341579	4.345506428	0.295259667	0.765358313	-0.535394815	4.345506428	0.16388465	1	0.990153967	4.345506428	0.004003491	0.234112097	yellow
Tmem132c	-1.260047674	2.776117532	5.72E-05	0.004185854	0.191467457	2.776117532	0.586825478	0.919099782	-0.515255267	2.776117532	0.13879472	0.990196533	0.936258508	2.776117532	0.00288177	0.189641677	yellow
Aspg	-2.282092526	4.194521625	1.44E-12	1.06E-09	-0.900980146	4.194521625	1.07E-02	0.156648581	0.903413653	4.194521625	0.010492992	0.34669045	2.284517927	4.194521625	1.42E-12	1.42E-09	royalblue
A330041J22Rik	-1.257119042	5.530070629	2.88E-04	0.014240943	-0.56758781	5.530070629	1.43E-01	0.592754364	0.595548439	5.530070629	0.124415558	0.956226206	1.285078347	5.530070629	0.000210857	0.027801341	royalblue
Me1	-1.219404243	10.62586096	0.00095579	0.033990115	-0.542131807	10.62586096	1.90E-01	0.661557439	0.646588245	10.62586096	0.118480975	0.945142756	1.323860639	10.62586096	0.00033837	0.040767691	royalblue
Sorcs2	-0.991711004	4.126960226	8.66E-05	0.005933737	-0.724327254	4.126960226	0.009704436	0.150487956	0.702423807	4.126960226	0.012108277	0.379428874	0.969804711	4.126960226	0.0001247	0.017967041	royalblue

Slc2a5	-2.04260938	4.249812902	2.37E-05	0.00208639	-1.188271126	4.249812902	0.027736624	0.264064748	1.346040678	4.249812902	0.012998977	0.391208682	2.200370336	4.249812902	5.53E-06	0.001544725	royalblue
Pygl	-0.872403081	8.096352134	0.000416617	0.01860268	-0.479860021	8.096352134	0.081801974	0.465541383	0.612003335	8.096352134	0.026665115	0.549663193	1.004546211	8.096352134	4.81E-05	0.008722537	royalblue
Pdk4	-0.715150669	8.515306032	5.87E-04	0.024565329	-0.230199632	8.515306032	3.20E-01	0.787640039	0.411205913	8.515306032	0.075762605	0.817704713	0.896156837	8.515306032	1.62E-05	0.003783626	royalblue
Adcy10	-1.536128352	1.987654346	1.92E-04	0.010600388	-0.581208879	1.987654346	1.92E-01	0.662606457	1.066381587	1.987654346	0.017802601	0.448304783	2.021269276	1.987654346	9.08E-07	0.000333845	royalblue
Gpd2	-0.634755852	7.670563161	0.00640689	0.129772028	-0.347269572	7.670563161	0.179968762	0.6467584	0.727564945	7.670563161	0.00514686	0.235844335	1.015050935	7.670563161	1.27E-05	0.003001013	royalblue
Apold1	-0.573535562	7.052820663	0.002893156	0.07629197	-0.267153606	7.052820663	0.210717502	0.687438797	0.732720092	7.052820663	0.000634394	0.062002007	1.039101582	7.052820663	6.23E-08	2.81E-05	royalblue
Adprhl1	-2.202218723	-0.901186619	1.20E-02	0.190158556	-0.334253868	-0.901186619	0.670084635	0.951254734	2.079579028	-0.901186619	0.014752927	0.419932609	3.946694221	-0.901186619	2.18E-06	0.000675596	royalblue
Otop1	-0.491609819	3.974809309	2.81E-01	0.821408556	-0.577841468	3.974809309	0.25529048	0.729851304	2.091584708	3.974809309	7.39E-05	0.013772289	2.00534065	3.974809309	1.20E-05	0.002951736	royalblue
Acot11	-0.368704185	4.251496329	3.13E-01	0.844786178	-0.256155914	4.251496329	0.524383625	0.900170012	2.518993281	4.251496329	3.55E-09	3.10E-06	2.63152609	4.251496329	1.17E-12	1.26E-09	royalblue
Fam151a	-0.475972696	2.443826775	2.86E-01	0.823769409	-0.185897063	2.443826775	0.69787893	0.955882348	2.416175936	2.443826775	1.91E-06	0.000797648	2.706198354	2.443826775	1.08E-09	6.59E-07	royalblue
Dcpp3	-1.980876233	-1.5562302	0.127098347	0.63968133	-0.245606192	-1.5562302	0.682153701	0.952820984	3.519379088	-1.5562302	5.66E-07	0.000282839	5.251161091	-1.5562302	1.85E-11	1.52E-08	royalblue
Cox8b	-0.28928321	5.746735495	2.07E-01	0.753949176	-0.010348918	5.746735495	9.68E-01	1	1.046538191	5.746735495	4.35E-05	0.009384939	1.3254707	5.746735495	6.03E-09	3.24E-06	royalblue
Cidea	-0.097251067	5.986852101	0.689350678	0.97543435	0.280271529	5.986852101	0.300545638	0.770624892	2.129799683	5.986852101	4.54E-14	9.06E-11	2.507318234	5.986852101	4.14E-24	9.65E-21	royalblue
Letmd1	-0.127861161	6.222308186	0.511362703	0.934711632	0.085498934	6.222308186	0.692826441	0.955447289	0.616907903	6.222308186	0.004467841	0.213044717	0.830267229	6.222308186	1.97E-05	0.004300966	royalblue
Gnao1	-0.250625174	3.434116951	0.276980844	0.818930459	0.196042178	3.434116951	0.437087254	0.863970822	0.550385415	3.434116951	0.029792243	0.579250129	0.997047175	3.434116951	1.15E-05	0.002861715	royalblue
Pim1	-0.231502722	6.042434314	0.254409486	0.797836601	0.059071063	6.042434314	7.94E-01	0.976199887	0.431643507	6.042434314	0.055727828	0.742471039	0.722216637	6.042434314	0.000365708	0.042950711	royalblue
Mtfp1	-0.376855756	3.089599219	0.174805337	0.719187341	0.207293322	3.089599219	4.98E-01	0.889724687	0.544428964	3.089599219	0.076301912	0.818134822	1.128570524	3.089599219	4.08E-05	0.007700671	royalblue
Gm9899	-0.641662406	3.896457329	0.022649869	0.274106207	0.425307368	3.896457329	0.17028881	0.634485847	0.703854957	3.896457329	0.024125605	0.52932411	1.770818102	3.896457329	2.54E-10	1.77E-07	royalblue
Pvalb	-1.624705605	0.280747195	0.068466628	0.501694422	0.357783774	0.280747195	0.713997787	0.961722539	1.360446743	0.280747195	0.168055548	1	3.342723504	0.280747195	0.000220105	0.028749356	royalblue
Atp2a1	-1.328473241	1.318057273	0.002945789	0.076810355	0.217378569	1.318057273	6.51E-01	0.942302597	0.69856003	1.318057273	0.151228804	0.999246012	2.244363116	1.318057273	4.14E-07	0.000160712	royalblue
Ckm	-1.650232504	1.253371109	0.017807714	0.237247232	0.339324435	1.253371109	6.57E-01	0.945507563	1.056395075	1.253371109	0.1725831	1	3.045866803	1.253371109	1.68E-05	0.003803256	royalblue
Ucp3	-0.551562936	5.052887525	0.003924999	0.093292153	0.055722694	5.052887525	7.93E-01	0.976199887	0.425521964	5.052887525	0.044071427	0.679711223	1.032806042	5.052887525	5.64E-08	2.63E-05	royalblue
Kcnh1	-0.884099701	4.357395023	0.002155606	0.061483175	-0.003102401	4.357395023	9.94E-01	1	0.742926335	4.357395023	0.020589472	0.483627674	1.623919306	4.357395023	1.68E-08	8.68E-06	royalblue
Tnnt3	-1.365226097	1.107875076	0.010278167	0.172861206	0.520316974	1.107875076	3.66E-01	0.82073697	0.591495873	1.107875076	0.309808698	1	2.47697788	1.107875076	3.12E-06	0.000927604	royalblue
Tnni2	-0.990455147	-0.438529831	0.062208858	0.476415264	0.387333417	-0.438529831	4.78E-01	0.882522979	0.414507557	-0.438529831	0.482774674	1	1.792177061	-0.438529831	0.000371333	0.043247899	royalblue
Fam69b	-0.461431028	3.166520478	0.077086497	0.52413909	0.188496775	3.166520478	5.14E-01	0.894229	0.301630386	3.166520478	0.296352299	1	0.951553152	3.166520478	0.000243882	0.031270596	royalblue
Acacb	-0.429035816	8.353633843	0.086886502	0.548432133	0.378185889	8.353633843	0.175383931	0.641834466	0.295704593	8.353633843	0.289165412	1	1.102926137	8.353633843	1.04E-05	0.002781713	royalblue
Kcnk3	-0.435779831	6.662795487	0.079051707	0.528372382	0.593253815	6.662795487	0.031830997	0.286827864	0.502706056	6.662795487	0.068874007	0.795523236	1.5317389	6.662795487	6.44E-10	4.29E-07	royalblue
Pamr1	-0.246043608	4.855085314	0.393984776	0.889563201	0.861156352	4.855085314	0.007754156	0.133633206	0.422529335	4.855085314	0.190803306	1	1.529726467	4.855085314	1.20E-07	5.22E-05	royalblue
Abi3	-0.066395634	5.852880377	0.694765024	0.975980943	0.852408628	5.852880377	5.42E-06	0.00065829	0.869119253	5.852880377	4.00E-06	0.001362649	1.78792141	5.852880377	2.56E-26	7.17E-23	royalblue
Phospho1	-0.1343002	5.640943926	0.455215821	0.913501714	0.972694992	5.640943926	8.73E-07	0.000150677	1.125593689	5.640943926	1.73E-08	1.27E-05	2.232585606	5.640943926	7.83E-36	5.47E-32	royalblue
Perm1	-0.125559782	2.262536387	0.6967799	0.976014878	0.763772629	2.262536387	2.28E-02	0.236750324	0.925736952	2.262536387	0.006915912	0.276162238	1.815043349	2.262536387	2.47E-09	1.38E-06	royalblue
Hc	-0.104555078	5.530403666	0.737123023	0.981536925	0.395420179	5.530403666	0.268922609	0.74085702	0.636309181	5.530403666	0.076326786	0.818134822	1.136282834	5.530403666	0.000386617	0.043575463	royalblue
Pank1	-0.022068755	4.739562782	0.907632539	1	0.613658868	4.739562782	4.76E-03	0.100263094	1.193639047	4.739562782	6.76E-08	3.78E-05	1.829361352	4.739562782	1.21E-20	2.12E-17	royalblue
A530050N04Rik	1.156883735	0.639816728	0.498960173	0.932175597	1.979492105	0.639816728	1.35E-05	0.001281951	5.988632528	0.639816728	4.00E-15	9.32E-12	6.806813526	0.639816728	6.08E-35	2.83E-31	royalblue
Dagla	0.12793813	4.196815496	0.525356544	0.938266133	0.869844278	4.196815496	4.31E-05	0.00316872	0.713201075	4.196815496	0.000907432	0.078771833	1.455101685	4.196815496	2.43E-14	2.83E-11	royalblue
Slc27a2	0.832785755	3.229791259	0.006714714	0.133302339	1.895757658	3.229791259	3.08E-10	1.79E-07	2.601784263	3.229791259	1.54E-15	5.39E-12	3.664686727	3.229791259	3.24E-39	4.53E-35	royalblue
Cldn1	0.353060772	3.499178984	0.240777251	0.78435695	0.709409434	3.499178984	0.026145129	0.255153509	0.796537704	3.499178984	0.013640528	0.402194133	1.152877788	3.499178984	5.43E-05	0.009244813	royalblue
Ldhb	0.438268791	6.283421373	0.025731213	0.296349735	0.381942705	6.283421373	0.071173198	0.43743035	0.743301164	6.283421373	0.000480212	0.050844272	0.68697414	6.283421373	0.000307498	0.038031772	royalblue
Fabp3	1.649205304	2.486591348	0.007315176	0.139477362	1.426686496	2.486591348	0.010919367	0.158773827	4.607004825	2.486591348	5.37E-12	7.50E-09	4.384146026	2.486591348	4.85E-16	6.16E-13	royalblue
Acss1	0.129226847	4.513668016	0.52596481	0.938266133	0.227405615	4.513668016	0.294769221	0.765202649	0.600555807	4.513668016	0.00601094	0.254083859	0.698732052	4.513668016	0.000358319	0.042802318	royalblue
Ppara	0.145557457	5.176667183	0.515290526	0.936855884	0.247312906	5.176667183	0.298657406	0.769765915	1.198400063	5.176667183	7.09E-07	0.000319836	1.300152333	5.176667183	1.27E-09	7.37E-07	royalblue
Gm44502	0.221703812	6.238748628	0.228560175	0.773020276	0.287289243	6.238748628	0.145691319	0.596927386	1.600815752	6.238748628	2.78E-15	7.77E-12	1.666399023	6.238748628	8.81E-21	1.76E-17	royalblue
Cox7a1	0.15156033	3.106297835	0.605018471	0.961467803	0.247837601	3.106297835	0.390763971	0.838396877	2.191484315	3.106297835	9.39E-13	1.46E-09	2.287732058	3.106297835	8.63E-18	1.21E-14	royalblue
Acaa2	0.165856503	7.189805404	0.335142476	0.858883664	0.130598465	7.189805404	4.83E-01	0.884299667	0.721128851	7.189805404	0.000114666	0.018965291	0.685870385	7.189805404	4.24E-05	0.007893084	royalblue
Ppp1r3b	0.37576778	5.353351514	0.048905421	0.425171207	0.219918957	5.353351514	0.278218699	0.750074179	1.787109551	5.353351514	1.61E-17	7.51E-14	1.631256332	5.353351514	5.91E-19	9.18E-16	royalblue
Cpt1b	0.942238284	5.387950268	0.000204372	0.010942234	0.496792911	5.387950268	5.84E-02	0.395362461	3.332175146	5.387950268	1.18E-30	1.65E-26	2.886716335	5.387950268	9.69E-33	3.39E-29	royalblue
Hsd11b1	0.376785234	6.259639053	0.085813279	0.545075371	0.11886881	6.259639053	0.615059738	0.928502365	0.870799276	6.259639053	0.000253543	0.031964298	0.612881916	6.259639053	0.003931403	0.231836632	royalblue
Nr4a3	0.492119494	6.886749523	0.027798312	0.308229671	0.014115945	6.886749523	9.53E-01	1	1.02871201	6.886749523	2.30E-05	0.005839258	0.550707792	6.886749523	0.010930211	0.420828182	royalblue
Coq10b	0.27743915	6.188785492	0.090950314	0.559037814	-0.022064126	6.188785492	9.02E-01	0.997031331	0.724293667	6.188785492	4.86E-05	0.00998344	0.424789666	6.188785492	0.007977379	0.349504208	royalblue
Sbp	0.658855129	1.319840146	0.492477561	0.930368531	-0.415206267	1.319840146	0.656709584	0.945507563	3.664981385	1.319840146	0.000423995	0.046381842	2.590706364	1.319840146	0.002185895	0.160789842	royalblue
Gys2	0.292309719	4.952167384	0.395593517	0.889930544	-0.222971192	4.952167384	0.54269308	0.904681263	1.382706723	4.952167384	0.000209949	0.029051924	0.867422472	4.952167384	0.008236913	0.353126047	royalblue
Nr4a2	0.248740362	5.853623534	0.30577194	0.837935027	-0.215567236	5.853623534	4.09E-01	0.849836988	1.016162064	5.853623534	0.000115344	0.018965291	0.551853274	5.853623534	0.018727424	0.559261714	royalblue
Gm5148	0.484067329	2.364143918	0.123706215	0.632145542	-0.442237018	2.364143918	1.76E-01	6.43E-01	1.445360428	2.364143918	1.69E-05	0.004646006	0.519037756	2.364143918	0.079819144	1	royalblue
Clic5	0.152535707	5.662526399	0.451907513	0.913501714	-0.410719812	5.662526399	0.059867676	0.400346125	0.860208754	5.662526399	8.95E-05	0.015442991	0.296952327	5.662526399	0.131754723	1	lightgreen
Csn1s1	0.443217905	1.59340209	0.460792888	0.915038563	-1.460958841	1.59340209	1.59E-02	0.196111641	3.064660193	1.59340209	2.25E-06	0.000872703	1.160399411	1.59340209	0.031877922	0.733837288	lightgreen
Pnldc1	0.279002982	1.782950037	0.43757327	0.908732183	-0.760837746	1.782950037	3.90E-02	0.322005228	1.348538436	1.782950037	0.000316363	0.036845692	0.308677765	1.782950037	0.355627924	1	lightgreen
Tnfsf11	0.957359352	0.401056271	0.376076358	0.884817534	-2.627814473	0.401056271	0.010996292	0.159359628	4.29980646	0.401056271	0.000133635	0.02098527	0.714284652	0.401056271	0.40233657	1	lightgreen
Bdnf	0.429207257	4.155867806	0.054650741	0.445363704	-0.556035349	4.155867806	2.02E-02	2.22E-01	1.092593643	4.155867806	6.25E-06	0.001986106	0.107348032	4.155867806	0.618126061	1	lightgreen
Wnk4	0.594721784	2.861576326	0.046978526	0.41987895	-0.440107431	2.861576326	1.66E-01	0.627233726	1.1971702	2.861576326	0.000207095	0.028943588	0.162331602	2.861576326	0.568036083	1	lightgreen
Csn2	2.971016679	0.035740291	0.000278336	0.01384349	-1.41176907	0.035740291	0.038704192	0.320645987	4.978883795	0.035740291	4.67E-09	3.84E-06	0.595117135	0.035740291	0.331140929	1	lightgreen
Defb15	2.06313401	-2.386333859	0.045098365	0.410348141	-2.199581646	-2.386333859	0.004821588	0.10042361	3.582984864	-2.386333859	0.000104995	0.017679668	-0.681563897	-2.386333859	0.45136269	1	lightgreen
Fcgbp	0.326509018	6.663183916	0.34449672	0.864240918	-1.322640492	6.663183916	0.000423086	0.01865317	1.513813523	6.663183916	5.95E-05	0.011882753	-0.135336437	6.663183916	0.70050342	1	lightgreen
Atp7b	0.039253765	0.856377626	1	1	-3.745261218	0.856377626	5.96E-13	6.21E-10	3.900651099	0.856377626	9.63E-14	1.68E-10	0.116026863	0.856377626	0.857216099	1	lightgreen
Entpd8	1.542325885	-0.82659896	0.317351878	0.846885321	-4.797700208	-0.82659896	0.000597215	0.024621483	5.585745661	-0.82659896	0.00015503	0.023050036	-0.755482219	-0.82659896	0.638498775	1	lightgreen
Calca	0.220416357	1.603758299	0.835135454	0.994187369	-3.398801094	1.603758299	7.76E-04	0.029626726	3.209511908	1.603758299	0.001373933	0.103101487	-0.409727725	1.603758299	0.672448517	1	lightgreen
Ppp1r3d	0.176353574	3.243076303	0.502770212	0.933318504	-1.355691633	3.243076303	1.64E-06	0.000249252	1.148886836	3.243076303	4.31E-05	0.009384939	-0.383159489	3.243076303	0.14263453	1	lightgreen
Gm4847	0.045343401	0.39777344	1	1	-6.113654613	0.39777344	2.56E-05	0.002099505	3.002351521	0.39777344	0.014272225	0.414695662	-3.155662777	0.39777344	0.016052488	0.514563239	lightgreen
Il1a	-0.343717644	1.581689886	0.479285448	0.924567759	-2.145412503	1.581689886	0.000151321	0.008603816	1.632698095	1.581689886	0.00326584	0.177600705	-0.16900016	1.581689886	0.761596301	1	lightgreen
Rasef	-0.328868077	5.213830757	0.16922153	0.709710104	-1.188423828	5.213830757	9.75E-06	0.000987831	0.826356725	5.213830757	0.001946253	0.129527749	-0.033199227	5.213830757	0.901517151	1	lightgreen
Il12b	-0.54795453	0.308394811	0.337751791	0.85956143	-2.256251544	0.308394811	0.000463363	0.019926014	1.794597594	0.308394811	0.004611464	0.217735883	0.086277809	0.308394811	0.911147304	1	lightgreen
Sc5d	-0.115604283	6.498008096	0.538404209	0.94090594	-0.70728118	6.498008096	0.000747172	0.028926523	0.650615648	6.498008096	0.001907872	0.127580947	0.058938561	6.498008096	0.751308701	1	lightgreen
Muc15	-0.207339539	5.170642329	0.509681051	0.934141884	-1.161973227	5.170642329	0.001142747	0.038670775	1.315099458	5.170642329	0.00024407	0.031790079	0.360464358	5.170642329	0.254478406	1	lightgreen
Gm4841	-0.799535061	1.474439892	0.045698701	0.413388376	-1.705943976	1.474439892	0.00012736	0.00767234	1.609558256	1.474439892	0.000281477	0.034813489	0.703125351	1.474439892	0.082189267	1	lightgreen
Mir7116	-1.465155389	0.022203241	0.01222525	0.192019475	-1.991528172	0.022203241	0.001473531	0.046039414	1.563440976	0.022203241	0.011274531	0.363337878	1.03698843	0.022203241	0.080762088	1	orange
Iigp1	-0.872827283	7.979361033	0.000191994	0.010600388	-1.102543859	7.979361033	2.85E-05	0.002303482	0.855846022	7.979361033	0.00109893	0.089742492	0.626129306	7.979361033	0.007532802	0.338515871	orange
Slc25a35	-0.724672329	5.861236418	0.000359515	0.016804611	-0.758768853	5.861236418	8.02E-04	0.030291008	0.508696588	5.861236418	0.024112504	0.52932411	0.474599706	5.861236418	0.019763405	0.577852195	orange
Ppp1r9a	-0.53148292	5.006422573	0.001717669	0.051961348	-0.667063963	5.006422573	0.000388809	0.017585738	0.356372451	5.006422573	0.057002695	0.746059679	0.220791185	5.006422573	0.194842762	1	orange
Rnf150	-0.571685017	4.480421577	0.00137737	0.044959173	-0.715912844	4.480421577	0.000301869	0.014649038	0.380427403	4.480421577	0.053551521	0.727343113	0.236199239	4.480421577	0.188841473	1	orange
Hspa4l	-0.469893335	4.484511816	0.010054391	0.170327473	-0.636194714	4.484511816	0.001645134	0.049767086	0.345008987	4.484511816	0.08642649	0.849375486	0.178707334	4.484511816	0.329896093	1	orange
Adap1	-0.680845788	4.184863696	0.011725827	0.188367998	-0.965989101	4.184863696	0.001387335	0.044066796	0.556349298	4.184863696	0.063733328	0.77496143	0.271205403	4.184863696	0.316828497	1	orange
Cxcl9	-0.390900805	6.373669034	0.057172263	0.45557183	-0.757509217	6.373669034	0.000939887	0.034102749	0.554009303	6.373669034	0.015252968	0.426259774	0.187400725	6.373669034	0.361291757	1	orange
Mir6935	-0.694302189	1.723946894	0.071199197	0.509252803	-1.371336578	1.723946894	0.001506664	0.046381353	0.911697789	1.723946894	0.033031823	0.609337417	0.23465877	1.723946894	0.548523075	1	orange
Rhoq	-0.326156821	7.217831862	0.074114664	0.516364178	-0.757403529	7.217831862	0.000195149	0.010612473	0.48562889	7.217831862	0.016533782	0.439241368	0.054382141	7.217831862	0.762188183	1	orange
Hvcn1	-0.354258989	4.794303331	0.055287285	0.448608979	-0.752088966	4.794303331	2.43E-04	0.012463372	0.447569544	4.794303331	0.028216539	0.564152758	0.049739418	4.794303331	0.785963075	1	orange
Pigf	-0.353216476	4.639523102	0.051458136	0.434024692	-0.735852136	4.639523102	0.000249595	0.01273117	0.441141419	4.639523102	0.027379018	0.557797606	0.058505577	4.639523102	0.746155676	1	orange
Zfp667	-0.560713191	3.799779915	0.005902156	0.122735018	-1.117576751	3.799779915	8.22E-07	0.000145358	0.618719872	3.799779915	0.00577291	0.24804562	0.061856191	3.799779915	0.761342718	1	orange
Palm2	-0.57134873	2.774626089	0.035356118	0.357048393	-1.031307825	2.774626089	0.000660288	0.026366243	0.573936841	2.774626089	0.054633185	0.732627562	0.113977153	2.774626089	0.678502794	1	orange
Tmem179	-0.558965823	5.965973401	0.007806078	0.144500326	-0.99052897	5.965973401	2.51E-05	0.002099505	0.586772427	5.965973401	0.011981225	0.378845246	0.155209162	5.965973401	0.459909137	1	orange
Myo5c	-0.786344777	3.966273098	0.002655801	0.071793962	-1.114909745	3.966273098	0.000146325	0.00845058	0.51743741	3.966273098	0.074810892	0.813663053	0.188872363	3.966273098	0.471415561	1	orange
Snhg11	-0.919016947	5.103249215	0.007457875	0.140662966	-1.303494881	5.103249215	0.000819413	0.03078525	0.560356046	5.103249215	0.144852754	0.994029995	0.175878201	5.103249215	0.604048145	1	orange
Ssbp3	-0.407537738	5.836069193	0.009234981	0.16025376	-0.620473732	5.836069193	0.000353276	0.016624199	0.281159254	5.836069193	0.104183903	0.907211355	0.068223261	5.836069193	0.662032072	1	orange
Mob3b	-0.447892072	3.969163962	0.027756986	0.308229671	-0.760908036	3.969163962	0.000759124	0.029147023	0.358094515	3.969163962	0.110937219	0.921880751	0.045078612	3.969163962	0.823127278	1	orange
Neat1	-0.529793435	6.869479887	0.035008396	0.355195794	-0.912875549	6.869479887	0.001194435	0.039835152	0.398962519	6.869479887	0.153981822	1	0.015880444	6.869479887	0.941462947	1	orange
Ssx2ip	-0.461745481	4.805006289	0.015908104	0.22255422	-0.864540524	4.805006289	5.08E-05	0.003607063	0.386382517	4.805006289	0.068145915	0.79192982	-0.016412356	4.805006289	0.942745965	1	orange
Scd2	-0.781105085	8.109843498	0.017865963	0.237352372	-1.419896191	8.109843498	0.000155658	0.008772065	0.589731565	8.109843498	0.110519412	0.920939068	-0.049059488	8.109843498	0.896422375	1	orange
P2rx5	-1.062834767	2.880286578	0.000648568	0.02642919	-1.989519147	2.880286578	2.68E-08	7.97E-06	0.818043255	2.880286578	0.017533177	0.447803905	-0.108637072	2.880286578	0.75142543	1	orange
Mettl24	-0.633187847	1.332498869	0.065006201	0.485583463	-1.247340448	1.332498869	0.001197107	0.039835152	0.519698285	1.332498869	0.16985187	1	-0.094448846	1.332498869	0.803846842	1	orange
Scnn1g	-2.003851461	1.220678968	2.30E-05	0.002058739	-3.359141801	1.220678968	3.15E-09	1.42E-06	1.097082057	1.220678968	0.031963597	0.604471554	-0.258154805	1.220678968	0.636200751	1	orange
Mir5114	-0.969978095	3.204860557	0.01106503	0.18065989	-1.606616144	3.204860557	0.000230556	0.011978651	0.566621317	3.204860557	0.183218096	1	-0.070014113	3.204860557	0.87454932	1	orange
Ptch2	-1.034472066	3.678179143	0.001015244	0.035472641	-1.601307658	3.678179143	8.43E-06	0.00090111	0.388329907	3.678179143	0.267580478	1	-0.178502728	3.678179143	0.59295623	1	orange
Ptger3	-0.993146098	5.644269522	1.12E-05	0.001258001	-1.366365435	5.644269522	9.24E-08	2.39E-05	0.300925939	5.644269522	0.231130818	1	-0.072292837	5.644269522	0.762804468	1	orange
Acp6	-0.489637577	4.895307338	0.003139081	0.080058024	-0.67040226	4.895307338	0.000272301	0.013543364	0.158814141	4.895307338	0.38602694	1	-0.021950188	4.895307338	0.904257009	1	orange
Thrb	-0.669917768	4.446811916	0.000206052	0.010942234	-0.895472029	4.446811916	8.65E-06	0.000903978	0.220266545	4.446811916	0.269147933	1	-0.00528709	4.446811916	0.986509046	1	orange
Gbp7	-0.457904515	5.759103308	0.013070242	0.199421077	-0.674189966	5.759103308	0.001022491	0.036088689	0.202955569	5.759103308	0.320427511	1	-0.01332973	5.759103308	0.951158193	1	orange
A530016L24Rik	-0.702238455	7.580158747	0.002078308	0.059889562	-1.0714495	7.580158747	2.94E-05	0.002348845	0.366432653	7.580158747	0.148947808	0.995387977	-0.002778329	7.580158747	0.999155672	1	orange
Dhcr7	-0.458623782	4.515825698	0.019747333	0.253899471	-0.714359178	4.515825698	0.001087907	0.037542199	0.245659429	4.515825698	0.25863291	1	-0.010075658	4.515825698	0.968737684	1	orange
Rb1	-0.399098568	6.396382164	0.013780459	0.204237212	-0.590198756	6.396382164	0.001034118	0.036405126	0.210679531	6.396382164	0.239888737	1	0.019579394	6.396382164	0.899932071	1	orange
Trps1	-0.545990343	7.387082765	0.006241603	0.127533108	-0.846600336	7.387082765	0.000148185	0.00848783	0.339192441	7.387082765	0.12616178	0.960892121	0.038582477	7.387082765	0.841533808	1	orange
Ttc28	-0.459165051	6.488178468	0.007449915	0.140662966	-0.663351878	6.488178468	0.000508444	0.021598835	0.258697514	6.488178468	0.173464623	1	0.05451072	6.488178468	0.748042031	1	orange
Kcnb1	-0.892706175	5.431611756	7.57E-06	0.000962194	-1.247255716	5.431611756	2.74E-08	7.97E-06	0.443883163	5.431611756	0.044781018	0.680286059	0.089333862	5.431611756	0.654747751	1	orange
Cc2d2a	-0.680553423	4.695990531	0.001209889	0.040745561	-0.856052502	4.695990531	0.000267678	0.013408856	0.222258663	4.695990531	0.340413139	1	0.046759991	4.695990531	0.821402869	1	orange
Igtp	-0.670373849	5.433590422	0.000635473	0.026045092	-0.873345725	5.433590422	6.79E-05	0.00463165	0.273077815	5.433590422	0.209291883	1	0.070106119	5.433590422	0.719097819	1	orange
Mlxipl	-1.03148438	6.801061989	0.000332403	0.015801578	-1.277558618	6.801061989	8.64E-05	0.005641246	0.379357133	6.801061989	0.236895584	1	0.133283008	6.801061989	0.638588478	1	orange
Sema6d	-0.614565563	5.687837295	0.000875388	0.032027281	-0.62050477	5.687837295	0.0025362	0.065519286	0.184632452	5.687837295	0.367322124	1	0.178693272	5.687837295	0.334668979	1	orange
Tbc1d32	-0.591500442	4.153392303	0.001284544	0.042846734	-0.545944329	4.153392303	0.007360496	0.129722935	0.136282095	4.153392303	0.501992866	1	0.181838303	4.153392303	0.326212062	1	orange

Rybp	-0.639841447	5.436805808	0.0005725	0.024027816	-0.535200483	5.436805808	0.009607662	0.150206382	0.080761357	5.436805808	0.695717387	1	0.185402413	5.436805808	0.319956288	1	orange
Cxadr	-0.879892619	6.191492394	1.38E-05	0.001490146	-0.72270654	6.191492394	0.001393159	0.044110588	0.087761621	6.191492394	0.698110965	1	0.244557784	6.191492394	0.228714812	1	orange
Cdon	-0.745959547	6.152157737	0.000181507	0.010516358	-0.684982553	6.152157737	0.002067559	0.058118807	0.110654598	6.152157737	0.617365135	1	0.171631687	6.152157737	0.390027274	1	orange
Tspan18	-0.827130995	6.399945745	1.54E-05	0.001579378	-0.754602441	6.399945745	0.00041346	0.018344482	0.128349896	6.399945745	0.545733599	1	0.200878529	6.399945745	0.295701712	1	orange
Mapkapk3	-0.937737005	4.565284086	8.99E-06	0.001091972	-0.852567263	4.565284086	0.000305581	0.014777865	0.118435192	4.565284086	0.614158795	1	0.203605336	4.565284086	0.339012737	1	orange
Gbp5	-0.85523878	4.427991697	0.000292389	0.014388834	-0.905662384	4.427991697	0.000613688	0.02500553	0.196158809	4.427991697	0.454332859	1	0.145735651	4.427991697	0.537956656	1	orange
Rdh10	-0.685188021	4.368808025	0.000173101	0.010122417	-0.739385584	4.368808025	0.000271159	0.013534716	0.154027213	4.368808025	0.445408626	1	0.099830068	4.368808025	0.58734793	1	orange
N4bp2l1	-0.671119694	4.94532962	0.000977825	0.034597687	-0.700540921	4.94532962	0.002023438	0.057301809	0.135060256	4.94532962	0.54961514	1	0.1056393	4.94532962	0.603525418	1	orange
Cntnap1	-1.193910129	4.241899136	1.55E-07	3.80E-05	-1.231000312	4.241899136	1.54E-06	0.000239423	0.187490101	4.241899136	0.457157446	1	0.150400933	4.241899136	0.513422745	1	orange
Sfxn5	-0.837619268	4.622528233	0.000305646	0.014884004	-0.893653754	4.622528233	0.000581752	0.024126307	0.102047001	4.622528233	0.69270286	1	0.046013221	4.622528233	0.840103181	1	orange
Syne1	-0.66081935	6.307088667	1.04E-05	0.001210173	-0.666939013	6.307088667	6.46E-05	0.004450547	0.049646995	6.307088667	0.765359477	1	0.043527504	6.307088667	0.769808792	1	orange
Tbc1d31	-0.84206528	4.040143096	5.79E-05	0.004216646	-0.836806086	4.040143096	0.000354992	0.016648873	0.005050155	4.040143096	0.984612541	1	0.010310665	4.040143096	0.956610407	1	orange
Meis2	-0.700058396	3.874778263	0.000446017	0.019726362	-0.714505701	3.874778263	0.001317333	0.042130548	0.007279492	3.874778263	0.976497516	1	-0.007166536	3.874778263	0.983658995	1	orange
Ccdc69	-1.086583828	4.017663834	3.26E-08	9.29E-06	-1.163196229	4.017663834	1.45E-07	3.49E-05	0.01959087	4.017663834	0.930223988	1	-0.057019449	4.017663834	0.788924974	1	orange
Vgll4	-0.775667814	5.362427678	4.90E-07	9.79E-05	-0.829593099	5.362427678	1.46E-06	0.000230104	0.03601213	5.362427678	0.833859637	1	-0.017912644	5.362427678	0.917061196	1	orange
A430046D13Rik	-1.497843719	2.531415326	1.81E-07	4.21E-05	-1.58109032	2.531415326	1.26E-06	0.000204986	0.053942502	2.531415326	0.867070135	1	-0.029296099	2.531415326	0.935281287	1	orange
Fgf11	-0.932015504	3.550254773	5.12E-05	0.003831278	-1.109193482	3.550254773	1.83E-05	0.001653759	0.14328301	3.550254773	0.574311133	1	-0.033892817	3.550254773	0.894215403	1	orange
Irgm2	-0.736867934	5.830855292	0.000207449	0.010942234	-0.938663314	5.830855292	2.45E-05	0.00206418	0.176302632	5.830855292	0.423816956	1	-0.025492434	5.830855292	0.908042021	1	orange
Banp	-1.276646412	3.582021071	4.59E-07	9.58E-05	-1.671369997	3.582021071	7.74E-09	2.85E-06	0.236346268	3.582021071	0.398451686	1	-0.158373342	3.582021071	0.559344574	1	orange
Wscd2	-1.313493448	3.016627167	9.76E-06	0.001165596	-1.673893706	3.016627167	7.98E-07	0.000142988	0.185113649	3.016627167	0.573698368	1	-0.175280293	3.016627167	0.579128729	1	orange
Sox6	-1.065430531	4.946596431	1.04E-08	3.83E-06	-1.235162681	4.946596431	3.89E-09	1.70E-06	0.106115793	4.946596431	0.607175237	1	-0.063615307	4.946596431	0.746986402	1	orange
Adrb3	-2.214191886	7.772984615	2.65E-32	3.70E-28	-2.546636798	7.772984615	1.51E-31	2.11E-27	0.127484004	7.772984615	0.535519077	1	-0.20496042	7.772984615	0.284120379	1	orange
Gbp6	-1.245335944	5.164081029	1.09E-13	1.18E-10	-1.547304272	5.164081029	4.28E-16	1.49E-12	0.124485607	5.164081029	0.502050571	1	-0.177481366	5.164081029	0.309209162	1	orange
Angpt1	-0.943601391	4.487877528	1.85E-06	0.000304474	-1.227894426	4.487877528	3.60E-08	1.03E-05	0.125116107	4.487877528	0.568609194	1	-0.159175359	4.487877528	0.440476112	1	orange
Tln2	-0.694995751	6.276381903	1.68E-05	0.001684372	-0.920739596	6.276381903	3.47E-07	7.23E-05	0.072523494	6.276381903	0.68586613	1	-0.153220002	6.276381903	0.356365189	1	orange
Six5	-0.673544782	4.179913996	0.000351583	0.01660044	-0.805695325	4.179913996	0.000128975	0.007736271	0.036454815	4.179913996	0.863100292	1	-0.095694452	4.179913996	0.628691302	1	orange
Prag1	-0.698582311	6.953212756	0.000160739	0.009641558	-0.799320756	6.953212756	0.000111986	0.006925286	-0.00088615	6.953212756	0.997058569	1	-0.101624393	6.953212756	0.596050295	1	orange
Slc45a3	-0.755288378	4.885028188	0.000852617	0.031441109	-0.890098156	4.885028188	0.000456836	0.019705984	-0.044922074	4.885028188	0.859487875	1	-0.179730742	4.885028188	0.445806028	1	orange
Plekhm1	-0.541342951	5.949697631	0.00065133	0.026462186	-0.612168749	5.949697631	0.000530747	0.022342528	-0.044407526	5.949697631	0.801261599	1	-0.115232967	5.949697631	0.480540564	1	orange
Strip2	-1.115529458	3.814169989	5.54E-06	0.000758602	-1.149722335	3.814169989	3.33E-05	0.002571221	-0.108262385	3.814169989	0.691472389	1	-0.142452204	3.814169989	0.584211418	1	orange
Tgm1	-1.609761787	4.750428461	1.29E-14	1.50E-11	-1.585634878	4.750428461	2.47E-11	1.82E-08	-0.190507595	4.750428461	0.41020394	1	-0.16637826	4.750428461	0.447154351	1	orange
Med12l	-1.183309431	4.167770506	2.11E-07	4.69E-05	-1.189343919	4.167770506	3.75E-06	0.000503294	-0.061078621	4.167770506	0.810027059	1	-0.067110949	4.167770506	0.785329239	1	orange
Tssc1	-0.625695763	3.852730019	0.001454548	0.046625615	-0.619233189	3.852730019	0.004698952	0.099503864	-0.041644272	3.852730019	0.849919964	1	-0.035180355	3.852730019	0.87158472	1	orange
Abca2	-0.654351623	6.528256623	0.000179909	0.010476722	-0.631256845	6.528256623	0.001185673	0.039738534	-0.059332113	6.528256623	0.759742265	1	-0.036237112	6.528256623	0.845058543	1	orange
Tbc1d4	-0.790620768	5.785735208	7.92E-07	0.00015169	-0.738770436	5.785735208	3.62E-05	0.002748981	-0.082647852	5.785735208	0.641617903	1	-0.030797077	5.785735208	0.857789862	1	orange
Pomt1	-0.684795803	5.326331966	0.000139267	0.008767544	-0.622024355	5.326331966	0.001905833	0.055376124	-0.078098995	5.326331966	0.695737373	1	-0.01532704	5.326331966	0.941055719	1	orange
Tmem94	-0.750340032	5.717633305	6.41E-07	0.000126168	-0.520693163	5.717633305	0.001917023	0.055470642	-0.151577281	5.717633305	0.363975174	1	0.078069931	5.717633305	0.606083349	1	orange
Rgs2	-1.368153619	6.716138857	2.43E-13	2.13E-10	-0.881382566	6.716138857	2.55E-05	0.002099505	-0.293528646	6.716138857	0.157454974	1	0.193242679	6.716138857	0.303291411	1	orange
Ddhd1	-0.907871802	5.185387903	1.68E-07	4.06E-05	-0.596861408	5.185387903	0.002058932	0.05801538	-0.194263284	5.185387903	0.312447312	1	0.116747672	5.185387903	0.504357897	1	orange
1190005I06Rik	-1.212162516	2.943547153	2.26E-05	0.002049444	-0.708372127	2.943547153	0.027004465	0.259927279	-0.326316892	2.943547153	0.303371511	1	0.177476947	2.943547153	0.540323244	1	orange
Rnf125	-0.699034433	4.752253022	5.11E-05	0.003831278	-0.518689838	4.752253022	0.006961232	0.124430753	-0.128113256	4.752253022	0.50249987	1	0.052232002	4.752253022	0.762308049	1	orange
Relb	-0.816394099	4.246687777	3.25E-05	0.002595765	-0.629095578	4.246687777	0.004167352	0.09172112	-0.176151613	4.246687777	0.418611606	1	0.011148192	4.246687777	0.950938145	1	orange
Cables1	-0.855429535	4.824234624	2.25E-05	0.002049444	-0.659362366	4.824234624	0.003435665	0.079952888	-0.163010931	4.824234624	0.467183606	1	0.033057075	4.824234624	0.867739596	1	orange
9530052E02Rik	-0.800957506	3.728880714	0.000462225	0.020187667	-0.623642733	3.728880714	0.014657067	0.188451851	-0.113360093	3.728880714	0.655566503	1	0.063956144	3.728880714	0.779751424	1	orange
Trim68	-0.846493088	4.356160415	4.19E-06	0.000616568	-0.7007038	4.356160415	0.000651714	0.026173436	-0.129903658	4.356160415	0.52375245	1	0.015886799	4.356160415	0.928963253	1	orange
Lrig1	-0.761855022	6.467814333	4.85E-07	9.79E-05	-0.616549831	6.467814333	0.000258827	0.013106377	-0.132391053	6.467814333	0.430512583	1	0.012914386	6.467814333	0.928225212	1	orange
Vegfa	-0.769665659	8.135130387	0.00089649	0.032498532	-0.606434905	8.135130387	0.019135615	0.215233848	-0.155737437	8.135130387	0.545962954	1	0.007493397	8.135130387	0.966041164	1	orange
Ptx3	-0.991250365	6.060459833	0.000210882	0.011038524	-0.842550895	6.060459833	0.005017375	0.102287048	-0.192976622	6.060459833	0.517268343	1	-0.044276656	6.060459833	0.881625446	1	orange
Cachd1	-0.713520147	5.521571332	0.000185578	0.010600388	-0.646034488	5.521571332	0.002420298	0.063822801	-0.06779545	5.521571332	0.748921091	1	-0.000309352	5.521571332	0.995399718	1	orange
Nudt6	-0.934713041	3.819579223	2.63E-05	0.002200348	-0.808946823	3.819579223	0.001160484	0.039176151	-0.097165448	3.819579223	0.693756876	1	0.028602533	3.819579223	0.895977611	1	orange
Pag1	-0.600974832	4.356394469	0.000932637	0.033507808	-0.547765627	4.356394469	0.006715785	0.121895851	-0.030730198	4.356394469	0.880119864	1	0.02247974	4.356394469	0.89858718	1	orange
Kcnk2	-1.186145755	3.813618402	2.24E-05	0.002049444	-0.964796839	3.813618402	0.002160364	0.059552755	-0.126945548	3.813618402	0.683272598	1	0.094405349	3.813618402	0.735064539	1	orange
Irs2	-0.731877196	7.934721427	0.000194055	0.010600388	-0.613861493	7.934721427	0.005078079	0.102708014	-0.04710401	7.934721427	0.829140402	1	0.070911758	7.934721427	0.714863958	1	orange
Fgf2	-1.967073406	1.72458226	2.82E-08	8.55E-06	-1.688162756	1.72458226	2.70E-05	0.002191277	-0.11493879	1.72458226	0.766810601	1	0.163986256	1.72458226	0.662048196	1	orange
Nfkb2	-0.775090121	5.441027062	2.36E-05	0.00208639	-0.650980772	5.441027062	0.001459049	0.045721231	-0.025936197	5.441027062	0.898830457	1	0.098173493	5.441027062	0.592661751	1	orange
Fam13a	-1.53970795	7.958372205	3.20E-10	1.79E-07	-1.348329046	7.958372205	1.12E-06	0.000186296	0.003249886	7.958372205	0.990535902	1	0.194628905	7.958372205	0.425082894	1	orange
Mir3058	-3.16855069	-2.584259636	0.000449832	0.019769989	-2.519034845	-2.584259636	0.010709917	0.156648581	-0.092299168	-2.584259636	0.942856736	1	0.557563356	-2.584259636	0.826521764	1	orange
Gbp11	-2.339827976	2.164718801	2.44E-13	2.13E-10	-1.942524575	2.164718801	8.01E-08	2.15E-05	-0.038387623	2.164718801	0.914379346	1	0.358924813	2.164718801	0.277089017	1	orange
Atp1b2	-0.653103952	4.684044138	0.000514723	0.022134662	-0.572419356	4.684044138	0.006224112	0.11646666	0.010331254	4.684044138	0.961863689	1	0.091016293	4.684044138	0.629561733	1	orange
A830010M20Rik	-0.9872205	3.183361532	3.10E-05	0.002506953	-0.863515356	3.183361532	0.001129739	0.038323378	0.028077622	3.183361532	0.917955004	1	0.151784521	3.183361532	0.529024019	1	orange
Hist2h2be	-0.769566398	3.436620329	0.000949363	0.033847699	-0.667936205	3.436620329	0.010072385	0.152123343	0.045031274	3.436620329	0.864162671	1	0.146662416	3.436620329	0.532806411	1	orange
Dock4	-0.662356839	4.647291355	0.000447862	0.019745502	-0.55515112	4.647291355	0.008171764	0.13779361	0.008208387	4.647291355	0.970416295	1	0.115414513	4.647291355	0.543171503	1	orange
Exd1	-1.069920231	2.200853998	0.000465267	0.020257227	-0.802883486	2.200853998	0.018979239	0.214478284	-0.060879905	2.200853998	0.860236216	1	0.206160376	2.200853998	0.508502382	1	orange
Rims4	-1.40854062	0.676488733	0.001466427	0.046767604	-1.030132424	0.676488733	0.038787831	0.320958397	-0.116287225	0.676488733	0.813609869	1	0.262134723	0.676488733	0.57836856	1	orange
Hps4	-0.742724705	3.939927796	0.000194927	0.010600388	-0.552577604	3.939927796	0.012825592	0.173692314	-0.061042436	3.939927796	0.783093769	1	0.129105506	3.939927796	0.521529424	1	orange
Peli3	-1.077657623	2.120872624	0.000231242	0.011925598	-0.809764277	2.120872624	0.013913582	0.182587997	-0.091318919	2.120872624	0.783666333	1	0.176578785	2.120872624	0.550174449	1	orange
Mfsd2a	-2.253776893	5.546515477	4.37E-10	2.26E-07	-1.677373021	5.546515477	4.08E-05	0.003030799	-0.216030049	5.546515477	0.587594474	1	0.360374643	5.546515477	0.312010387	1	orange
Ano8	-0.64309615	4.514170041	0.000198398	0.010728997	-0.510776298	4.514170041	0.007866158	0.134639363	-0.041828098	4.514170041	0.828137709	1	0.090492293	4.514170041	0.602822779	1	orange
Uck1	-0.681086945	7.148670482	0.000940454	0.033702021	-0.535808578	7.148670482	0.019540618	0.217436047	-0.046849796	7.148670482	0.837917904	1	0.098428661	7.148670482	0.630409543	1	orange
Limch1	-0.855038029	4.991831987	1.31E-05	0.001451015	-0.66509173	4.991831987	0.002387796	0.063380503	-0.090044546	4.991831987	0.680178933	1	0.099902321	4.991831987	0.611489946	1	orange
Abcd4	-0.681246581	3.879952358	0.000663599	0.026727537	-0.466455381	3.879952358	0.036293998	0.307302421	-0.084861925	3.879952358	0.703314356	1	0.129930088	3.879952358	0.51927666	1	orange
Arid5b	-0.873971361	6.597068664	2.00E-06	0.000325598	-0.584417438	6.597068664	0.00438325	0.095124697	-0.121690339	6.597068664	0.55143261	1	0.167863738	6.597068664	0.362892437	1	orange
Cish	-1.666381143	5.105952965	4.95E-08	1.36E-05	-1.051346549	5.105952965	0.00220503	0.060545173	-0.296491276	5.105952965	0.38264051	1	0.318544097	5.105952965	0.296387785	1	orange
Ksr1	-0.754074578	5.026602629	0.000192678	0.010600388	-0.484759205	5.026602629	0.031402633	0.284619452	-0.088678635	5.026602629	0.692786064	1	0.180637068	5.026602629	0.374285504	1	orange
Baiap2l2	-2.056362028	1.24111884	9.08E-07	0.000169117	-1.221953815	1.24111884	0.008936901	0.144369474	-0.098304548	1.24111884	0.833076526	1	0.736103383	1.24111884	0.090266345	1	orange
Saa2	-1.966880393	-1.416332942	0.001169757	0.039584811	-0.993129409	-1.416332942	0.126399323	0.566068691	0.055198621	-1.416332942	0.965108926	1	1.028873868	-1.416332942	0.130549982	1	orange
Ric8b	-0.676624837	5.091267977	2.79E-05	0.002309255	-0.411426763	5.091267977	0.021557874	0.230170238	0.067078905	5.091267977	0.707892457	1	0.332276887	5.091267977	0.040833906	0.821143416	orange
Ptpn14	-0.826403514	6.515269748	1.04E-06	0.000191482	-0.582739245	6.515269748	0.001985467	0.056746199	0.125307591	6.515269748	0.504797626	1	0.368971824	6.515269748	0.030000411	0.712887181	orange
Srgap3	-1.188706842	4.027515467	6.24E-06	0.000838903	-0.874459071	4.027515467	0.002967646	0.072839404	0.186523078	4.027515467	0.523641702	1	0.500770548	4.027515467	0.05869212	0.962015487	orange
Ikzf2	-1.043714274	2.848292838	0.000871667	0.031974853	-0.85266512	2.848292838	0.014954656	0.190262765	0.230996288	2.848292838	0.507683258	1	0.422044881	2.848292838	0.180296973	1	orange
Oscp1	-0.756743439	3.939873557	0.00153179	0.048435066	-0.622037117	3.939873557	0.019155386	0.215233848	0.223376777	3.939873557	0.398406219	1	0.358082675	3.939873557	0.136164871	1	orange
Klhl24	-0.598953285	6.482179126	0.000610632	0.025324032	-0.530906034	6.482179126	0.0062877	0.117169187	0.206965087	6.482179126	0.285514683	1	0.275012281	6.482179126	0.116683209	1	orange
4930480K23Rik	-1.177874942	1.533950808	0.000317958	0.015212199	-0.986855852	1.533950808	0.006279033	0.117163911	0.343252936	1.533950808	0.336276035	1	0.534268313	1.533950808	0.108677963	1	orange
Pak6	-1.053065514	3.660929124	9.36E-05	0.006288961	-1.054803192	3.660929124	0.000478295	0.020505074	0.36802685	3.660929124	0.217638968	1	0.366288941	3.660929124	0.177798639	1	orange
Adamts4	-0.896925654	6.62545231	0.000146053	0.008992255	-0.900201364	6.62545231	0.000676362	0.026787291	0.332560554	6.62545231	0.206039288	1	0.329284806	6.62545231	0.16419583	1	orange
Atat1	-0.72893855	4.065151302	0.000251925	0.012849992	-0.750741949	4.065151302	0.000683742	0.026994278	0.33021957	4.065151302	0.132995205	0.982421237	0.308415772	4.065151302	0.124716416	1	orange
Ank	-0.883359173	5.962246699	4.34E-06	0.000625835	-0.79138274	5.962246699	0.00022621	0.011885403	0.350338267	5.962246699	0.100667205	0.899993457	0.442314516	5.962246699	0.022014206	0.621556641	orange
Sec31b	-0.776111555	3.278482587	0.001556984	0.049009939	-0.650551622	3.278482587	0.016812913	0.20221796	0.303215752	3.278482587	0.263174561	1	0.428774446	3.278482587	0.082526757	1	orange
Ddo	-0.995802137	3.846101285	2.79E-05	0.002309255	-0.78256336	3.846101285	0.00303381	0.073997431	0.394204741	3.846101285	0.133194456	0.982854129	0.607442125	3.846101285	0.011100374	0.426205566	orange
Acss2os	-1.328292409	4.761805379	0.001246428	0.041739146	-0.977431391	4.761805379	0.034931168	0.301170882	0.50687865	4.761805379	0.270785251	1	0.857738474	4.761805379	0.036918088	0.784121507	orange
Mapk1ip1	-0.640342957	3.982250057	0.000842878	0.031413503	-0.4841445	3.982250057	0.022577382	0.235654588	0.194124437	3.982250057	0.359538088	1	0.350322385	3.982250057	0.069622855	1	orange
Coq8a	-1.267060955	5.964180488	8.33E-13	6.47E-10	-0.91947312	5.964180488	3.40E-06	0.000461389	0.285331018	5.964180488	0.146149768	0.994029995	0.632918631	5.964180488	0.000374723	0.043282012	orange
Atp1a3	-1.55669325	5.100076104	5.60E-05	0.004141367	-0.96802435	5.100076104	0.02576422	0.253075701	0.285182327	5.100076104	0.507733948	1	0.873850504	5.100076104	0.023401274	0.633191108	orange
Acaca	-1.191773412	10.79327996	7.36E-05	0.005218393	-0.764526504	10.79327996	0.023350484	0.24013713	0.349394775	10.79327996	0.297849565	1	0.776641669	10.79327996	0.00975495	0.396323192	orange
Fasn	-1.173653235	13.49489746	0.000207477	0.010942234	-0.730542661	13.49489746	0.039513809	0.324357628	0.41367731	13.49489746	0.242011976	1	0.856787881	13.49489746	0.006737913	0.313896921	orange
Grk3	-0.826176217	5.224991488	2.20E-05	0.002038088	-0.521031016	5.224991488	0.016010525	0.196785932	0.309828583	5.224991488	0.151226379	0.999246012	0.614973207	5.224991488	0.001628966	0.130093848	orange
Elovl6	-1.795651745	9.196748276	2.02E-07	4.62E-05	-0.871090394	9.196748276	0.02422061	0.244409562	0.400659823	9.196748276	0.297117934	1	1.32522108	9.196748276	0.000116593	0.016974008	orange
Rorc	-1.092135708	4.64136973	3.12E-09	1.25E-06	-0.305816063	4.64136973	0.13475673	0.578238515	-0.012166086	4.64136973	0.953257245	1	0.774152863	4.64136973	2.89E-05	0.005935889	orange
Neil2	-1.199340609	1.362695796	0.000700548	0.02779717	-0.181373707	1.362695796	0.646506331	0.940363476	-0.069521213	1.362695796	0.860628179	1	0.946096518	1.362695796	0.008030985	0.350318033	orange
Mir23b	-5.356854426	-3.737631899	0.000846605	0.031441109	-0.291789184	-3.737631899	1	1	-1.341134155	-3.737631899	0.268212815	1	3.711698709	-3.737631899	0.142983931	1	orange
Gm15441	-0.635384134	8.64046549	0.000755903	0.029047101	-0.133836303	8.64046549	0.522939404	0.900170012	-0.155521989	8.64046549	0.457923829	1	0.346025843	8.64046549	0.067110432	1	orange
Txnip	-0.603484247	10.68356812	0.001469017	0.046767604	-0.147019919	10.68356812	0.485326767	0.885297579	-0.120215672	10.68356812	0.568287977	1	0.336248656	10.68356812	0.076810955	1	orange
Bcas1	-1.169242269	1.988719041	0.000617209	0.025521036	-0.410046502	1.988719041	0.282888522	0.755019189	-0.21161149	1.988719041	0.578663146	1	0.547584522	1.988719041	0.111663507	1	orange
Apol10b	-0.91758102	3.105428406	0.000841166	0.031413503	-0.41350359	3.105428406	0.17763197	0.64377745	-0.113467576	3.105428406	0.712522248	1	0.390610199	3.105428406	0.158188472	1	orange
Hs3st3b1	-1.11425527	3.717919476	4.24E-05	0.003277589	-0.417864594	3.717919476	0.169901385	0.633887281	-0.312839796	3.717919476	0.301353045	1	0.383551769	3.717919476	0.161727717	1	orange
Nr3c2	-0.826471534	4.166840058	0.000656256	0.026581036	-0.432018806	4.166840058	0.110571934	0.532696776	-0.144315483	4.166840058	0.592916446	1	0.250137786	4.166840058	0.304600582	1	orange
Cited2	-0.876948798	6.376686549	1.47E-08	4.89E-06	-0.563042454	6.376686549	0.001107885	0.037950499	-0.259347705	6.376686549	0.131323708	0.978347623	0.054558938	6.376686549	0.723824983	1	brown
Gfod2	-0.890122478	3.531486466	3.73E-05	0.002929871	-0.519433384	3.531486466	0.031705081	0.28624691	-0.363413001	3.531486466	0.128788783	0.972421414	0.007278722	3.531486466	0.970986703	1	brown
Igfbp5	-1.186385781	9.79314182	2.63E-09	1.08E-06	-0.650161238	9.79314182	0.00349947	0.080707258	-0.475878603	9.79314182	0.032208086	0.605841474	0.060345981	9.79314182	0.758211702	1	brown
Ifi47	-0.907076909	4.48636319	0.000387169	0.017683244	-0.541413007	4.48636319	0.058292313	0.395098623	-0.327830536	4.48636319	0.249732387	1	0.037834621	4.48636319	0.876888272	1	brown
4930412C18Rik	-1.19174569	1.641816586	0.000191064	0.010600388	-0.550193511	1.641816586	0.127164823	0.56726957	-0.427017292	1.641816586	0.227348405	1	0.214543233	1.641816586	0.513440503	1	brown
Arntl	-1.079691075	4.275934798	4.14E-08	1.16E-05	-0.396121068	4.275934798	0.071914915	0.438517827	-0.48265821	4.275934798	0.02716441	0.555043565	0.200913114	4.275934798	0.313881051	1	brown
Gm13889	-1.050130392	3.827244875	3.23E-06	0.000494542	-0.399710891	3.827244875	0.113015133	0.537179221	-0.44283191	3.827244875	0.077150487	0.822467745	0.207589256	3.827244875	0.363361461	1	brown
Syt3	-1.293319154	1.775560341	0.00012627	0.008246505	-0.375977053	1.775560341	0.325577308	0.790959397	-0.519541059	1.775560341	0.164764407	1	0.397806559	1.775560341	0.251529595	1	brown
St3gal4	-0.723861926	5.185874374	3.99E-05	0.003118561	-0.204345674	5.185874374	0.297921557	0.768645316	-0.32465847	5.185874374	0.096791387	0.888392991	0.194858196	5.185874374	0.271679922	1	brown
Nmnat3	-0.740014638	4.069389513	0.00011416	0.007561636	-0.237687452	4.069389513	0.267371532	0.740071375	-0.37944152	4.069389513	0.074402062	0.812856567	0.122886899	4.069389513	0.525452618	1	brown

Thbs1	-0.938095728	9.077791328	0.000385978	0.017683244	-0.339592636	9.077791328	0.249474598	0.725182207	-0.487388616	9.077791328	0.098700054	0.892836214	0.111114527	9.077791328	0.669530024	1	brown
Sytl2	-1.093555014	3.366107839	3.48E-05	0.002747378	-0.435677103	3.366107839	0.141693638	0.591573599	-0.600601934	3.366107839	0.041483254	0.666634647	0.05727959	3.366107839	0.825883027	1	brown
B230311B06Rik	-1.480562403	0.007987519	0.001164085	0.03948849	-0.54706989	0.007987519	0.3120534	0.781307473	-0.905979401	0.007987519	0.074204339	0.812758499	0.027569295	0.007987519	0.954187463	1	brown
5930412G12Rik	-1.079093155	2.115294763	0.00157661	0.049405162	-0.137252637	2.115294763	0.730222383	0.965045279	-0.729335856	2.115294763	0.056923112	0.746059679	0.212511888	2.115294763	0.537098694	1	brown
Adm	-0.602958972	4.535425075	0.0008515	0.031441109	-0.06014219	4.535425075	0.766959996	0.970758052	-0.406082838	4.535425075	0.042960496	0.67446002	0.13673472	4.535425075	0.451820893	1	brown
Pinc	-3.779720452	-0.621354285	3.40E-11	2.16E-08	0.114486002	-0.621354285	0.913063154	0.998835231	-2.105816956	-0.621354285	0.000427209	0.046381842	1.78833287	-0.621354285	0.003822536	0.227921322	brown
Lalba	-2.406830958	1.547101455	2.44E-06	0.000387896	0.146349243	1.547101455	0.807408079	0.976199887	-1.78229871	1.547101455	0.002047022	0.1339501	0.770896862	1.547101455	0.129625448	1	brown
Csf2rb	-0.660679812	6.146721353	0.000162011	0.009676361	-0.064793711	6.146721353	0.739855222	0.965045279	-0.528169704	6.146721353	0.006725622	0.271668487	0.067716766	6.146721353	0.697667492	1	brown
Syt15	-1.010601828	3.129374267	0.000234394	0.011999621	-0.115079498	3.129374267	0.712545852	0.96143472	-0.806900948	3.129374267	0.008764152	0.314351894	0.088625839	3.129374267	0.748488668	1	brown
Csf2rb2	-0.765084399	5.224045738	0.000182095	0.010516358	-0.105660306	5.224045738	0.643597857	0.939498978	-0.630786602	5.224045738	0.005654473	0.245803133	0.028638372	5.224045738	0.884697152	1	brown
Slc27a6	-1.358890382	1.433418997	0.000549509	0.023202217	-0.177677737	1.433418997	0.707706299	0.961245088	-1.166418299	1.433418997	0.008686781	0.313711747	0.014816671	1.433418997	0.969073959	1	brown
Ccl12	-1.784654553	1.484999424	1.50E-05	0.001552812	-0.547177108	1.484999424	0.244105302	0.72206739	-1.416239155	1.484999424	0.002449944	0.150199913	-0.178728951	1.484999424	0.701319014	1	brown
Thbd	-0.760164014	7.0946841	6.90E-06	0.000909632	-0.356514234	7.0946841	0.058139294	0.394416599	-0.477942316	7.0946841	0.011060671	0.36012661	-0.07429228	7.0946841	0.671687527	1	brown
Gulp1	-0.693209495	5.300684157	0.000468597	0.020338865	-0.289380863	5.300684157	0.190446542	0.662262313	-0.432560812	5.300684157	0.049578534	0.708496518	-0.028731432	5.300684157	0.895550957	1	brown
St3gal2	-0.775471428	5.839737123	1.96E-05	0.001877839	-0.369797648	5.839737123	0.067723607	0.425586838	-0.454182424	5.839737123	0.024604637	0.530027096	-0.048508067	5.839737123	0.800518131	1	brown
Socs2	-0.744589848	4.639219076	0.000520703	0.02225488	-0.370272948	4.639219076	0.122610075	0.558129292	-0.442845143	4.639219076	0.063507205	0.77496143	-0.068526908	4.639219076	0.76417	1	brown
Sirpa	-0.663243408	5.603544865	0.000270675	0.013510561	-0.410020942	5.603544865	0.04333841	0.342395489	-0.383256639	5.603544865	0.058154529	0.752562678	-0.130033482	5.603544865	0.489671669	1	brown
Ccl2	-1.146075039	4.930533346	0.000401585	0.018105014	-0.740376974	4.930533346	0.041849886	0.333954643	-0.565787634	4.930533346	0.118464988	0.945142756	-0.160447853	4.930533346	0.641073107	1	brown
Irf4	-2.100292277	7.110242922	9.46E-18	1.65E-14	-1.292260503	7.110242922	2.58E-06	0.000371945	-1.174709012	7.110242922	1.76E-05	0.004740051	-0.366676385	7.110242922	0.145046191	1	brown
Ccl7	-1.166443973	4.115208665	0.0007741	0.029398971	-0.77422615	4.115208665	0.047768307	0.35887623	-0.724589178	4.115208665	0.062629173	0.771874186	-0.33236739	4.115208665	0.363249911	1	brown
Lrrn4	-1.171232299	1.288872807	0.000785993	0.029609255	-0.803208939	1.288872807	0.044070101	0.34505531	-0.722936643	1.288872807	0.062332302	0.771874186	-0.354884027	1.288872807	0.347255967	1	brown
Oasl1	-1.538990009	3.503562359	7.18E-06	0.000937525	-0.957825782	3.503562359	0.013679472	0.181217344	-0.968048365	3.503562359	0.01192471	0.377938143	-0.386876111	3.503562359	0.28591526	1	brown
Serpina3k	-1.711192255	1.834559805	0.000947665	0.033847699	-0.853619147	1.834559805	0.147813486	0.599715571	-1.262928592	1.834559805	0.031493577	0.601303591	-0.405325904	1.834559805	0.468508762	1	brown
Cmah	-0.916512518	4.532624702	0.000547584	0.023191022	-0.659795671	4.532624702	0.026791121	0.258764824	-0.659797635	4.532624702	0.02613458	0.541591024	-0.403077988	4.532624702	0.14505402	1	brown
Gfra2	-0.969219797	4.35697012	0.000607513	0.02526965	-0.581251076	4.35697012	0.067622031	0.425139681	-0.895559606	4.35697012	0.004803894	0.22454591	-0.50758702	4.35697012	0.08610077	1	brown
Plppr3	-0.658924494	5.776090002	0.000708409	0.027861808	-0.397779682	5.776090002	0.06704582	0.423295188	-0.646895854	5.776090002	0.002862037	0.167363302	-0.385750024	5.776090002	0.054778411	0.935725048	brown
Prtn3	-1.229556262	3.266739602	0.004647458	0.103924598	-1.498038236	3.266739602	0.00293653	0.072510512	-1.427950644	3.266739602	0.004012422	0.203122991	-1.696407338	3.266739602	0.000403459	0.045109948	brown
AW551984	-1.325991072	2.795901179	0.000304455	0.014877857	-1.150536094	2.795901179	0.006065999	0.114410803	-0.766556244	2.795901179	0.061983359	0.770026154	-0.591087076	2.795901179	0.129375903	1	brown
Npas2	-1.180750654	3.382433944	7.48E-06	0.000958492	-0.976668064	3.382433944	0.00111745	0.038091405	-0.686195612	3.382433944	0.019635571	0.4711954	-0.482105252	3.382433944	0.084382815	1	brown
Rgs16	-0.750053033	5.931507322	0.000648627	0.02642919	-0.625033593	5.931507322	0.011041753	0.159557034	-0.399158122	5.931507322	0.10311584	0.906449992	-0.274137936	5.931507322	0.228191508	1	brown
Spon2	-1.067539922	5.633705632	8.51E-06	0.001052705	-1.006766958	5.633705632	0.00019468	0.010612473	-0.566593224	5.633705632	0.034293237	0.618332787	-0.505818717	5.633705632	0.042565846	0.848645178	brown
Xbp1	-0.726099028	7.979722648	3.13E-05	0.002511027	-0.701235871	7.979722648	0.00031655	0.015255525	-0.368470325	7.979722648	0.057389369	0.746809887	-0.343606975	7.979722648	0.055314319	0.938195297	brown
Sh3pxd2a	-0.928687941	8.975523899	2.41E-06	0.000387303	-0.994503087	8.975523899	6.95E-06	0.000777344	-0.421996466	8.975523899	0.054446206	0.732627562	-0.487811477	8.975523899	0.016521909	0.5236059	brown
Ifnb1	-3.071606689	-1.527334171	8.26E-06	0.001039402	-7.258851241	-1.527334171	4.39E-06	0.000557251	-1.247289436	-1.527334171	0.090931535	0.868666527	-5.404518287	-1.527334171	0.006205708	0.304425461	brown
Cpne2	-0.747600655	4.929698354	0.000125839	0.008246505	-0.863377072	4.929698354	7.97E-05	0.005253018	-0.27725628	4.929698354	0.199892667	1	-0.393031003	4.929698354	0.051696626	0.908820182	brown
Srgap1	-0.610841366	4.179620075	0.001458315	0.046639376	-0.648661985	4.179620075	0.002508518	0.065408669	-0.204801376	4.179620075	0.335493211	1	-0.242620037	4.179620075	0.22271874	1	brown
Ccl4	-1.352197344	3.364187985	0.00021204	0.011057752	-1.30880937	3.364187985	0.00168867	0.050863891	-0.50466182	3.364187985	0.216086788	1	-0.461265646	3.364187985	0.234612722	1	brown
Steap3	-0.714855145	5.591406783	0.00026733	0.013431649	-0.707020602	5.591406783	0.001261057	0.041178814	-0.246755999	5.591406783	0.257285505	1	-0.238920645	5.591406783	0.237825693	1	brown
Ifit1	-0.92665295	4.690128965	0.00023239	0.011940767	-0.742434113	4.690128965	0.008520827	0.139609704	-0.306543198	4.690128965	0.274427976	1	-0.122322866	4.690128965	0.644656832	1	brown
Nfil3	-1.073431605	6.030533908	1.21E-06	0.000214098	-0.745334308	6.030533908	0.002626105	0.066975252	-0.4185298	6.030533908	0.089249272	0.862619516	-0.090431852	6.030533908	0.697041443	1	brown
Hcar1	-1.447263243	6.241284203	6.74E-08	1.78E-05	-1.253715092	6.241284203	3.54E-05	0.002704425	-0.522346845	6.241284203	0.080603316	0.836876692	-0.328797689	6.241284203	0.237506264	1	brown
Itsn1	-0.685848329	6.186717131	1.49E-05	0.001552812	-0.635899845	6.186717131	0.000319468	0.015291874	-0.236749847	6.186717131	0.177867819	1	-0.186800887	6.186717131	0.251257589	1	brown
Ar	-0.681372312	5.438873059	1.01E-05	0.00119424	-0.634293607	5.438873059	0.000230069	0.011978651	-0.214662095	5.438873059	0.209138044	1	-0.167582633	5.438873059	0.291831121	1	brown
St6galnac5	-1.569199961	2.58840176	1.08E-05	0.001241955	-1.332551615	2.58840176	0.001040539	0.036520641	-0.515770219	2.58840176	0.19320776	1	-0.27910906	2.58840176	0.470993048	1	brown
Hs1bp3	-0.627857654	4.310634719	0.000706855	0.027861808	-0.554929881	4.310634719	0.007308238	0.129291059	-0.187438452	4.310634719	0.36184946	1	-0.114509296	4.310634719	0.554116198	1	brown
Tppp	-1.289660716	4.968984562	3.83E-13	3.15E-10	-1.117828387	4.968984562	2.44E-08	7.75E-06	-0.394127706	4.968984562	0.045386546	0.682801253	-0.222293489	4.968984562	0.229103371	1	brown
Prkag2	-0.693271643	5.540929019	0.000127109	0.00826265	-0.61430589	5.540929019	0.002334895	0.062394835	-0.17170932	5.540929019	0.392113901	1	-0.092742976	5.540929019	0.621656682	1	brown
Irs1	-0.893616105	7.008049318	4.31E-05	0.003313484	-0.788231516	7.008049318	0.001275807	0.041466688	-0.22404485	7.008049318	0.356857609	1	-0.118659983	7.008049318	0.601753316	1	brown
Ldb2	-0.588982462	4.663370782	0.000984892	0.034677726	-0.57843796	4.663370782	0.003685805	0.083541409	-0.134756982	4.663370782	0.49662308	1	-0.12421146	4.663370782	0.502490331	1	brown
Tmprss13	-0.769389372	4.793743504	8.88E-06	0.001088992	-0.776907645	4.793743504	6.12E-05	0.004253036	-0.15540596	4.793743504	0.418585308	1	-0.162923008	4.793743504	0.365036935	1	brown
Pm20d2	-1.067521454	3.424284715	8.87E-05	0.006018955	-1.088134476	3.424284715	0.000419775	0.018565751	-0.290938705	3.424284715	0.336018275	1	-0.311546411	3.424284715	0.276599854	1	brown
Slc5a3	-0.854709778	6.025793453	0.001390726	0.044992576	-1.064137225	6.025793453	0.0004124	0.018344482	-0.100016143	6.025793453	0.737102562	1	-0.309442877	6.025793453	0.265016864	1	cyan
Steap4	-0.980202911	8.463136341	4.03E-05	0.003127965	-1.224233411	8.463136341	5.66E-06	0.000666561	-0.097654758	8.463136341	0.713345855	1	-0.341685106	8.463136341	0.166336685	1	cyan
C1rl	-0.701894005	3.831040096	0.001497751	0.047466141	-0.911088998	3.831040096	0.000238387	0.012248862	-0.045592393	3.831040096	0.854070591	1	-0.254704832	3.831040096	0.268550729	1	cyan
Napepld	-0.689647641	4.108989061	0.004569007	0.102934021	-0.903479077	4.108989061	0.000927863	0.033858505	-0.029149521	4.108989061	0.915503885	1	-0.242978938	4.108989061	0.337392313	1	cyan
Cxcl1	-0.695566726	7.314174234	0.005153359	0.111147144	-0.891173086	7.314174234	0.001402418	0.044177329	-0.098027037	7.314174234	0.723264629	1	-0.293633147	7.314174234	0.253846977	1	cyan
Igf1	-0.792079007	7.61866596	0.000439871	0.019578451	-1.039974681	7.61866596	4.07E-05	0.003030799	-0.189786263	7.61866596	0.449097309	1	-0.437681656	7.61866596	0.060320986	0.967905971	cyan
Ablim2	-0.899128332	2.909407527	0.000866881	0.031882973	-1.249352103	2.909407527	4.86E-05	0.003482824	-0.186927013	2.909407527	0.533316112	1	-0.537141701	2.909407527	0.059928007	0.965078208	cyan
Myb	-1.081372451	4.505107257	0.001334589	0.044095063	-1.689211727	4.505107257	1.26E-05	0.001218188	-0.09944833	4.505107257	0.79233637	1	-0.707283491	4.505107257	0.0463687	0.874164634	cyan
Cpeb1	-0.595856867	3.696546683	0.013842137	0.204558206	-0.890064306	3.696546683	0.001042626	0.036520641	-0.024093847	3.696546683	0.930364028	1	-0.31829844	3.696546683	0.206397971	1	cyan
Blcap	-0.460082163	6.310896759	0.012486022	0.194198129	-0.703317464	6.310896759	0.000611856	0.02500553	-0.003777466	6.310896759	0.98574302	1	-0.247012424	6.310896759	0.191577342	1	cyan
Slc22a3	-0.856556664	4.97860417	0.000783645	0.02960062	-1.456784132	4.97860417	5.03E-07	0.000100426	0.033254399	4.97860417	0.907578309	1	-0.566970944	4.97860417	0.033039931	0.743584658	cyan
Zfp185	-0.519034598	4.879988551	0.005930152	0.122872372	-0.945390314	4.879988551	7.53E-06	0.000822444	0.096588203	4.879988551	0.644937543	1	-0.329766391	4.879988551	0.090272529	1	cyan
Plagl1	-0.480832983	5.290763887	0.009330698	0.161096467	-0.936840254	5.290763887	5.95E-06	0.000676573	0.110880706	5.290763887	0.588777462	1	-0.345125734	5.290763887	0.070151365	1	cyan
Mmab	-0.414635348	4.691837278	0.070155955	0.505697475	-0.835757175	4.691837278	0.001093042	0.037626495	0.110080512	4.691837278	0.665458281	1	-0.311040239	4.691837278	0.188496408	1	cyan
Klhdc7a	-0.486855235	7.438230923	0.051679744	0.434844135	-0.925504453	7.438230923	0.000975529	0.035139157	0.135381978	7.438230923	0.626749124	1	-0.303267071	7.438230923	0.241033178	1	cyan
Mvk	-0.444462441	4.0881636	0.062770875	0.476871702	-0.87403115	4.0881636	0.001069075	0.037167654	0.130764125	4.0881636	0.622220714	1	-0.298802956	4.0881636	0.227181462	1	cyan
Adamts9	-0.273743096	6.166977706	0.093174801	0.566424978	-0.634801923	6.166977706	0.000454845	0.019705984	0.140061733	6.166977706	0.437164417	1	-0.220996862	6.166977706	0.185122249	1	cyan
Morc4	-0.468269168	4.79024127	0.007957546	0.146335078	-0.90627809	4.79024127	4.19E-06	0.000551793	0.170827692	4.79024127	0.380830065	1	-0.267180304	4.79024127	0.141500045	1	cyan
Unc5a	-0.741390854	5.31849252	2.96E-05	0.002413018	-1.370024308	5.31849252	8.51E-12	7.00E-09	0.208559228	5.31849252	0.288765335	1	-0.42007304	5.31849252	0.02227448	0.625116719	cyan
Gbp10	-1.455988444	2.739788442	1.58E-05	0.00159568	-2.464349597	2.739788442	6.57E-10	3.40E-07	0.206445424	2.739788442	0.580622763	1	-0.801895326	2.739788442	0.028228187	0.693510036	cyan
Trhde	-0.831276187	3.238662089	0.009725255	0.166241583	-1.39469895	3.238662089	0.000140771	0.008231871	0.085843861	3.238662089	0.812026861	1	-0.477572807	3.238662089	0.15748689	1	cyan
Cxcl10	-0.972231431	6.784214391	0.000826309	0.030961122	-1.445075983	6.784214391	1.25E-05	0.001218188	0.072929339	6.784214391	0.822218675	1	-0.399914697	6.784214391	0.186263116	1	cyan
Trmt1l	-0.387986321	4.831765766	0.020596316	0.26116258	-0.588865899	4.831765766	0.001564911	0.047858975	0.037060805	4.831765766	0.842209868	1	-0.163818109	4.831765766	0.342415458	1	cyan
Kctd21	-0.884295587	3.768831629	5.68E-05	0.004176837	-1.18319229	3.768831629	1.87E-06	0.000280613	0.01824103	3.768831629	0.942066619	1	-0.280652452	3.768831629	0.223417505	1	cyan
Fbxo45	-0.604443956	5.179256774	0.000154911	0.009413199	-0.777381297	5.179256774	1.29E-05	0.001236006	0.024084917	5.179256774	0.893132047	1	-0.148851742	5.179256774	0.36629954	1	cyan
Rasl12	-0.798746003	4.588594603	0.000392265	0.017799662	-1.181337799	4.588594603	3.29E-06	0.000454905	0.073443451	4.588594603	0.770314848	1	-0.309146594	4.588594603	0.186313018	1	cyan
B3galt2	-0.800525268	7.662657083	0.001793894	0.053917134	-1.165267264	7.662657083	5.64E-05	0.003957778	0.094593976	7.662657083	0.740479087	1	-0.270147829	7.662657083	0.309226141	1	cyan
Dach1	-0.593835747	3.776833438	0.014450198	0.208847955	-0.890469287	3.776833438	0.001062035	0.037014951	0.09489692	3.776833438	0.725663123	1	-0.201734669	3.776833438	0.427122317	1	cyan
Gm4951	-1.19933001	4.255873887	2.40E-05	0.002094958	-1.816492593	4.255873887	2.56E-08	7.94E-06	0.170126582	4.255873887	0.590318072	1	-0.447032208	4.255873887	0.133708432	1	cyan
Nrip1	-0.417638331	6.860399972	0.027077055	0.304203315	-0.693561878	6.860399972	0.000984194	0.035360149	0.11173377	6.860399972	0.593988185	1	-0.16418962	6.860399972	0.397968658	1	cyan
Zeb2	-0.39119775	7.524327573	0.031808906	0.332258053	-0.688665919	7.524327573	0.000687778	0.027077124	0.13090387	7.524327573	0.51688983	1	-0.166564206	7.524327573	0.373004299	1	cyan
Mical3	-0.381414261	6.660872102	0.029057353	0.315299357	-0.62886189	6.660872102	0.001210468	0.039899767	0.112929864	6.660872102	0.55967211	1	-0.134517615	6.660872102	0.453562474	1	cyan
Hykk	-0.691081454	2.852855754	0.007289384	0.139365844	-1.128727582	2.852855754	9.97E-05	0.0063217	0.176653105	2.852855754	0.535434937	1	-0.260988425	2.852855754	0.338171048	1	cyan
Aox1	-0.423592662	5.745520266	0.017585432	0.235415709	-0.664689926	5.745520266	0.000811029	0.03055241	0.138186081	5.745520266	0.484403045	1	-0.102910922	5.745520266	0.576513047	1	cyan
Srf	-0.393516748	6.545497507	0.020792783	0.262058515	-0.635114838	6.545497507	0.000792533	0.030166529	0.13204412	6.545497507	0.483812772	1	-0.109553824	6.545497507	0.531705103	1	cyan
Nlrx1	-0.3949552	4.281665259	0.039890706	0.384562158	-0.673841196	4.281665259	0.00162392	0.049231911	0.148618723	4.281665259	0.485186073	1	-0.130266519	4.281665259	0.514033116	1	cyan
Caprin2	-0.481927541	3.063085597	0.070147804	0.505697475	-0.976082713	3.063085597	0.00107643	0.037238092	0.280346225	3.063085597	0.342356995	1	-0.21380648	3.063085597	0.447459802	1	cyan
Tra2a	-0.341099415	5.799573537	0.044852114	0.409116592	-0.662574034	5.799573537	0.00045553	0.019705984	0.189754755	5.799573537	0.313123762	1	-0.131719645	5.799573537	0.451352548	1	cyan
Ccr10	-0.794372408	0.491421989	0.025769337	0.296349735	-1.453367311	0.491421989	0.000342293	0.016220179	0.379749328	0.491421989	0.325355758	1	-0.279219087	0.491421989	0.497471154	1	cyan
Hoxc6	-0.535512876	4.222381912	0.008668573	0.154727945	-0.997856049	4.222381912	1.19E-05	0.00118296	0.293120227	4.222381912	0.193279118	1	-0.169221924	4.222381912	0.423619053	1	cyan
Efna5	-0.350053342	4.247581332	0.074183331	0.516585071	-0.822767434	4.247581332	0.00015811	0.008874495	0.420997265	4.247581332	0.051687895	0.715274536	-0.051716688	4.247581332	0.804977708	1	cyan
Hsph1	-0.594796213	5.788931896	0.005477559	0.116521108	-1.409570039	5.788931896	5.75E-09	2.17E-06	0.616771004	5.788931896	0.009600531	0.331452611	-0.198002484	5.788931896	0.371143888	1	cyan
Gbp2	-0.252387193	6.511745738	0.219731189	0.765683478	-0.811516183	6.511745738	0.000406546	0.018211169	0.405528091	6.511745738	0.075690748	0.817704713	-0.153600831	6.511745738	0.468811914	1	cyan
Gnptab	-0.166833277	6.362024217	0.342785305	0.863177667	-0.629942984	6.362024217	0.001272972	0.041466688	0.308796061	6.362024217	0.113012421	0.928722145	-0.15431358	6.362024217	0.392406849	1	cyan
Slc16a5	-1.486137879	1.54565587	0.001199939	0.040508089	-3.482932024	1.54565587	6.52E-10	3.40E-07	1.123691881	1.54565587	0.02676784	0.550157848	-0.873037136	1.54565587	0.095203645	1	cyan
Abcd2	-0.415574495	8.321498391	0.095176744	0.573017654	-0.992500237	8.321498391	0.000386998	0.01756068	0.351866968	8.321498391	0.20449533	1	-0.225058718	8.321498391	0.382459824	1	cyan
Unc119	-0.361841735	4.957295904	0.10160579	0.589718653	-0.900508656	4.957295904	0.000265712	0.013358216	0.291600837	4.957295904	0.233911128	1	-0.247065491	4.957295904	0.278529153	1	cyan
Sntb1	-0.31524821	5.417668277	0.143913981	0.671342389	-0.834471794	5.417668277	0.000529365	0.022342528	0.303552768	5.417668277	0.204483113	1	-0.215670478	5.417668277	0.332082937	1	cyan
Pnpla3	-0.52822955	7.752685298	0.105540189	0.596453572	-1.397060944	7.752685298	0.000169038	0.009412245	0.472810676	7.752685298	0.195612	1	-0.396020563	7.752685298	0.245044588	1	cyan
Cldn8	-0.623107851	4.409060495	0.001362703	0.044602187	-1.747477339	4.409060495	2.89E-15	6.72E-12	0.611813284	4.409060495	0.004435937	0.212317292	-0.512553981	4.409060495	0.011522321	0.434398616	cyan
Mknk2	-0.300444678	9.444750595	0.149580381	0.679962827	-0.793982865	9.444750595	0.000649788	0.026171275	0.26543824	9.444750595	0.251658519	1	-0.228099925	9.444750595	0.287325899	1	cyan
Zfp467	-0.225657879	4.804509838	0.200456576	0.749423702	-0.634761966	4.804509838	0.001173604	0.03946393	0.192151907	4.804509838	0.323539266	1	-0.216951685	4.804509838	0.230103595	1	cyan
Dmpk	-0.320257592	6.248253207	0.156726734	0.691243258	-0.934233098	6.248253207	0.000225406	0.011885403	0.274709684	6.248253207	0.274192651	1	-0.339265513	6.248253207	0.145675073	1	cyan
Sfpq	-0.145487549	7.339392446	0.396338437	0.890020106	-0.601773024	7.339392446	0.00162285	0.049231911	0.24609341	7.339392446	0.196020637	1	-0.210192006	7.339392446	0.232019705	1	cyan
Serpina1c	-0.657646668	-0.623565762	0.226986649	0.771865062	-2.754000115	-0.623565762	2.05E-05	0.001779052	0.832636612	-0.623565762	0.146671622	0.994029995	-1.263475074	-0.623565762	0.044160047	0.858387787	cyan
Cdsn	-0.207113514	5.68933344	0.493575452	0.930813487	-1.140083378	5.68933344	0.000917081	0.033818261	0.42939961	5.68933344	0.206777794	1	-0.503569702	5.68933344	0.112598815	1	cyan
Esrrb	-0.193399859	2.921335333	0.727291005	0.981189896	-2.826909521	2.921335333	6.79E-05	0.00463165	1.054094794	2.921335333	0.114294559	0.933150299	-1.579394136	2.921335333	0.014911044	0.498843884	cyan
Mir6392	-0.154735294	0.165952071	0.743352995	0.982603121	-2.633240362	0.165952071	3.88E-06	0.000516465	0.851586438	0.165952071	0.100893369	0.900794776	-1.626776479	0.165952071	0.002654037	0.179192371	cyan
Parp3	-0.304925167	6.160294502	0.147054715	0.676064703	-0.892558446	6.160294502	0.000147819	0.00848783	0.093731302	6.160294502	0.688062046	1	-0.49390146	6.160294502	0.022859698	0.628715895	cyan
Dclk1	-0.458693167	4.596638046	0.048687307	0.424752689	-1.36325766	4.596638046	2.31E-07	5.22E-05	0.037738425	4.596638046	0.88519321	1	-0.86682296	4.596638046	0.000383748	0.043575463	cyan
Slc2a13	-0.438599626	4.151513304	0.140038009	0.664574266	-1.143671529	4.151513304	0.000666053	0.026520674	-0.023926832	4.151513304	0.943274952	1	-0.728995214	4.151513304	0.019378008	0.570162176	cyan
Tshr	-0.626089173	6.302974488	0.014040854	0.204766082	-1.403974445	6.302974488	1.21E-06	0.000199416	-0.087710223	6.302974488	0.757316164	1	-0.865594425	6.302974488	0.00119644	0.103859953	cyan
Rbm14	-0.27321298	4.903956848	0.093074536	0.566061668	-0.66109674	4.903956848	0.000258644	0.013106377	0.025418606	4.903956848	0.88895805	1	-0.362464215	4.903956848	0.030071027	0.712887181	cyan
Slc18b1	-0.376743982	5.021003054	0.084030241	0.539791925	-0.837792284	5.021003054	0.000587619	0.024297523	0.050283777	5.021003054	0.835903501	1	-0.410763463	5.021003054	0.068020809	1	cyan
Hoxc8	-0.362211326	5.367955282	0.072616115	0.514592937	-0.722971049	5.367955282	0.00130796	0.042008616	0.042540306	5.367955282	0.849935504	1	-0.318218744	5.367955282	0.125257652	1	cyan

Rgs7bp	-0.580934416	2.884841897	0.063907776	0.480977428	-1.14275987	2.884841897	0.001299105	0.041910457	0.013903506	2.884841897	0.971363358	1	-0.547914721	2.884841897	0.096051431	1	cyan
Apcdd1	-0.572287611	6.703558939	0.010105312	0.170982853	-0.987020744	6.703558939	7.74E-05	0.005150196	-0.092343113	6.703558939	0.708787753	1	-0.507075757	6.703558939	0.027599856	0.687550018	cyan
Ephb2	-0.848049728	3.907302519	0.003835427	0.091944986	-1.30296513	3.907302519	9.35E-05	0.006022318	-0.184057221	3.907302519	0.573266335	1	-0.638967633	3.907302519	0.038091425	0.794575765	cyan
Lhfpl2	-0.455209617	5.996942478	0.022659267	0.274106207	-0.760012461	5.996942478	0.000655449	0.02624802	-0.102083196	5.996942478	0.645098129	1	-0.40688542	5.996942478	0.048034353	0.884490277	cyan
Fam20c	-0.703526453	3.348807939	0.002533336	0.068883094	-1.177484616	3.348807939	8.67E-06	0.000903978	-0.196383971	3.348807939	0.447534292	1	-0.670335135	3.348807939	0.006366786	0.30702712	cyan
Rtp4	-0.405201454	5.296288953	0.025961753	0.296924269	-0.65815673	5.296288953	0.001174656	0.03946393	-0.120904335	5.296288953	0.549439109	1	-0.373858691	5.296288953	0.045947244	0.874164634	cyan
Idh1	-0.707270376	7.961231696	0.00648504	0.130597857	-0.998953804	7.961231696	0.000629716	0.025509877	-0.270982396	7.961231696	0.349271244	1	-0.56266557	7.961231696	0.037085312	0.784121507	cyan
Dtx2	-0.758039033	5.2408423	1.85E-05	0.001783651	-0.859080346	5.2408423	1.55E-05	0.00143999	-0.402513517	5.2408423	0.040570113	0.661619478	-0.50355318	5.2408423	0.006092689	0.303004374	cyan
Phkg1	-1.090425922	3.472813964	0.005703442	0.120409828	-1.485369435	3.472813964	0.001061267	0.037014951	-0.690571382	3.472813964	0.118822329	0.946501935	-1.085502413	3.472813964	0.010487921	0.406036508	cyan
Trabd2b	-0.875841622	6.195963414	0.002821159	0.075245257	-1.51644741	6.195963414	5.82E-06	0.000676573	-0.347292674	6.195963414	0.288699196	1	-0.98789693	6.195963414	0.001420434	0.117467379	cyan
Cmklr1	-0.560996948	6.852962702	0.009187507	0.159707212	-1.113054462	6.852962702	4.36E-06	0.000557251	-0.167604346	6.852962702	0.483546366	1	-0.719661265	6.852962702	0.001312615	0.112546638	cyan
Igfals	-0.889737669	4.903258112	0.004975586	0.108919069	-1.672152154	4.903258112	4.25E-06	0.000555396	-0.315090071	4.903258112	0.372913157	1	-1.097500347	4.903258112	0.001123865	0.100686796	cyan
Ffar2	-0.598681365	4.829452698	0.00683179	0.134860315	-1.081708478	4.829452698	1.48E-05	0.001392839	-0.343799145	4.829452698	0.162115973	1	-0.826823375	4.829452698	0.000363376	0.042950711	cyan
Mcam	-0.539167949	7.890879587	0.009032817	0.15819881	-1.013276253	7.890879587	1.23E-05	0.001210891	-0.324702596	7.890879587	0.157181396	1	-0.798810576	7.890879587	0.000203759	0.027121311	cyan
Tenm4	-0.728837038	7.430054636	0.03912539	0.380791405	-1.425073817	7.430054636	0.00040506	0.018211169	-0.35241304	7.430054636	0.372965583	1	-1.048649178	7.430054636	0.00516908	0.273341102	cyan
Serpina3c	-0.470897679	6.650315254	0.117680028	0.622755045	-1.078277209	6.650315254	0.001505391	0.046381353	-0.155172946	6.650315254	0.644116596	1	-0.762551795	6.650315254	0.015625393	0.50904544	cyan
Peg10	-0.267730781	3.893054777	0.181524208	0.728300217	-0.749934482	3.893054777	0.000834931	0.0312612	-0.088436756	3.893054777	0.691626232	1	-0.570637466	3.893054777	0.006113855	0.303004374	cyan
Irs3	-0.127886354	4.635216326	0.60530965	0.96149007	-1.413725178	4.635216326	5.87E-07	0.000112399	0.239865418	4.635216326	0.386876573	1	-1.045970459	4.635216326	7.05E-05	0.011330343	cyan
Serpina3n	-0.131557939	7.68672432	0.670560717	0.973876581	-1.182494955	7.68672432	0.000925469	0.033858505	-0.064745436	7.68672432	0.854190483	1	-1.11568205	7.68672432	0.000855734	0.080319634	cyan
Sgms2	-0.06643689	5.783369542	0.782762078	0.987952495	-0.936190191	5.783369542	0.000757848	0.029147023	-0.001496532	5.783369542	0.996484398	1	-0.871248786	5.783369542	0.000774757	0.076253534	cyan
Sncg	0.281533027	7.679267496	0.357849641	0.873590669	-1.023945657	7.679267496	0.002025407	0.057301809	0.089752657	7.679267496	0.784695865	1	-1.215725689	7.679267496	0.000102628	0.015258823	cyan
Prr32	1.064190869	0.929111753	0.079406966	0.529953489	-1.084198716	0.929111753	0.112426707	0.536821202	-0.460636174	0.929111753	0.479905519	1	-2.608917261	0.929111753	6.54E-05	0.010630753	firebrick1
Timp1	0.423590599	3.274513317	0.260756957	0.805555578	-0.47982719	3.274513317	0.240395382	0.720200554	-0.546804817	3.274513317	0.177135114	1	-1.450212445	3.274513317	0.000193009	0.025937381	firebrick1
Fam83e	0.356579147	2.160952793	0.449684531	0.912511115	-1.075733015	2.160952793	0.039568824	0.324357628	-1.216053376	2.160952793	0.017282202	0.445638474	-2.64830305	2.160952793	3.05E-07	0.000125553	firebrick1
Creb3l1	0.08235482	5.3306639	0.678948305	0.973876581	-0.478847283	5.3306639	0.025409345	0.251502131	-0.378555099	5.3306639	0.076071952	0.818134822	-0.939755591	5.3306639	2.80E-06	0.000849798	firebrick1
Spon1	-0.075600221	7.5358696	0.776741352	0.987066641	-0.610504974	7.5358696	0.047333461	0.357507289	-0.72320192	7.5358696	0.018881872	0.461913105	-1.258106107	7.5358696	1.71E-05	0.003803256	firebrick1
Agt	0.06164286	4.910788222	0.826174714	0.993891716	-0.811859501	4.910788222	0.005627304	0.109996092	-0.54195502	4.910788222	0.062359319	0.771874186	-1.41545373	4.910788222	3.80E-07	0.000151715	firebrick1
3830417A13Rik	0.105994432	0.837694188	0.870240614	1	-1.382885782	0.837694188	0.02760013	0.263303353	-0.77058486	0.837694188	0.194428047	1	-2.259340338	0.837694188	0.000146936	0.020535843	firebrick1
Fgf10	0.039297288	6.27743709	0.813469785	0.992237189	-0.427674129	6.27743709	0.015741253	0.19566743	-0.150194903	6.27743709	0.395302222	1	-0.617165827	6.27743709	0.000173489	0.023771338	firebrick1
Cfb	-0.115174903	7.317063723	0.593491171	0.95880622	-0.683294157	7.317063723	0.004889993	0.101376359	-0.318202951	7.317063723	0.18812539	1	-0.886321787	7.317063723	9.54E-05	0.014658159	firebrick1
Serpinh1	-0.183624079	9.119820877	0.346391208	0.866251564	-0.634095457	9.119820877	0.003555326	0.081860357	-0.276092949	9.119820877	0.202796282	1	-0.726564226	9.119820877	0.000333396	0.040517746	firebrick1
Ttyh2	-0.341436666	6.115326008	0.061294981	0.473815629	-0.573696169	6.115326008	0.004821415	0.10042361	-0.598432392	6.115326008	0.003188367	0.17636499	-0.83069079	6.115326008	1.23E-05	0.00295713	firebrick1
C2	-0.230859588	6.19835561	0.185627906	0.730456392	-0.498956288	6.19835561	0.010223779	0.15251593	-0.510505246	6.19835561	0.008400237	0.306129109	-0.778601035	6.19835561	1.71E-05	0.003803256	firebrick1
C4b	-0.264366688	8.982150868	0.156915229	0.691376808	-0.731506398	8.982150868	0.000443813	0.019383518	-0.74828335	8.982150868	0.000325202	0.037254236	-1.215422845	8.982150868	6.94E-10	4.41E-07	firebrick1
Chrdl1	-0.289334016	5.945803862	0.063272794	0.47800031	-0.370003339	5.945803862	0.033111612	0.293926096	-0.705980766	5.945803862	4.64E-05	0.009682532	-0.786648887	5.945803862	1.16E-06	0.000405646	firebrick1
Gas6	-1.515651368	8.666581017	0.000370262	0.017135018	-0.500421971	8.666581017	0.293923568	0.765012213	-1.801369159	8.666581017	0.000236673	0.031357795	-0.786139399	8.666581017	0.079942195	1	firebrick1
Efhd1	-0.942398546	3.912798826	0.000670159	0.026914212	-0.288065495	3.912798826	0.355568554	0.812794589	-1.002934293	3.912798826	0.001298086	0.100232328	-0.348596615	3.912798826	0.229076898	1	firebrick1
Ldb3	-2.135048925	3.528305598	9.53E-06	0.001148677	-0.401991226	3.528305598	0.46204131	0.877383718	-2.459592694	3.528305598	1.32E-05	0.003761765	-0.726517926	3.528305598	0.154335	1	firebrick1
Lgi2	-1.16338824	2.886391564	0.002273022	0.063535518	-0.267198133	2.886391564	0.543875861	0.905060463	-1.601202525	2.886391564	0.000253866	0.031964298	-0.704996102	2.886391564	0.081971	1	firebrick1
Cd209g	-1.417734235	3.058817124	0.000109185	0.007281448	-0.144481775	3.058817124	0.72994669	0.965045279	-1.720715953	3.058817124	4.42E-05	0.009384939	-0.447450139	3.058817124	0.251593984	1	firebrick1
Ivl	-4.200360847	-0.978687181	1.72E-05	0.001708203	1.565861032	-0.978687181	0.285413102	0.756852872	-5.486450848	-0.978687181	1.87E-05	0.004942492	0.281045452	-0.978687181	0.850866969	1	firebrick1
Dab2	-0.764236601	6.309940035	0.000187387	0.010600388	0.037286002	6.309940035	0.870916255	0.988639654	-0.901084939	6.309940035	8.49E-05	0.01540585	-0.099561733	6.309940035	0.64022988	1	firebrick1
Ngfr	-1.86995823	3.82443267	5.85E-05	0.004234773	0.293803325	3.82443267	0.575118167	0.914616378	-2.44864968	3.82443267	7.51E-06	0.002282734	-0.284878116	3.82443267	0.565885984	1	firebrick1
Arsi	-1.222267311	2.644622585	0.001730217	0.052227904	0.185883296	2.644622585	0.682008694	0.952820984	-1.663751506	2.644622585	0.000203887	0.028783008	-0.255586274	2.644622585	0.540514148	1	firebrick1
Chsy3	-1.010990275	2.089708637	0.004751895	0.105416649	0.312349504	2.089708637	0.444837192	0.868423605	-1.448212634	2.089708637	0.000413163	0.045828357	-0.124855857	2.089708637	0.758720706	1	firebrick1
Cldn11	-1.812953211	0.082820914	0.00557761	0.118289341	0.446543952	0.082820914	0.618678693	0.929258879	-3.002952381	0.082820914	0.000175938	0.025883311	-0.743243085	0.082820914	0.308038647	1	firebrick1
Hif1a	-0.582153085	7.897680488	0.004350517	0.099676769	0.06919138	7.897680488	0.760673163	0.968583101	-0.873066677	7.897680488	0.00013266	0.02098527	-0.221722001	7.897680488	0.291295246	1	firebrick1
Sprr1a	-1.351369007	2.94260577	0.000353761	0.016602381	0.329426328	2.94260577	0.447494365	0.869239922	-2.109977768	2.94260577	1.76E-06	0.000767591	-0.429166138	2.94260577	0.286261942	1	firebrick1
Gsr	-0.521862224	6.406781314	0.007023971	0.136911847	0.279566642	6.406781314	0.194962101	0.667601274	-1.028805985	6.406781314	2.17E-06	0.000864821	-0.227376469	6.406781314	0.253367533	1	firebrick1
Mrc1	-0.436612215	6.894775325	0.101433693	0.589454178	0.32803339	6.894775325	0.27034355	0.742594625	-1.087644184	6.894775325	0.000293004	0.035573925	-0.322998065	6.894775325	0.242228823	1	firebrick1
Gm14005	-0.785642145	2.363416587	0.02763947	0.308047179	0.750684986	2.363416587	0.072426925	0.44029522	-2.31424186	2.363416587	4.39E-08	2.56E-05	-0.777884471	2.363416587	0.040261937	0.814861989	firebrick1
Mgl2	-0.333659808	5.637249091	0.228482335	0.773020276	0.311371794	5.637249091	0.315934783	0.785073719	-1.130553228	5.637249091	0.000304339	0.03604613	-0.485520218	5.637249091	0.092017363	1	firebrick1
Areg	-0.396601574	8.008953601	0.225939719	0.771815654	0.469824741	8.008953601	0.201122076	0.674853955	-1.439902293	8.008953601	0.000117401	0.019079065	-0.573475632	8.008953601	0.094498889	1	firebrick1
Thsd4	-0.293793243	5.773376075	0.249789364	0.794011058	0.300261558	5.773376075	0.293163588	0.764413118	-1.18859181	5.773376075	3.76E-05	0.008765327	-0.594535583	5.773376075	0.025124468	0.66252748	firebrick1
Siglec1	-0.583887758	5.675613489	0.045613251	0.413365976	0.069986903	5.675613489	0.831721104	0.980677001	-1.384942475	5.675613489	3.01E-05	0.007374637	-0.731065866	5.675613489	0.016821808	0.527550793	firebrick1
Wfdc17	-0.737419667	2.638940486	0.051970475	0.435078428	0.081903654	2.638940486	0.856229639	0.984992665	-1.569628381	2.638940486	0.000323633	0.037254236	-0.750287053	2.638940486	0.063697495	0.998022638	firebrick1
Sfrp4	-0.743758294	4.027944069	0.022451723	0.273332127	0.128989531	4.027944069	0.728428748	0.965045279	-1.409626606	4.027944069	0.000146274	0.022465104	-0.536873206	4.027944069	0.116168774	1	firebrick1
Cd209f	-1.031620784	4.494775155	0.001634469	0.050252623	0.05674995	4.494775155	0.880696438	0.990473438	-1.772960862	4.494775155	2.54E-06	0.0009583	-0.684584732	4.494775155	0.046827559	0.874949149	firebrick1
Fcgr1	-0.972470036	3.003002523	0.008219482	0.14895629	-0.079591055	3.003002523	0.854434111	0.984607393	-1.55764938	3.003002523	0.000225752	0.030632082	-0.664756602	3.003002523	0.087754348	1	firebrick1
Npl	-0.80837096	2.415136742	0.012155313	0.191569999	-0.071160492	2.415136742	0.86029913	0.986263689	-1.378014503	2.415136742	0.000179501	0.026132408	-0.640785549	2.415136742	0.062047622	0.979861648	firebrick1
Bst1	-0.484426706	2.103957866	0.152952593	0.683983455	-0.004699776	2.103957866	1	1	-1.432064325	2.103957866	0.000236481	0.031357795	-0.952310362	2.103957866	0.008738408	0.369985586	firebrick1
Ccl8	-0.481789919	4.040652834	0.109336056	0.604462311	0.114887395	4.040652834	0.738679744	0.965045279	-1.408776064	4.040652834	3.95E-05	0.008958835	-0.812092415	4.040652834	0.01035029	0.404698347	firebrick1
Tmed3	-0.441604626	7.50068509	0.109930592	0.605717291	0.151031575	7.50068509	0.624542675	0.931608926	-1.466417224	7.50068509	3.07E-06	0.001071415	-0.873780415	7.50068509	0.002570053	0.176823703	firebrick1
Tcf21	-0.897636317	-0.640277544	0.28765791	0.824970472	1.576613091	-0.640277544	0.43940259	0.865305943	-6.148430213	-0.640277544	7.22E-06	0.002243028	-3.671029552	-0.640277544	0.001363224	0.114862431	firebrick1
Rnase1	-0.393776259	-1.192340747	0.454152188	0.913501714	-1.211636258	-1.192340747	0.267312126	0.740071375	-3.006132343	-1.192340747	2.10E-05	0.005422402	-3.821955711	-1.192340747	1.25E-06	0.000424439	firebrick1
Vnn1	-0.120962563	3.123880895	0.687378612	0.974759724	-0.210391396	3.123880895	0.551986536	0.907049956	-1.224538549	3.123880895	0.000428109	0.046381842	-1.313952772	3.123880895	6.50E-05	0.010630753	firebrick1
Padi4	-0.318888633	3.802089597	0.560699276	0.946944793	0.145239522	3.802089597	0.831885058	0.980677001	-3.148547478	3.802089597	5.13E-06	0.001667822	-2.684386379	3.802089597	3.86E-05	0.007388841	firebrick1
Muc13	-0.061519007	4.817661121	0.896040214	1	0.460560857	4.817661121	0.485338478	0.885297579	-2.724055014	4.817661121	8.95E-05	0.015442991	-2.201963688	4.817661121	0.000734623	0.072816296	firebrick1
Upk3b	0.842594239	-0.525561475	0.453994862	0.913501714	0	-0.525561475	1	1	-8.389166747	-0.525561475	4.97E-05	0.010060595	-9.183459255	-0.525561475	5.27E-06	0.00150266	firebrick1
Aoc1	-0.086140421	4.821036587	0.880826855	1	2.501946478	4.821036587	0.004457223	0.096281531	-6.05834159	4.821036587	2.86E-09	2.67E-06	-3.470183005	4.821036587	7.30E-05	0.011599423	darkgreen
Ms4a6d	-0.036717769	3.348371754	0.89770714	1	0.729990736	3.348371754	0.038116833	0.316907112	-1.339745054	3.348371754	0.000151316	0.022812507	-0.573028565	3.348371754	0.076470564	1	darkgreen
Krt4	0.147921623	1.746538842	0.817211532	0.992918622	1.666780548	1.746538842	0.012401475	0.171606939	-2.961256183	1.746538842	1.70E-05	0.004646006	-1.442311188	1.746538842	0.018500519	0.554856774	darkgreen
Wfdc3	0.191486819	2.318550889	0.719750559	0.980142426	1.423872467	2.318550889	0.012796244	0.173692314	-2.805459257	2.318550889	1.94E-06	0.000797648	-1.573018296	2.318550889	0.002998054	0.193091276	darkgreen
Faim2	0.762081593	4.193802732	0.033543188	0.345213255	1.100025393	4.193802732	0.004932506	0.101701826	-1.85608005	4.193802732	2.97E-06	0.001070258	-1.518126674	4.193802732	3.81E-05	0.007388841	darkgreen
Slco2a1	0.273913782	7.196662291	0.190754088	0.737272992	0.686659284	7.196662291	0.002545457	0.06554194	-0.932332497	7.196662291	4.43E-05	0.009384939	-0.519586622	7.196662291	0.013450854	0.479564123	darkgreen
Greb1	0.36223352	2.008932254	0.632585788	0.969614941	2.5000041	2.008932254	0.002830983	0.070653258	-3.423758012	2.008932254	7.38E-05	0.013772289	-1.285890022	2.008932254	0.085207477	1	darkgreen
Gng12	0.098171432	8.312034352	0.579032882	0.953302094	0.43673505	8.312034352	0.022296168	0.234494547	-0.730959137	8.312034352	0.000137139	0.02129612	-0.392395386	8.312034352	0.026160109	0.680844858	darkgreen
Tmem176a	0.064241324	6.834487605	0.75330096	0.983951948	0.44949169	6.834487605	0.039092435	0.322333848	-0.765531655	6.834487605	0.000459041	0.049350387	-0.380280905	6.834487605	0.058666146	0.962015487	darkgreen
Tmem86a	0.146162463	5.704651744	0.685045723	0.974473184	1.466720838	5.704651744	0.000152057	0.008603816	-2.10766784	5.704651744	9.10E-08	4.89E-05	-0.787106644	5.704651744	0.027012182	0.685465124	darkgreen
Tgm2	0.01262139	10.88713806	0.973285771	1	1.171208983	10.88713806	0.000115655	0.007058465	-1.781767431	10.88713806	7.93E-09	6.15E-06	-0.623179779	10.88713806	0.026042599	0.680320318	darkgreen
Ptpn5	-0.235956815	4.230415661	0.705737548	0.976863224	4.103681985	4.230415661	2.07E-06	0.000305062	-5.637865185	4.230415661	1.07E-09	1.15E-06	-1.298156189	4.230415661	0.074419426	1	darkgreen
Fbln1	0.143915463	5.870305998	0.454000037	0.913501714	0.768753438	5.870305998	0.00023658	0.012200885	-0.838541057	5.870305998	6.13E-05	0.012075656	-0.213702433	5.870305998	0.265194089	1	darkgreen
Ccl9	0.237840072	6.104432277	0.229432481	0.773780974	1.032461712	6.104432277	1.90E-06	0.000282562	-1.083473301	6.104432277	5.87E-07	0.000282839	-0.288850895	6.104432277	0.144437826	1	darkgreen
Ms4a6c	0.260763736	4.307117619	0.294755649	0.82920792	0.898251365	4.307117619	0.000939043	0.034102749	-0.907500429	4.307117619	0.000830384	0.073452215	-0.27001065	4.307117619	0.278150526	1	darkgreen
Ccl6	0.366847287	7.004637986	0.156143983	0.691028594	1.463337241	7.004637986	2.69E-07	5.87E-05	-1.32452912	7.004637986	2.99E-06	0.001070258	-0.228038657	7.004637986	0.378559992	1	darkgreen
Ninj1	0.049447597	5.327904469	0.781634129	0.987815684	0.644289137	5.327904469	0.000701049	0.027368342	-0.685574694	5.327904469	0.000310704	0.036490767	-0.090732475	5.327904469	0.605824221	1	darkgreen
Fcgr2b	0.015907523	4.974415883	0.950173218	1	0.844991536	4.974415883	0.000378581	0.017358896	-0.941265071	4.974415883	7.61E-05	0.013995466	-0.112179788	4.974415883	0.61019141	1	darkgreen
Alox5ap	-0.047317666	4.212379709	0.851088043	0.997576004	0.942049416	4.212379709	0.001497897	0.046381353	-1.09668701	4.212379709	0.000223694	0.030632082	-0.107317351	4.212379709	0.699040334	1	darkgreen
Thbs2	-0.116075446	7.509686801	0.537374786	0.940321774	0.792033458	7.509686801	0.000175775	0.009709993	-0.838091879	7.509686801	7.27E-05	0.013772289	0.070017184	7.509686801	0.707594879	1	darkgreen
Mcpt4	-0.355850975	4.116096427	0.203468716	0.751702402	0.864343357	4.116096427	0.006123861	0.115346467	-1.128874528	4.116096427	0.00035749	0.040620217	0.091322285	4.116096427	0.739716345	1	darkgreen
Slpi	-0.442712874	3.731087085	0.040207194	0.385415463	1.067389147	3.731087085	1.26E-05	0.001218188	-1.357610601	3.731087085	3.18E-08	2.02E-05	0.152495351	3.731087085	0.481163298	1	darkgreen
Matn4	-0.594291137	1.411456086	0.069217501	0.503115788	0.869232935	1.411456086	0.021107454	0.226634864	-1.359508586	1.411456086	0.000292223	0.035573925	0.104036121	1.411456086	0.756918236	1	darkgreen
Dscam	-1.164402953	2.357968295	0.055932769	0.451044994	1.695222336	2.357968295	0.017445552	0.206626299	-2.589229735	2.357968295	0.000399548	0.044672631	0.270419827	2.357968295	0.648934735	1	darkgreen
Mmp9	-0.757380069	3.600051917	0.01469097	0.211018493	0.775307338	3.600051917	0.027349474	0.261773795	-1.386235032	3.600051917	8.87E-05	0.015442991	0.146456862	3.600051917	0.634555222	1	darkgreen
Clec3b	-0.390876516	6.729961858	0.053938011	0.441874348	0.569218735	6.729961858	0.01179628	0.166362066	-0.943714819	6.729961858	3.24E-05	0.007812573	0.016380794	6.729961858	0.929290544	1	darkgreen
Cystm1	-0.256440791	3.932157711	0.188896484	0.73476684	0.416057914	3.932157711	0.057316121	0.392287027	-0.789965797	3.932157711	0.000296682	0.035573925	-0.117464664	3.932157711	0.562548133	1	darkgreen
Nfam1	-0.42304073	3.408923677	0.132211096	0.65130045	0.500768998	3.408923677	0.114956193	0.540654648	-1.166634641	3.408923677	0.00025059	0.031964298	-0.242819246	3.408923677	0.409142553	1	darkgreen
Fcrls	-0.283048288	4.602875016	0.200887366	0.749441765	0.391680082	4.602875016	0.114169965	0.5394234	-0.975355687	4.602875016	8.67E-05	0.015442991	-0.300625117	4.602875016	0.188209176	1	darkgreen
Itgam	-0.192638206	5.109687116	0.479542932	0.924624379	0.560162727	5.109687116	0.068525004	0.428120455	-1.229005759	5.109687116	7.37E-05	0.013772289	-0.476202741	5.109687116	0.094224904	1	darkgreen
Cfp	-0.13919803	5.621597244	0.599021995	0.960083876	0.579291207	5.621597244	0.053314878	0.379478602	-1.143986146	5.621597244	0.0001518	0.022812507	-0.425495589	5.621597244	0.125025226	1	darkgreen
Gm13031	-0.335201552	2.070189347	0.369061462	0.880194409	1.040788616	2.070189347	0.017899307	0.209630678	-1.961563591	2.070189347	9.28E-06	0.002758403	-0.58554468	2.070189347	0.142100715	1	darkgreen
Spi1	-0.1770576	3.787691061	0.495308868	0.931687313	0.55394869	3.787691061	0.05987238	0.400346125	-1.066066032	3.787691061	0.000297807	0.035573925	-0.335055572	3.787691061	0.215705241	1	darkgreen
Padi2	-0.351820167	5.687445826	0.218911486	0.764384385	1.39749848	5.687445826	1.88E-05	0.001663136	-2.235978296	5.687445826	2.30E-11	2.93E-08	-0.486657004	5.687445826	0.102403218	1	darkgreen
Aqp3	-0.416575482	0.973379448	0.31895265	0.847629252	1.431905002	0.973379448	0.003850706	0.086107947	-2.178865286	0.973379448	1.22E-05	0.003554766	-0.330320801	0.973379448	0.46124331	1	darkgreen

Supplementary Table 2. Oligonucleotide sequences used in this manuscript.

Hairpin	shDnmt1	ACCAAGCTGTGTAGTACTTTG
Hairpin	shDnm3a	CGCTCCGCTGAAGGAATATTT
Hairpin	shDnmt3b	GCACTTTAATCTGGCTACCTT
Hairpin	shTet1	AATAGAGGATTACTAAGCAAG
Hairpin	shTet2	GAGCGTTCCTCAGTATCATTT
Hairpin	shTet3	GCTCCAACGAGAAGCTATTTG
gRNA	gHdac1 (#2)	CACCGAATCCGCATGACTCA
Q-PCR	Cyclophilin_F	GGTGGAGAGCACCAAGACAGA
Q-PCR	Cyclophilin_R	GCCGGAAGTCGACAATGATG
Q-PCR	Tet1-f	CCCAGACTCCTTAACTTGCA
Q-PCR	Tet1-r	CTCGTCCTGGATATTATGTGTAC
Q-PCR	Tet2-f	AGAGCCTCAAGCAACCAAAA
Q-PCR	Tet2-r	ACATCCCTGAGAGCTCTTGC
Q-PCR	Tet3-f	CCGGATTGAGAAGGTCATCTAC
Q-PCR	Tet3-r	AAGATAACAATCACGGCGTTCT
Q-PCR	Ppargc1a_f	AGCCGTGACCACTGACAACGAG
Q-PCR	Ppargc1a_r	GCTGCATGGTTCTGAGTGCTAAG
Q-PCR	Ucp1_f	CACCTTCCCGCTGGACACT
Q-PCR	Ucp1_r	CCCTAGGACACCTTTATACCTAATGG
Q-PCR	Elovl3_f	TCCGCGTTCTCATGTAGGTCT
Q-PCR	Elovl3_r	GGACCTGATGCAACCCTATGA
Q-PCR	Fabp4_f	AAGGTGAAGAGCATCATAACCCT
Q-PCR	Fabp4_r	TCACGCCTTTCATAACACATTCC
Q-PCR	Pparg_f	CAAGAATACCAAAGTGCGATCAA
Q-PCR	Pparg_r	GAGCTGGGTCTTTTCAGAATAATAAG
Q-PCR	Ppara_f	GCCTGTCTGTCGGGATGT
Q-PCR	Ppara_r	GGCTTCGTGGATTCTCTTG
ChIP	Ucp1_promoter_f	CCCACTAGCAGCTCTTTGGA
ChIP	Ucp1_promoter_r	CTGTGGAGCAGCTCAAAGGT
ChIP	Ucp1_enhancer_f	CTCCTCTACAGCGTCACAGAGG
ChIP	Ucp1_enhancer_r	AGTCTGAGGAAAGGGTTGA
ChIP	Ppargc1a_promoter_f	CAAAGCTGGCTTCAGTCACA
ChIP	Ppargc1a_promoter_r	AAAAGTAGGCTGGGCTGTCA
ChIP	Ins_f	GGACCCACAAGTGGAACAAC
ChIP	Ins_r	GTGCAGCACTGATCCACAAT
Bisulfite	Ucp1 (#1)_f	TAAGGGTTGGTTTATGAGTTTAGTTG
Bisulfite	Ucp1 (#1)_r	TTCAAATATCACCTTCAAATTTAAATAACT
Bisulfite	Ucp1 (#2)_f	TTTTGAGAGAAATTATGGGAATTAAAA
Bisulfite	Ucp1_(#2)_r	CATAACCCCAAAACTACAAAAAATAAC
Bisulfite	Ucp1 (#3)_f	AGTTAGGTTGGGTTGTATATTTTTGT
Bisulfite	Ucp1 (#3)_r	TTACTTTTCAAACTTTCTTACACTTTTAAA
Bisulfite	Ucp1 (#4)_f	TTTTTTTGGAGATAGATAAGAAGTTA
Bisulfite	Ucp1_(#4)_r	AAAATATAAAACACCATTTACAAAACAC
Bisulfite	Tet1 (#1)_f	AAAAGAAATTAATATTTGAGGGGAAG

Bisulfite	Tet1 (#1)_r	AATAAACCAACCATCCTAAACTAAAC
Bisulfite	Tet1 (#2)_f	GATTTTTATAATTAGAATTTAGAATAGAG
Bisulfite	Tet1 (#2)_r	ATTCATTAATAAAACACTTACTTAAC
Bisulfite	Tet1 (#3)_f	TTTTTTTGGAGATAGATAAGAAGTTA
Bisulfite	Tet1 (#3)_r	AAAATATAAAACACCATTTACAAAACAC
