# Supplementary material for: TET1 is a beige adipocyte-selective epigenetic suppressor of thermogenesis
Source: Nat Commun. 2020 Aug 27;11:4313. doi: 10.1038/s41467-020-18054-y (PMC7453011; doi:10.1038/s41467-020-18054-y)
Supplement: Supplementary file 3 — Reporting Summary [file 41467_2020_18054_MOESM3_ESM.pdf]

natureresearch

Corresponding author(s): Sona Kang

Last updated by author(s): 2020-06/23

Reporting Summary

Nature Research wishes to improve the reproducibility of the work that we publish. This form provides structure for consistency and transparency in reporting. For further information on Nature Research policies, seeAuthors & Referees and theEditorial Policy Checklist .

Statistics

For all statistical analyses, confirm that the following items are present in the figure legend, table legend, main text, or Methods section.

n/a	Confirmed	
☐	☒	The exact sample size (*n*) for each experimental group/condition, given as a discrete number and unit of measurement
☒	☐	A statement on whether measurements were taken from distinct samples or whether the same sample was measured repeatedly
☐	☒	The statistical test(s) used AND whether they are one- or two-sided<br>*Only common tests should be described solely by name; describe more complex techniques in the Methods section.*
☐	☒	A description of all covariates tested
☐	☒	A description of any assumptions or corrections, such as tests of normality and adjustment for multiple comparisons
☐	☒	A full description of the statistical parameters including central tendency (e.g. means) or other basic estimates (e.g. regression coefficient) AND variation (e.g. standard deviation) or associated estimates of uncertainty (e.g. confidence intervals)
☐	☒	For null hypothesis testing, the test statistic (e.g. *F*, *t*, *r*) with confidence intervals, effect sizes, degrees of freedom and *P* value noted<br>*Give P values as exact values whenever suitable.*
☒	☐	For Bayesian analysis, information on the choice of priors and Markov chain Monte Carlo settings
☒	☐	For hierarchical and complex designs, identification of the appropriate level for tests and full reporting of outcomes
☐	☒	Estimates of effect sizes (e.g. Cohen's *d*, Pearson's *r*), indicating how they were calculated

Our web collection on statistics for biologists contains articles on many of the points above.

Software and code

Policy information about availability of computer code

Data collection	No software was used.
Data analysis	Microsoft Excel 2013, Prism 5 and 8, ImageJ1.52, cutadapt ,MethylQA ,Picard tool , makeUCSCfile in UCSC tool kit

For manuscripts utilizing custom algorithms or software that are central to the research but not yet described in published literature, software must be made available to editors/reviewers. We strongly encourage code deposition in a community repository (e.g. GitHub). See the Nature Research guidelines for submitting code & software for further information.

Data

Policy information about availability of data

All manuscripts must include a data availability statement. This statement should provide the following information, where applicable:

- Accession codes, unique identifiers, or web links for publicly available datasets
- A list of figures that have associated raw data
- A description of any restrictions on data availability

Raw data files are available in the source data. Global profiling data are available in the GEO repository under accession number: GSE153093.

Field-specific reporting

Please select the one below that is the best fit for your research. If you are not sure, read the appropriate sections before making your selection.

☒ Life sciences ☐ Behavioural & social sciences ☐ Ecological, evolutionary & environmental sciences

For a reference copy of the document with all sections, see nature.com/documents/nr-reporting-summary-flat.pdf

Life sciences study design

All studies must disclose on these points even when the disclosure is negative.

Sample size	The sample size was large enough to determine statistically significant effects and was determined based upon other studies with similar methodologies (PMID: 29091029 , PMID: 25503565).
Data exclusions	No data were excluded from analysis.
Replication	Experiments were repeated independently at least 2-3 times and successfully reproducible.
Randomization	The groups that receive different experimental treatments are determined randomly.
Blinding	Blind test was performed for data collection and analysis.

Reporting for specific materials, systems and methods

We require information from authors about some types of materials, experimental systems and methods used in many studies. Here, indicate whether each material, system or method listed is relevant to your study. If you are not sure if a list item applies to your research, read the appropriate section before selecting a response.

Materials & experimental systems

n/a	Involved in the study	
☐	☒	Antibodies
☐	☒	Eukaryotic cell lines
☒	☐	Palaeontology
☐	☒	Animals and other organisms
☒	☐	Human research participants
☒	☐	Clinical data

Methods

n/a	Involved in the study	
☒	☐	ChIP-seq
☐	☒	Flow cytometry
☒	☐	MRI-based neuroimaging

Antibodies

Antibodies used	TET1 (GTX124207, 1:1000) from GeneTex. Beta-actin (MA5-14739, 1:1000) from Thermo Fisher. HSP-90 (4877, 1:000), HDAC1 (5356, 1:1000) from CST. Ty1 (A01004, 1:1000) from GenScript. HA (MMS-101R, 1:1000) from Covance. FLAG (F3165, 1:1000) from Sigma. Myc (sc-40, 1:1000) from SCBT. UCP1 (Ab10983, 1:1000), PGC1 (Ab54481, 1:1000), and H3K27ac (Ab4729, 1:1000) from Abcam. CD45-PerCP/Cy5.5 (103131, 1:100), F4/80-PE/Cy7 (123113, 1:100), Cd11b-Pacific Blue (101223, 1:100), and CD301-APC (145707, 1:100) from Biolegend. Cd11c-PE (12-0114-81, 1:100) from eBioscience.
Validation	The validation statements of commercial antibodies were validated using 293T lysates overexpress cDNAs.

Eukaryotic cell lines

Policy information about cell lines

Cell line source(s)	Immortalized mouse beige and brown adipocyte cell lines were obtained from Dr. Shingo Kajimura (UCSF). HEK293T (CRL-1573) was obtained from ATCC.
Authentication	Both cell lines were authenciated by lipid accumulation during adipogenesis and the expression levels of thermogenic gene expression including Ucp1 and Pgc1a by RT-qPCR analysis.
Mycoplasma contamination	Cells were tested to be negative for mycoplasma contamination by PCR screening.
Commonly misidentified lines (See ICLAC register)	None

Animals and other organisms

Policy information about studies involving animals; ARRIVE guidelines recommended for reporting animal research

Laboratory animals	8~16 weeks C57BL/6 wild type male and female mice and Tet1f/f, Fabp4-Cre, PDGFRa-Cre mice backcrossed to C57BL/6 more than 5 generations. Animals were housed at 23 degree.
Wild animals	No wild animals were used in this study.

Field-collected samples	No filed-collected samples were used in this study.
Ethics oversight	All the methods involving live mice were carried out in accordance with the approved guidelines. All experimental protocols were approved by The Animal Care and Use Committee (AUP-2015-08-7887-1) at UC Berkeley.

Note that full information on the approval of the study protocol must also be provided in the manuscript.

Flow Cytometry

Plots

Confirm that:

- [x] The axis labels state the marker and fluorochrome used (e.g. CD4-FITC).
- [x] The axis scales are clearly visible. Include numbers along axes only for bottom left plot of group (a 'group' is an analysis of identical markers).
- [x] All plots are contour plots with outliers or pseudocolor plots.
- [x] A numerical value for number of cells or percentage (with statistics) is provided.

Methodology

Sample preparation	Individual mouse adipose depots were carefully excised and thoroughly minced with scissors (1–2 mm pieces), using sterile techniques. Then, the minced adipose tissue was digested in 0.5U collagenase D (1108874103, 2.5g , Roche, 14253028) in sterile PBS containing 10mM CaCl2 for 30 min in a shaking water bath (100–120 rpm). Floating adipocytes were separated from the SVF by centrifugation at 300 × g for 5 min. The floating adipocyte fraction and supernatant was removed and the SVF pellet was resuspended in PBS 3% BSA wash buffer and sequentially filtered through sterile 70 µm and 40 µm nylon mesh filters before antibody staining. SVF pellet was resuspended in antibody staining solution and placed on ice in the dark for 20 min. After staining, an excess of PBS 3% BSA was added to wash and then the stained cells were centrifuged at 300 × g for 5 min. The wash buffer was carefully removed and the pellet was resuspended in FACS buffer (PBS with 1% BSA). Then. we proceeded to flow cytometry.
Instrument	BD LSR Fortessa (H657675001)
Software	BD FACSDiva 8.0.1
Cell population abundance	Purity of samples was determined by re-sorting the samples. The purity for all the experiment was calculated to be between 85-95 %
Gating strategy	Cell aggregates, dead cells and cellular debris were first excluded at the FSC/SSC gates of the starting cell population. Next, adipose tissue leukocytes (CD45+) were chosen. Then, Adipose tissue macrophages (F4/80-high and CD11b-high) were selected to minimize contamination with eosinophils (F4/80-mid). Finally, viable CD45+F4/80-high/CD11b-high adipose tissue macrophages were analyzed for surface expression of CD11c (M1 marker) and CD301 (M2 marker), which identify discrete M1 and M2 ATM subsets. all gates were defined based on negative controls (wild type mice).

- [x] Tick this box to confirm that a figure exemplifying the gating strategy is provided in the Supplementary Information.
